# The moderating role of impulsivity in the association between sexual abuse and substance use frequency across countries, gender identities, and sexual orientations

**DOI:** 10.1371/journal.pone.0355986

**Published:** 2026-09-30

**Authors:** Damla E. Aksen, Marc N. Potenza, Sarah D. Lichenstein, Léna Nagy, Shane W. Kraus, Joël Billieux, Loïs Fournier, Zsolt Demetrovics, Mónika Koós, International Sex Survey Consortium, Beáta Bőthe

**Affiliations:** 1 Radiology and Biomedical Imaging, Yale School of Medicine, New Haven, Connecticut, United States of America; 2 Yale University School of Medicine, New Haven, Connecticut, United States of America; 3 Connecticut Council on Problem Gambling, Wethersfield, Connecticut, United States of America; 4 Connecticut Mental Health Center, New Haven, Connecticut, United States of America; 5 Department of Psychiatry, Yale School of Medicine, New Haven, Connecticut, United States of America; 6 Doctoral School of Psychology, ELTE Eötvös Loránd University, Budapest, Hungary; 7 Institute of Psychology, ELTE Eötvös Loránd University, Budapest, Hungary; 8 Department of Psychology, University of Nevada, Las Vegas, Nevada, United States of America; 9 Institute of Psychology, University of Lausanne, Lausanne, Switzerland; 10 Center for Excessive Gambling, Addiction Medicine, Lausanne University Hospital, Lausanne, Switzerland; 11 Flinders University Institute for Mental Health and Wellbeing, College of Education, Psychology and Social Work, Flinders University, Bedford Park, South Australia, Australia; 12 Centre of Excellence in Responsible Gaming, University of Gibraltar, Gibraltar, Gibraltar; 13 Institute of Forensic Psychiatry and Sex Research, Center for Translational Neuro-and Behavioral Science, University of Duisburg-Essen, Essen, Germany; 14 Département de Psychologie, Université de Montréal, Montréal, Canada; 15 Centre de recherche interdisciplinaire sur les problèmes conjugaux et les agressions sexuelles (CRIPCAS), Université de Montréal, Montréal, Canada; The University of Alabama, UNITED STATES OF AMERICA

## Abstract

This study examined how impulsivity moderates the relationship between sexual abuse and substance use frequency in a large international sample. Participants (N = 64,472) completed the Alcohol, Smoking, and Substance Involvement Screening Test (ASSIST-11), the UPPS-P Impulsive Behavior Scale, and sexual abuse measures through the International Sex Survey. Structural equation models tested associations between childhood sexual abuse (CSA), adolescent/adult sexual abuse (AASA), and combined exposure with substance use frequency, and explored moderation by impulsivity across gender identity, sexual orientation, and country. AASA and CSA+AASA were consistently associated with higher substance use frequency, while CSA alone showed weaker or inconsistent associations across groups. Urgency and premeditation facets of impulsivity moderated these associations, particularly among sexual and gender minority individuals. Country-level differences indicated cultural and contextual variation in how impulsivity interacts with trauma to influence substance use. Findings underscore the need for targeted, culturally responsive interventions addressing impulsivity in the aftermath of sexual trauma.

Sexual abuse is a robust and well-documented risk factor for substance use and misuse. Individuals who experience sexual abuse during childhood or adolescence are more likely to engage in problematic substance use in adulthood, including heightened risk for dependence, polysubstance use, and earlier initiation of use [1,2]. However, not all survivors of sexual abuse follow this course, suggesting that individual differences, including personality features, are susceptible to moderate associations between trauma and substance-related outcomes. Identifying factors that may influence this heterogeneity is important for improving clinical risk assessment as well as prevention and treatment approaches [3,4].

Impulsivity is a multidimensional construct that contributes importantly to the etiology and maintenance of substance use disorders. Globally defined as a predisposition toward rapid, unplanned reactions to internal or external stimuli with diminished regard to negative consequences [5], impulsivity has been repeatedly associated with more severe use of substances and poor treatment engagement [6,7]. Over the past decades, the UPPS-P Impulsive Behavior Model [8,9] has become a dominant framework to conceptualize impulsivity traits, which has gained traction in both research and clinical practice. Its corresponding assessment tool, the UPPS-P Impulsive Behavior Scale, assesses five distinct aspects of impulsivity: lack of premeditation, lack of perseverance, sensation seeking, negative urgency, and positive urgency [8,9]. These facets exhibit unique associations with various psychopathological symptoms and problematic behaviors, including substance use and misuse, as well as trauma-related behaviors [10,11]. Mood dysregulation and impulsivity have also been found to jointly mediate relationships between early adversity and subsequent substance use [12].

As urgency captures the tendency to act rashly in conditions of intense negative or positive emotion, it may be specifically relevant in trauma-exposed populations, particularly with regard to the facet of negative urgency [3,13,14]. Urgency may potentiate the risk of externalizing behaviors, including substance use, among trauma survivors [3]. Impulsivity may strengthen the relationship between emotional distress and substance use across adolescence [15], suggesting that timing and trajectory may be important considerations. Such relationships resonate with research identifying PTSD symptoms and impulsivity as key contributors to problematic substance use in adolescents with adverse childhood experiences [16]. Yet, while a growing body of literature has examined impulsivity as a mediator or moderator of trauma outcomes, few studies have tested whether specific impulsivity domains interact with distinct forms of sexual abuse, namely, childhood sexual abuse (CSA) and adolescent/adult sexual abuse (AASA) to predict substance use. Bearing in mind that impulsivity is a multi-dimensional construct, tackling the specific role of its various facets is warranted to tailor and improve prevention strategies and treatment options. Moreover, despite increasing recognition of the importance of culturally and demographically responsive research [17], existing studies have rarely considered how such moderation effects may vary across key social identity groups.

Gender identity, sexual orientation, and national context each contribute to shape both trauma exposure and substance use patterns [18,19]. Gender-Minority individuals, for example, report higher rates of both sexual victimization and substance use, yet remain underrepresented in the literature on trauma and behavioral risk [19,20]. Similarly, substance use frequency patterns differ by sexual orientation and vary cross-nationally due to sociopolitical and economic conditions that influence both the prevalence and the meaning of substance use and misuse [21–23]. Understanding whether and how the interaction between trauma and impulsivity facets differs across these groups is important to foster effective and equitable prevention and intervention.

## Aims and objectives

The present study examined impulsivity as a moderating factor in the relationship between sexual abuse history and substance use frequency in a large international sample. The study was preregistered to examine both alcohol- and drug-related outcomes using the Alcohol Use Disorders Identification Test (AUDIT) and the ASSIST-11 (https://osf.io/v3mgx); however, during manuscript preparation, we determined that these measures capture conceptually distinct constructs, as the ASSIST-11 assesses only frequency of substance use whereas the AUDIT assesses both alcohol use and related consequences (e.g., problems, dependence symptoms). Since combining non-comparable dependent variables would reduce interpretability and conceptual clarity, we presented the ASSIST-11-based substance use findings in the current manuscript and report the AUDIT-based alcohol misuse findings in a separate paper. Using latent variable interaction models and the ASSIST-11, we investigated whether UPPS-P impulsivity domains interacted with distinct forms of sexual abuse to statistically predict substance use frequency, and whether these associations varied across gender identity, sexual orientation, and country. By identifying how individual tendencies and social-contextual factors relate to substance use as a function of different sexual abuse contexts, this study aimed to provide a more comprehensive understanding of trauma-related vulnerability

We first evaluated the extent to which different forms of sexual abuse, childhood sexual abuse (CSA), adolescent/adult sexual abuse (AASA), and combined lifetime exposure (CSA+AASA), were associated with patterns of substance use, as assessed by the ASSIST-11. We anticipated that each form of abuse would show significant, positive associations with greater substance involvement.

Next, we examined whether these associations varied based on demographic characteristics, including gender identity, sexual orientation, and country. Drawing on the minority stress theory [24], we hypothesized that sexual and gender minority (SGM) individuals would exhibit stronger associations between sexual abuse and substance use frequency than their cisgender, heterosexual counterparts, given likely heightened exposure to social stressors and cumulative adversity [25].

We also tested the moderating role of impulsivity, particularly urgency-related facets, positive and negative urgency, in abuse/substance-use associations [7,9,26]. Specifically, we hypothesized that individuals with higher levels of positive and negative urgency would show stronger associations between sexual abuse and substance use frequency.

Beyond these primary analyses, we explored whether other impulsivity domains, namely, lack of premeditation, lack of perseverance, and sensation seeking, also moderated these associations across gender-identity and sexual-orientation groups, as well as exploring variation in relationships related to abuse timing (CSA, AASA, or both). Finally, we examined whether the moderating role of impulsivity domains differed across national contexts, with the goal of providing a more globally nuanced understanding of the interplay between sexual abuse, impulsivity, and substance use frequency.

## Methods

### Procedure

Data for the present study were drawn from a completed dataset. The lead author did not have full access to the data prior to preregistration. This project is part of the International Sex Survey (ISS), a multilingual, cross-sectional study conducted across 42 countries and designed to collect self-report data from community-dwelling adults (preregistration link; see Bothe et al., 2021 for the full study protocol). Participant recruitment occurred between October 2021 and May 2022 through various online advertisements. Inclusion criteria required participants to be at or above the legal minimum age of consent in their respective countries (e.g., 18 years in Canada, 20 years in Taiwan); no minors were included. All participants provided informed consent electronically prior to participation. Eligible individuals completed a 25–45-minute anonymous questionnaire administered via Qualtrics. No financial compensation was offered; however, participants could opt to direct a $0.50 donation to one of several international non-profit organizations focused on sexuality and sexual health (donations were capped at $1,000 total). Detailed information about participating countries, translation processes, survey procedures, and eligibility standards is available in the study protocol [27]. Ethics approval for the International Sex Survey (ISS) was obtained from the relevant institutional review boards (IRBs) and ethics committees across all participating sites prior to data collection. In Canada, ethics approval was granted by the Comité d’éthique de la recherche of the Université du Québec à Trois-Rivières and the Comité d’éthique de la recherche en éducation et en psychologie of the Université de Montréal. All participants provided written informed consent electronically prior to completing the survey. A complete list of ethics approvals by country is available at the OSF project page (link to ethics approvals). To promote open science and transparency, a list of all resulting publications and conference presentations is maintained on the OSF project page (publication link; presentation link).

### Sample

Following data cleaning procedures (see link for details), the final ISS dataset comprises responses from 82,243 individuals across 42 countries. For the current analysis, we included only those participants who completed the Sexual Abuse History Questionnaire (SAHQ), the short version of the UPPS-P Impulsive Behavior Scale (SUPPS-P), and the Alcohol, Smoking, and Substance Involvement Screening Test (ASSIST-11), and who were from countries where these instruments demonstrated acceptable psychometric properties. Although the SAHQ and ASSIST-11 showed robust validity and reliability across most participating nations, the SUPPS-P was found to meet psychometric standards in only 26 countries [28–31]. As a result, all analyses for the current study were limited to data from these 26 countries: Australia, Austria, Belgium, Canada, Chile, Colombia, Croatia, France, Germany, Hungary, Ireland, Israel, Italy, Malaysia, Mexico, New Zealand, North Macedonia, Peru, Poland, Portugal, South Africa, South Korea, Spain, Switzerland, the United Kingdom, and the United States [28].

The final sample for the current analyses consisted of N = 64,472 participants across 26 countries (see Table 1). Gender identity was grouped into three categories: cisgender men (n = 23,004), cisgender women (n = 38,136), and gender-minority individuals (n = 3,332). Participants’ sexual orientation was categorized as heterosexual (n = 43,343), gay or lesbian (n = 3,302), and other sexual orientations (n = 17,802). This demographically and geographically diverse dataset offers a unique opportunity for examining how sexual abuse history, impulsivity domains, and substance use frequency intersect across different populations. Summary statistics for key variables are reported in Table 2.

**Table 1:** Sociodemographic Distribution of the Sample (N = 64,472).

Category	Group	n
Country	Australia	639
	Austria	746
	Belgium	644
	Canada	2541
	Chile	1173
	Colombia	1913
	Croatia	2390
	France	1706
	Germany	3271
	Hungary	11200
	Ireland	1702
	Israel	1334
	Italy	2401
	Malaysia	1170
	Mexico	2137
	New Zealand	2834
	North Macedonia	1251
	Peru	2398
	Poland	9892
	Portugal	2262
	South Africa	1849
	South Korea	1464
	Spain	2327
	Switzerland	1144
	United Kingdom	1412
	United States	2398
Gender Identity	Cisgender Men	23004
	Cisgender Women	38136
	Gender Diverse	3332
Sexual Orientation	Gay/Lesbian	3302
	Heterosexual	43343
	Other	17802

**Table 2:** Descriptive Statistics (N = 64,472).

Variable	M	SD	Min	Max
ASSIST-11	3.36	3.36	0	37
UPPS_PR	7.19	2.18	4	16
UPPS_PE	7.72	2.48	4	16
UPPS_SE	9.76	2.69	4	16
UPPS_PU	9.44	2.55	4	16
UPPS_NU	9.06	2.82	4	16

Note. SD = Standard deviation; ASSIST-11 = total score for the Alcohol, Smoking, and Substance Involvement Screening Test (ASSIST-11), ranging from 0 to 40, with higher scores reflecting greater substance involvement across 10 substances in the past three months; UPPS_PR = total score for the UPPS-P Lack of Premeditation subscale (4 items, range: 4–16); UPPS_PE = total score for the UPPS-P Lack of Perseverance subscale (4 items, range: 4–16); UPPS_SE = total score for the UPPS-P Sensation Seeking subscale (4 items, range: 4–16); UPPS_PU = total score for the UPPS-P Positive Urgency subscale (4 items, range: 4–16); UPPS_NU = total score for the UPPS-P Negative Urgency subscale (4 items, range: 4–16). For all UPPS-P subscales, higher scores indicate greater impulsivity on that dimension. All subscales are from the Short version of the UPPS-P Impulsive Behavior Scale (SUPPS-P; Billieux et al., 2012), rated on a 4-point Likert scale (1 = strongly agree to 4 = strongly disagree), with relevant items reverse-scored so that higher scores uniformly reflect higher impulsivity.

### Measures

All included measures and translations can be found at https://osf.io/jcz96/files/osfstorage

#### Sociodemographic characteristics.

Participants provided information on key demographic variables, including age, gender identity, sexual orientation, education level, and country of residence. Country of residence was determined via a single item at the beginning of the survey. Age was assessed by asking participants to report their current age in years. Gender identity was derived from multiple questions capturing sex assigned at birth and current gender identity, and responses were categorized into three groups for analysis: cisgender men, cisgender women, and gender minority individuals. The original preregistration included six distinct gender-identity groups; for the final analysis, these were consolidated into three broader categories to enable meaningful statistical comparisons and maintain adequate sample sizes within each group. Sexual orientation was similarly grouped into three categories: heterosexual, gay or lesbian, and other sexual orientations (including bisexual, pansexual, asexual, queer, and other non-heterosexual, non-gay/lesbian identities). This grouping was necessary to maintain adequate cell sizes for stable structural equation modeling; the original preregistration specified eight sexual orientation categories, but several had insufficient sample sizes for reliable model estimation.

#### Sexual abuse history questionnaire (SAHQ; [32]).

The SAHQ [31,32] is a self-report instrument used to evaluate experiences of sexual abuse during different developmental periods. The measure includes six dichotomous (0 = “no,” 1 = “yes”) items, such as " Has anyone ever exposed the sex organs of their body to you when you did not want it?” and " Has anyone ever threatened to have sex with you when you did not want it?” Participants were categorized into three groups based on their reported abuse history: [1] CSA only, defined as abuse occurring at age 13 or younger; [2] AASA only, defined as abuse beginning at age 14 or older; and [3] CSA + AASA, reflecting abuse reported in both childhood and adolescence/adulthood. The SAHQ demonstrates solid psychometric properties, including convergent validity, test-retest reliability, internal consistency, and measurement invariance across gender-identity-, sexual-orientation-, and country-based contexts [31].

#### Short version of the UPPS-P Impulsive Behavior Scale (SUPPS-P; [33]).

The SUPPS-P [28,33] is a 20-item short form of the UPPS-P Impulsive Behavior Scale assessing five facets of impulsivity, with four items per subscale: lack of premeditation (e.g., ‘I usually think carefully before doing anything’ [reverse-coded]), lack of perseverance (e.g., ‘I finish what I start’ [reverse-coded]), sensation seeking (e.g., ‘I welcome new and exciting experiences and sensations, even if they are a little frightening or unconventional’), negative urgency (e.g., ‘When I feel bad, I often do things I later regret’), and positive urgency (e.g., ‘When I'm in a great mood, I tend to get carried away easily’). Responses are recorded on a 4-point Likert scale… Items for lack of premeditation and lack of perseverance are reverse coded such that higher scores on all subscales uniformly reflect higher impulsivity. Subscale scores range from 4 to 16. The SUPPS-P has demonstrated good convergent validity with established impulsivity measures (e.g., Barratt Impulsiveness Scale, BIS-11), as well as high internal consistency and test-retest reliability [28,33,34].

#### Alcohol, smoking, and substance involvement screening test (ASSIST-11; [35]).

The ASSIST-11 [35] is an 11-item instrument developed by the World Health Organization to assess substance involvement across a range of substances. Participants are asked how often they have used substances such as alcohol, tobacco, cannabis, cocaine, and opioids in the past three months (e.g., “In the past three months, how often have you used cannabis?”). Responses are rated on a 5-point Likert scale from 0 (never) to 4 (daily or almost daily). The final item prompts participants to write in any “other” substances not listed. Higher scores reflect greater levels of substance involvement. The ASSIST-11 has demonstrated strong cross-cultural reliability, convergent validity [36], and internal consistency [30,37].

### Statistical analyses

To address the study’s hypotheses and exploratory aims, we first evaluated measurement invariance for CSA, AASA, and ASSIST-11 variables across country, gender-identity, and sexual-orientation groups. Establishing measurement invariance is essential for confirming that these constructs are interpreted consistently across different groups, enabling valid group comparisons. Given the ordinal nature of the data, we used Diagonally Weighted Least Squares (DWLS) estimation. We tested three levels of invariance: (1) configural invariance, assessing whether the underlying factor structure is consistent across groups; (2) metric invariance, evaluating whether factor loadings are equivalent; and (3) scalar invariance, which tests equality of both factor loadings and item thresholds.

Model fit was assessed using the Comparative Fit Index (CFI), Tucker-Lewis Index (TLI), and Root Mean Square Error of Approximation (RMSEA), following guidelines by Kline (2016). Acceptable model fit was indicated by CFI and TLI ≥ 0.900 and RMSEA ≤ 0.080; optimal fit was defined as CFI and TLI ≥ 0.950 and RMSEA ≤ 0.050 [38–42]. Invariance was judged based on changes in model fit: a CFI decrease ≤ 0.010 and RMSEA increase ≤ 0.015 indicated invariance (Chen, 2007). For comparisons across larger groupings (e.g., > 10 groups), more relaxed criteria were applied, with CFI changes ≤ 0.020 and RMSEA changes ≤ 0.030 [43,44].

Next, we used linear regression models to examine bivariate associations between sexual abuse history (CSA, AASA, CSA + AASA, with no history of sexual victimization as the referent) and substance use frequency (ASSIST-11 scores). Three regression models were estimated with ASSIST-11 as the dependent variable and CSA, AASA, and CSA + AASA as predictors.

We then conducted multi-group structural equation modeling (SEM) to investigate whether the strength of these associations varied across gender-identity and sexual-orientation groups. We compared constrained and unconstrained models to determine if the paths significantly differed by group.

Finally, latent variable interaction models were tested to assess whether impulsivity facets moderated the relationship between sexual abuse history and substance use in the total sample. Interaction terms between each SUPPS-P subscales (e.g., lack of premeditation, sensation seeking) and CSA, AASA, and CSA + AASA were included as predictors of ASSIST-11 scores. These models were first estimated in the total sample and then stratified by gender-identity, sexual-orientation, and country groups to examine whether moderation effects varied across demographic contexts. Where significant interactions were identified, simple slopes analyses were conducted at −1 SD, mean, and +1 SD levels of the moderator to interpret the nature of moderation.

## Results

### Descriptive analyses

The mean ASSIST-11 score was 3.36 (SD = 3.36, range = 0–37). Of the participants, 11.24% had a history of CSA only, 27% had a history of AASA only, and 18.57% had a history of both CSA and AASA (CSA + AASA).

#### Measurement invariance testing and group comparison.

We conducted a series of measurement invariance tests for CSA, AASA, and ASSIST-11 across 26 countries, three sexual-orientation groups, and three gender-identity groups. Due to convergence issues, country-level analyses were divided into two subsets of 13 countries each. Both country-based groups demonstrated good configural fit, but full metric invariance was not achieved. After freeing a small number of item loadings (CSA items 2–3, ASSIST items 2–9 across subsets), partial metric invariance was established. Similarly, partial scalar invariance was required in several cases, achieved by freeing a limited number of intercepts (e.g., CSA items 1–2, AASA item 1, and ASSIST item 2). The number of freed parameters was small relative to the total, supporting the plausibility and parsimony of the partial invariance models. Mean comparisons using Kruskal-Wallis tests revealed significant country-level differences in ASSIST-11 scores, with the highest scores observed in Croatia, New Zealand, and Switzerland. Across sexual-orientation groups, configural and metric invariance were supported, but partial scalar invariance was required. Kruskal-Wallis tests indicated significant group differences, with highest ASSIST-11 scores among individuals identifying with other sexual orientations. For gender identity, full configural, metric, and scalar invariance were achieved. Kruskal-Wallis tests showed significant differences, with gender-minority individuals reporting the highest ASSIST-11 scores. Full fit indices and item-level details are provided in S1 Text and S2 Table.

### Regression analyses

We conducted a series of linear regressions in the SEM framework to examine the associations between sexual abuse history (CSA, AASA, and CSA+AASA, with no history of sexual victimization as the referent) and substance use frequency (ASSIST-11; Table 3). CSA, AASA, and CSA+AASA were each positively associated with substance use frequency, although the variance explained by the models was small (R² < .001). When examining differences by gender identity (Table 4), CSA was associated with lower substance use frequency among cisgender women and gender-minority individuals. In contrast, AASA was associated with higher substance use frequency for cisgender men and cisgender women, but not for gender-minority individuals. CSA+AASA was associated with consistently higher substance use frequency across all gender-identity groups compared to individuals with no history of sexual abuse. Analyses by sexual orientation (Table 5) showed a similar pattern. CSA was associated with lower levels of substance use frequency among all three sexual-orientation groups. AASA was associated with higher substance use frequency across all sexual-orientation groups. Similarly, CSA+AASA was associated with higher substance use frequency across all groups.

**Table 3:** Parameter Estimates from Structural Equation Models Predicting ASSIST-11 Scores.

Model	B	*β*	SE	p	R²
CSA **on** ASSIST**-11**	54.86	0.01	32.96	0.096	<.001
AASA **on** ASSIST**-11**	70.38	0.01	24.38	0.004	<.001
CSA + AASA **on** ASSIST**-11**	6.06	0.001	27.38	0.82	<.001

*Note.* CSA = Childhood Sexual Abuse; AASA = Adult Sexual Abuse; ASSIST-11 = Alcohol, Smoking and Substance Involvement Screening Test.

**Table 4:** Structural Equation Models Predicting ASSIST-11 Scores Across Gender-Identity Groups.

Predictor	Group	B	*β*	SE	*p*
CSA					
	Cisgender Men	−0.10	−0.01	0.08	.160
	Cisgender Women	−0.52	−0.05	0.06	<.001
	Gender-Minority Individuals	−0.92	−0.07	0.23	<.001
AASA					
	Cisgender Men	0.82	0.09	0.06	<.001
	Cisgender Women	0.63	0.09	0.04	<.001
	Gender-Minority Individuals	0.47	0.05	0.16	.003
CSA + AASA					
	Cisgender Men	0.79	0.06	0.09	<.001
	Cisgender Women	0.76	0.10	0.04	<.001
	Gender-Minority Individuals	1.29	0.15	0.15	<.001

*Note.* CSA = Childhood Sexual Abuse; AASA = Adult Sexual Abuse; CSA+AASA = Lifetime Sexual Abuse; ASSIST-11 = Alcohol, Smoking and Substance Involvement Screening Test

**Table 5:** Structural Equation Models Predicting ASSIST-11 Scores Across Sexual-Orientation Groups.

Predictor	Group	B	*β*	SE	*p*
CSA					
	Heterosexual	−0.28	−0.03	0.05	<.001
	Gay/Lesbian	−0.51	−0.04	0.21	.017
	Other Sexual Orientations	−0.63	−0.05	0.09	<.001
AASA					
	Heterosexual	0.53	0.07	0.04	<.001
	Gay/Lesbian	0.83	0.10	0.15	<.001
	Other Sexual Orientations	0.60	0.08	0.06	<.001
CSA + AASA					
	Heterosexual	0.48	0.05	0.04	<.001
	Gay/Lesbian	0.64	0.07	0.17	<.001
	Other Sexual Orientations	0.88	0.11	0.06	<.001

*Note.* CSA = Childhood Sexual Abuse; AASA = Adult Sexual Abuse; ASSIST-11 = Alcohol, Smoking and Substance Involvement Screening Test.

Analyses stratified by country (Table 6) revealed that patterns varied across geographic contexts. CSA was associated with lower levels of substance use frequency in a subset of countries, including Peru, South Africa, and United States of America, although most associations across countries were non-significant. Notably, AASA was associated with higher levels of substance use frequency in almost all countries except for Belgium, Mexico, and Peru. CSA+AASA was generally associated with higher substance use frequency across all countries except for Belgium and United Kingdom.

**Table 6:** Structural Equation Models Predicting ASSIST-11 Scores Across Country Groups.

Predictor	Group	B	*β*	SE	*p*
CSA					
	Australia	−0.60	−0.05	0.55	.280
	Austria	0.21	0.02	0.44	.630
	Belgium	0.74	0.07	0.44	.095
	Canada	−0.55	−0.04	0.26	.033
	Colombia	0.03	0.003	0.19	.870
	Croatia	−0.12	−0.01	0.29	.680
	France	0.20	0.02	0.27	.470
	Germany	0.05	0.01	0.16	.740
	Hungary	−0.05	−0.004	0.11	.640
	Ireland	−0.99	−0.08	0.34	.004
	Israel	−0.40	−0.04	0.32	.210
	Italy	−0.11	−0.01	0.25	.670
	Malaysia	−0.06	−0.01	0.22	.780
	Mexico	−0.27	−0.04	0.17	.110
	New Zealand	−0.28	−0.02	0.27	.300
	North Macedonia	−0.11	−0.01	0.45	.810
	Peru	−0.47	−0.07	0.14	.001
	Poland	−0.27	−0.03	0.11	.012
	Portugal	−0.32	−0.03	0.23	.160
	South Africa	−0.60	−0.05	0.28	.033
	South Korea	−0.17	−0.03	0.18	.350
	Spain	−0.28	−0.02	0.25	.280
	Switzerland	−0.66	−0.06	0.33	.048
	United Kingdom	−0.43	−0.03	0.36	.230
	United States	−0.70	−0.06	0.27	.009
	Chile	−0.16	−0.02	0.30	.590
AASA					
	Australia	0.86	0.10	0.35	.014
	Austria	0.86	0.11	0.29	.003
	Belgium	−0.26	−0.04	0.30	.390
	Canada	0.40	0.05	0.15	.010
	Colombia	0.58	0.07	0.20	.004
	Croatia	0.63	0.08	0.18	<.001
	France	0.47	0.06	0.20	.016
	Germany	0.43	0.06	0.12	<.001
	Hungary	0.60	0.08	0.08	<.001
	Ireland	0.57	0.08	0.20	.004
	Israel	0.55	0.07	0.24	.019
	Italy	0.44	0.06	0.17	.009
	Malaysia	0.77	0.13	0.17	<.001
	Mexico	0.26	0.04	0.16	.099
	New Zealand	0.39	0.05	0.16	.016
	North Macedonia	0.95	0.10	0.28	.001
	Peru	0.13	0.02	0.15	.360
	Poland	0.32	0.05	0.07	<.001
	Portugal	0.65	0.09	0.16	<.001
	South Africa	0.65	0.08	0.20	.001
	South Korea	0.53	0.08	0.18	.003
	Spain	0.93	0.13	0.15	<.001
	Switzerland	0.67	0.09	0.22	.002
	United Kingdom	0.82	0.11	0.21	<.001
	United States	0.55	0.07	0.17	.002
	Chile	0.87	0.11	0.24	<.001
CSA + AASA					
	Australia	1.22	0.14	0.37	<.001
	Austria	1.07	0.11	0.36	.003
	Belgium	0.67	0.08	0.36	.065
	Canada	0.83	0.10	0.18	<.001
	Colombia	0.93	0.11	0.20	<.001
	Croatia	0.86	0.09	0.22	<.001
	France	0.58	0.07	0.21	.007
	Germany	0.47	0.06	0.15	.001
	Hungary	0.67	0.07	0.10	<.001
	Ireland	0.86	0.10	0.22	<.001
	Israel	1.07	0.12	0.26	<.001
	Italy	0.42	0.04	0.21	.045
	Malaysia	0.60	0.10	0.19	.002
	Mexico	0.75	0.12	0.15	<.001
	New Zealand	1.02	0.12	0.17	<.001
	North Macedonia	1.56	0.13	0.36	<.001
	Peru	0.82	0.13	0.13	<.001
	Poland	0.62	0.08	0.08	<.001
	Portugal	1.06	0.13	0.18	<.001
	South Africa	1.02	0.11	0.23	<.001
	South Korea	0.90	0.13	0.19	<.001
	Spain	0.48	0.05	0.21	.020
	Switzerland	0.91	0.10	0.27	.001
	United Kingdom	0.23	0.03	0.23	.310
	United States	1.31	0.16	0.17	<.001
	Chile	1.13	0.13	0.27	<.001

*Note.* CSA = Childhood Sexual Abuse; AASA = Adult Sexual Abuse; CSA+AASA = Lifetime Sexual Abuse; ASSIST-11 = Alcohol, Smoking and Substance Involvement Screening Test.

### Regression analyses by impulsivity domains

We conducted a series of linear regressions in the SEM framework and simple slopes analyses to examine whether impulsivity domains moderated the associations between sexual abuse history and substance use frequency. Results are presented in S3 Table.

Consistent with our hypotheses, both positive and negative urgency significantly moderated the relationship between CSA and substance use frequency (positive urgency: *B* = −0.04, *p* = .015; negative urgency: *B* = −0.05, *p* = .002), such that higher levels of urgency were associated with a stronger positive association. For AASA, only negative urgency emerged as a significant moderator (*B* = 0.03, *p* = .015). In the case of CSA+AASA, both positive (*B* = 0.10, *p* < .001) and negative urgency (*B* = 0.06, *p* < .001) again significantly amplified the association with substance use frequency.

In exploratory analyses, we also examined whether other impulsivity domains moderated abuse/substance-use relationships (see S4 Table). For CSA, lack of premeditation (*B* = −0.04, *p* = .035), lack of perseverance (*B* = −0.06, *p* = .001), and sensation seeking (*B* = −0.04, *p* = .006) all emerged as significant moderators. These effects indicated that higher impulsivity scores were associated with stronger abuse/substance-use associations. These domains did not significantly moderate the AASA/substance-use link (*p*s > .05), but all three significantly moderated the CSA+AASA association: lack of premeditation (*B* = 0.07, *p* < .001), lack of perseverance (*B* = 0.07, *p* < .001), and sensation seeking (*B* = 0.08, *p* < .001).

Simple slopes analyses across all significant interactions revealed that higher levels of impulsivity were associated with stronger associations between sexual abuse and substance use frequency.

#### Gender-identity groups.

Across gender-identity groups, significant moderation effects were most consistently observed among cisgender women and gender-minority individuals (see Table 4). Among cisgender women, all impulsivity domains moderated the association between CSA and substance use frequency, with higher impulsivity associated with more negative associations. For CSA combined with AASA, higher impulsivity across all domains was associated with more positive associations. No significant moderation effects were observed for AASA. Among cisgender men, effects were largely non-significant or inconsistent. Gender-minority individuals showed a mixed pattern, with negative associations observed for CSA and positive associations for CSA+AASA at higher levels of impulsivity.

#### Sexual-orientation groups.

Across sexual-orientation groups, significant moderation effects were most consistently observed among individuals identifying as gay or lesbian and those identifying with other sexual orientations (see Table 5). For CSA, higher impulsivity was generally associated with more negative associations among individuals identifying as gay or lesbian and those with other orientations, particularly for positive urgency and sensation seeking. In contrast, for CSA + AASA, higher impulsivity was associated with more positive associations across multiple impulsivity domains, including among heterosexual, gay or lesbian, and other-identified individuals. AASA showed limited moderation effects across all groups, with most interactions non-significant or inconsistent.

#### Country groups.

Across countries, patterns of moderation varied considerably (see Table 6). For CSA, several countries showed negative associations at higher impulsivity levels, particularly for domains such as positive urgency, sensation seeking, and negative urgency (e.g., Germany, Ireland, and Switzerland). In contrast, for CSA + AASA, a more consistent pattern emerged across countries, with higher impulsivity associated with more positive associations in countries such as the United States, Croatia, and Hungary. Moderation effects for AASA were generally inconsistent and often non-significant across countries.

## Discussion

The present study offers a comprehensive examination of how impulsivity domains moderate the relationships between sexual abuse history and substance use frequency across diverse gender identities, sexual orientations, and jurisdictional contexts. Our findings extend prior research by demonstrating that impulsivity, particularly urgency facets, interact with sexual abuse history in relationships with substance use. These interactions underscore the importance of individual tendencies in understanding heterogeneous outcomes among sexual trauma survivors and align with prior work identifying impulsivity as a mechanism linking trauma to substance use [4].

Consistent with the existing literature, findings suggested that individuals higher in impulsivity, especially those with elevated negative or positive urgency, were more likely to show stronger associations between sexual abuse and substance use frequency. This aligns with prior work suggesting that impulsivity may influence substance use as a potential coping strategy for trauma-related emotional distress [3]. Urgency, which reflects tendencies to act rashly under intense emotional states, appears especially relevant for understanding maladaptive behavioral responses following trauma [9,10] and has been conceptualized as a key mechanism linking emotional states to substance use [45].

However, some results diverged from established findings. CSA was not consistently associated with higher substance use; in several groups, including cisgender women, gender-minority individuals, and certain country and sexual-orientation groups, CSA was negatively associated with substance use frequency. These results contrast with the substantial literature linking CSA to increased substance use risk [1,2]. Such discrepancies may reflect the moderating role of protective factors (e.g., resilience, social support), differential use severity, or underreporting biases, particularly in culturally or socially stigmatized settings or variation in the availability and accessibility of psychoactive substances across groups and regions. Several additional non-mutually exclusive mechanisms may account for this seemingly counterintuitive finding. First, the ASSIST-11 captures frequency of use in the past three months rather than lifetime patterns, abuse-related onset, or severity of dependence; CSA survivors who engaged in heavy substance use at an earlier developmental stage may not be in an active period of use at the time of survey completion, or their use patterns may have shifted in ways not captured by a frequency-based measure [30,35]. Second, CSA survivors may develop internalizing rather than externalizing coping styles, particularly when the abuse occurred early in development and was never disclosed, such that distress may be expressed through outcomes other than frequent substance use [3,26,46]. Third, individuals who experienced CSA without subsequent AASA may represent a relatively resilient subgroup who were able to access protective resources, such as social support or treatment [47]. Fourth, the online recruitment strategy may have underrepresented the most severely affected survivors, who may be less likely to participate in a voluntary survey. Importantly, these findings should not be interpreted as evidence that CSA is protective against substance use; the robust epidemiological literature documents positive associations between CSA and lifetime substance use risk [2], and the present cross-sectional, frequency-based findings may reflect measurement, sampling, and contextual factors rather than a true protective effect. Critically, impulsivity is not itself a coping strategy but rather a dispositional feature that may moderate in which coping behaviors individuals engage when under emotional distress [9,45]. Individuals high in urgency may be more likely to resort to substance use as a maladaptive response to trauma-related distress, a pattern more consistent with the positive moderation effects observed for AASA and CSA+AASA, where the accumulation of abuse experiences may more reliably trigger such emotion-driven substance use [12].

Importantly, the moderating effects of impulsivity domains varied by abuse type. For example, while CSA and CSA+AASA showed consistent moderation across all five UPPS-P domains, AASA demonstrated limited and inconsistent moderation effects. This suggests that impulsivity traits may exert greater influence on substance use behavior when trauma occurs earlier in life or accumulates over time. Developmental timing and chronicity of trauma may therefore shape the behavioral expression of impulsive tendencies [15,48].

Subgroup analyses suggested that gender and sexual orientation shaped associations between sexual abuse, impulsivity, and substance use frequency. Among cisgender women and gender-minority individuals, these associations were more consistently moderated by impulsivity facets. These findings align with prior research suggesting that women may be particularly vulnerable to substance use following trauma [2], and that gender-minority individuals often encounter additional stressors, including identity-related discrimination, which may intensify behavioral consequences of trauma [19,20]. Individuals identifying as gay, lesbian, or with other non-heterosexual orientations also exhibited more frequent and pronounced associations between sexual abuse, impulsivity, and substance use. These observations are consistent with the Minority Stress Model, which proposes that sexual minorities experience chronic psychosocial stress due to stigma and discrimination, contributing to elevated health risks [21,24]. When combined with documented emotional distress and difficulties in emotion regulation among sexual minority individuals generally [46], and sexual minority women specifically [49], these stressors may contribute to a heightened likelihood of substance use within these communities [50].

Jurisdictional contexts may further shape relationships. While impulsivity domains moderated the relationship between sexual abuse and substance use frequency in many countries, the pattern was more consistent in the United States, where CSA+AASA and high impulsivity jointly statistically predicted greater substance use frequency. Conversely, moderation patterns were inconsistent in countries like Belgium and the United Kingdom. These variations may reflect sociocultural differences in trauma disclosure, substance availability, stigma, mental health infrastructures, or other factors [22]. These cross-national differences highlight the importance of situating findings within specific country contexts when designing interventions [23].

Together, these findings suggest the importance of trauma-informed, culturally responsive approaches to substance use prevention and intervention. Findings suggest that clinicians should be particularly attentive to impulsivity features, especially negative and positive urgency, when working with trauma-exposed individuals. Incorporating emotion-regulation strategies into treatment (e.g., Dialectical Behavior Therapy skills such as distress tolerance and emotion regulation, or mindfulness-based relapse prevention) may be especially effective for those with high impulsivity [7]. Moreover, for sexual and gender minority populations, addressing minority stress through supportive services and affirming environments may help mitigate compounding risk factors [19,24]. These speculative possibilities warrant direct examination.

Several limitations should be considered when interpreting the findings. Although the large, diverse sample is a strength, the study is subject to both general limitations of the International Sex Survey (ISS; https://osf.io/6kscb) and issues specific to this analysis. The study’s cross-sectional design precludes causal inference. All variables were self-reported, raising concerns about recall and social desirability bias. Additionally, convergence issues during confirmatory factor analysis required us to split the country-level sample, which may reflect instability in measurement across cultural groups. A further limitation concerns the aggregation of sexual orientation categories. By grouping bisexual, pansexual, and other non-heterosexual, non-gay/lesbian identities into a single “other” category, the present analyses may obscure meaningful differences between these subgroups. Bisexual individuals, in particular, have been shown to experience elevated rates of both sexual victimization and substance use relative to gay or lesbian individuals [21]. Future studies with larger and more representative samples should examine these subgroups separately to capture potential heterogeneity in risk. Another limitation concerns the assessment of substance use, as the ASSIST-11 focuses on frequency of use but does not capture substance-related problems, consequences, or symptom severity. This limitation suggests that the findings may underestimate the clinical significance of substance-use concerns. Future research should employ longitudinal designs, include more comprehensive assessments of substance-related severity, and incorporate qualitative methods to explore the lived experiences behind these statistical associations.

## Conclusions

In conclusion, this study highlights the complex interplay between sexual abuse, impulsivity, and substance use across diverse populations. By identifying specific subgroups and psychological mechanisms that shape these associations, this work contributes to a more nuanced understanding of individual and structural vulnerability and can inform more tailored, effective public health responses.

^24^Macedonian Academy of Sciences and Arts, Republic of North Macedonia
^25^Institute of Psychlogy, Polish Academy of Sciences, Poland
^28^Department of Psychology, University of New Mexico, Albuquerque, USA
^29^University of Baghdad, College of Medicine, Iraq
^42^Chuncheon Addiction Management Center, South Korea
^43^Centre of Excellence in Responsible Gaming, University of Gibraltar, Gibraltar
^52^Facultad de Medicina, Universidad Científica del Sur, Lima, Perú
^53^College of Healthcare Sciences, James Cook University, Australia
^54^Artois University, France
^55^Department of Psychology, Sungkyunkwan University, South Korea

## Supporting information

S1 Text Measurement invariance testing and group comparisons for CSA, AASA, and ASSIST-11. Full narrative results of configural, metric, and scalar invariance testing and Kruskal-Wallis group comparisons by country, gender identity, and sexual orientation.
(DOCX)

S2 Table Measurement invariance results for CSA, AASA, and ASSIST-11 across 26 countries, three gender-identity groups, and three sexual-orientation groups. Includes configural, metric, and scalar invariance fit indices, item-level details for all measurement models, and country-based pairwise comparisons from Kruskal-Wallis tests on the ASSIST-11 and SAHQ (Child, Adult, and full-scale) subscales.
(DOCX)

S3 Table Latent variable interaction models and simple slopes analyses for all UPPS-P impulsivity domains by abuse type (CSA, AASA, CSA+AASA) in the full sample, stratified by gender-identity group, and stratified by sexual-orientation group. (DOCX)

S4 Table Exploratory latent variable interaction models examining lack of premeditation, lack of perseverance, and sensation seeking as moderators of abuse/substance-use associations by abuse type and demographic subgroup. (DOCX)
