# Supplementary material for: The moderating role of impulsivity in the association between sexual abuse and substance use frequency across countries, gender identities, and sexual orientations
Source: PLoS One. 2026 Sep 30;21(9):e0355986. doi: 10.1371/journal.pone.0355986 (PMC13626376; doi:10.1371/journal.pone.0355986)
Supplement: Supplementary file 1 [file pone.0355986.s001.docx]

**S1 Text. Measurement invariance testing and group comparisons for CSA, AASA, and ASSIST-11. Full narrative results of configural, metric, and scalar invariance testing and Kruskal-Wallis group comparisons by country, gender identity, and sexual orientation.**

First, we conducted measurement invariance testing to evaluate the factor structure of CSA across 26 countries, three sexual orientation groups, and three gender identity groups. The initial configural model demonstrated a good fit across the 26 countries (CFI = 0.996, TLI = 0.993, RMSEA = 0.014, 90% CI [0.010, 0.017]), indicating consistent factor structure across groups. In the metric invariance stage, changes in CFI, TLI, and RMSEA exceeded the recommended thresholds (ΔCFI = -0.029, ΔTLI = -0.028, ΔRMSEA = 0.018). To address this, we adjusted the factor loadings for item 2 and item 3 based on modification indices, achieving partial metric invariance with adequate changes in fit indices (ΔCFI = -0.019, ΔTLI = -0.021, ΔRMSEA = 0.015). Similarly, when evaluating scalar invariance, changes in CFI, TLI, and RMSEA also exceeded thresholds (ΔCFI = -0.028, ΔTLI = -0.018, ΔRMSEA = 0.008). Consequently, we adjusted the intercepts for item 1 and item 2, resulting in partial scalar invariance with acceptable changes in fit indices (ΔCFI = -0.015, ΔTLI = -0.011, ΔRMSEA = 0.005). Mean scores on CSA were compared with Kruskal-Wallis tests by country (*H*(25) = 1388.262, *p < .*001). The highest scores on the CSA were observed in Mexico (*M* = 1.15, *SD =* 1.56), followed by Peru (*M* = .97, *SD* = 1.46) and Australia (*M* = .90, *SD* = 1.62).

Next, we assessed measurement invariance across three sexual orientation groups (i.e., heterosexual, gay, lesbian or bisexual, and other). The initial configural model indicated a good fit (CFI = 0.992, TLI = 0.987, RMSEA = 0.020, 90% CI [0.018, 0.022]). Changes in fit indices suggested that scalar invariance was achieved, indicating no mean differences between sexual orientation groups. Mean scores on CSA were compared with Kruskal-Wallis tests by sexual orientation (*H*(2) = 1217.48, *p < .*001). The highest scores on the CSA were observed among individuals who identified as Gay, Lesbian or Bisexual (*M* = 0.87, *SD =* 1.53), followed by other (*M* = 0.84, *SD* = 1.40) and Heterosexual (*M* = 0.51, *SD* = 1.08).

Finally, we conducted measurement invariance testing across three gender-based groups (i.e., cis men, cis women, and Gender Diverse Individuals). The initial configural model demonstrated a good fit (CFI = 0.992, TLI = 0.987, RMSEA = 0.019, 90% CI [0.018, 0.022]). Although metric invariance was achieved, scalar invariance was not. To address this, we adjusted the intercepts for item 1 and item 6, resulting in partial scalar invariance with adequate changes in the fit indices (ΔCFI = -0.007, ΔTLI = -0.005, ΔRMSEA = 0.003). Mean scores on CSA were compared with Kruskal-Wallis tests by gender-based group (*H*(2) = 2179.58, *p < .*001). The highest scores on CSA were observed in Gender Diverse Individuals (*M* = 1.08, *SD =* 1.63), followed by Women (*M* = .70, *SD* = 1.24) and Men (*M* = .44, *SD* = 1.07).

We applied the same sequence of invariance tests for AASA across 26 countries, three sexual orientation groups, and three gender identity groups. The initial configural model for the AASA across 26 countries demonstrated a good fit (CFI = 0.997, TLI = 0.995, RMSEA = 0.016, 90% CI [0.013, 0.019]). Changes in fit indices indicated that both metric invariance (ΔCFI = -0.013, ΔTLI = -0.012, ΔRMSEA = 0.015) and scalar invariance (ΔCFI = -0.020, ΔTLI = -0.012, ΔRMSEA = 0.010) were achieved, suggesting no mean differences between countries. Mean scores on the AASA were compared with Kruskal-Wallis tests by country (*H*(25) = 2122.76, *p < .*001). The highest scores on the AASA were observed in New Zealand (*M* = 1.73, *SD* = 1.96), followed by Ireland (*M* = 1.68, *SD* = 1.84) and Canada (*M* = 1.62, *SD* = 1.89).

Similarly, we tested measurement invariance across three sexual orientation groups for AASA. The initial configural model indicated a good fit (CFI = 0.996, TLI = 0.993, RMSEA = 0.020, 90% CI [0.018, 0.023]). Changes in fit indices confirmed the achievement of both metric invariance (ΔCFI = -0.003, ΔTLI = -0.001, ΔRMSEA = 0.001) and scalar invariance (ΔCFI = -0.005, ΔTLI = -0.003, ΔRMSEA = 0.004), indicating no mean differences between sexual orientation groups. Mean scores on AASA were compared with Kruskal-Wallis tests by sexual orientation (*H*(2) = 3358.64, *p < .*001). The highest scores on the AASA were observed among individuals who identified as belonging to other sexual orientation groups (*M* = 1.55, *SD =* 1.77), followed by individuals who identify as Gay, Lesbian or Bisexual (*M* = 1.16, *SD* = 1.58) and Heterosexual (*M* = .82, *SD* = 1.35).

In the final step for AASA, we tested measurement invariance across three gender-based groups. The initial configural model demonstrated a good fit (CFI = 0.995, TLI = 0.992, RMSEA = 0.021, 90% CI [0.019, 0.023]). While metric invariance was achieved (ΔCFI = -0.005, ΔTLI = -0.004, ΔRMSEA = 0.005), scalar invariance was not. We adjusted the intercept for item 1, resulting in partial scalar invariance with acceptable changes in fit indices (ΔCFI = -0.010, ΔTLI = -0.008, ΔRMSEA = 0.007). Mean scores on the AASA were compared with Kruskal-Wallis tests by gender-based group (*H*(2) = 7899.26, *p < .*001). The highest scores on the AASA were observed in the Gender-diverse group (*M* = 1.71, *SD* = 1.93), followed by Women (*M* = 1.35, *SD* = 1.64) and Men (*M* = 0.48, *SD* = 1.02).

For ASSIST, we followed a similar sequence of invariance tests across 26 countries, three sexual orientation groups, and three gender identity groups. However, due to convergence issues when analyzing all 26 countries together, we divided the countries into two groups, each consisting of 13 countries, and conducted a single invariance test for each group. For the first group, the initial configural model demonstrated a good fit (CFI = 0.941, TLI = 0.924, RMSEA = 0.027, 90% CI [0.0285 0.029]). For metric invariance, changes in CFI, TIL and RMSEA exceeded recommended thresholds (ΔCFI = -0.113, ΔTLI = -0.102, ΔRMSEA = 0.015) necessitating adjustments to the factor loadings for items ASSIST5, ASSIST8, ASSIST4, ASSIST3, ASSIST6, and ASSIST2. This led to partial metric invariance with acceptable changes in fit indices (ΔCFI = -0.016 ΔTLI = -0.014, ΔRMSEA = 0.003). Changes in fit indices confirmed that scalar invariance was achieved (ΔCFI = 0.003 ΔTLI = -0.002, ΔRMSEA = 0). Similarly, for the second group, the initial configural model demonstrated a good fit (CFI = 0.950, TLI = 0.934, RMSEA = 0.029, 90% CI [0.027 0.031]). However, for metric invariance, changes in CFI, TIL and RMSA exceeded recommended thresholds (ΔCFI = -109, ΔTLI = -0.101, ΔRMSEA = 0.017) requiring adjustments to the factor loadings of items ASSIST9, ASSIST8, ASSIST4, ASSIST3, ASSIST5 and ASSIST7. Changes in CFI, TIL and RMSA confirmed that scalar invariance was achieved (ΔCFI = -0.02 ΔTLI = -0.015, ΔRMSEA = 0.003). Mean scores on the ASSIST scale were compared with Kruskal-Wallis tests by country (*H*(25) = 3233.47, *p < .*001). The highest scores on the ASSIST scale were observed in Croatia (*M* = 4.72, *SD =* 3.78), followed by New Zealand (*M* = 4.01, *SD* = 3.73) and Switzerland (*M* = 3.96, *SD* = 3.32).

After the country-based tests, we conducted measurement invariance testing across three sexual orientation groups for the ASSIST. The initial configural model demonstrated adequate fit (CFI = 0.938, TLI = 0.920, RMSEA = 0.029, 90% CI [0.0328, 0.030]). Changes in fit indices suggested that metric invariance (ΔCFI = -0.009, ΔTLI = 0.002, ΔRMSEA = 0) was achieved, but scalar invariance was not (ΔCFI = -0.017, ΔTLI = -0.007, ΔRMSEA = 0.010). We addressed this by adjusting the intercept for item ASSIST2, resulting in partial scalar invariance with adequate changes in fit indices (ΔCFI = -0.01, ΔTLI = -0.001, ΔRMSEA = 0). Mean scores on ASSIST were compared with Kruskal-Wallis tests by sexual orientation (*H*(2) = 737.37, *p < .*001). The highest scores on the ASSIST were observed among individuals who identified as belonging to other sexual orientation groups (*M* = 3.70, *SD =* 3.67), followed by individuals who identify as Gay, Lesbian or Bisexual (*M* = 3.56, *SD* = 3.75) and Heterosexual (*M* = 2.88, *SD* = 3.17).

Finally, we conducted measurement invariance testing across three gender-based groups for ASSIST. The initial configural model demonstrated a good fit (CFI = 0.973, TLI = 0.966, RMSEA = 0.039, 90% CI [0.038, 0.040]). Changes in fit indices indicated that both metric invariance (ΔCFI = -0.004, ΔTLI = 0, ΔRMSEA = 0) and scalar invariance (ΔCFI = -0.009, ΔTLI = -0.004, ΔRMSEA = 0.003) were achieved, suggesting no mean differences between gender-based groups. Mean scores on ASSIST were compared with Kruskal-Wallis tests by gender-based group (*H*(2) = 8.80, *p = .*012). The highest scores on ASSIST were observed for the Gender-diverse group (*M* = 3.47, *SD* = 3.93), followed by Men (*M* = 3.14, *SD* = 3.41) and Women (*M* = 3.10, *SD* = 3.27).
