# Supplementary material for: The moderating role of impulsivity in the association between sexual abuse and substance use frequency across countries, gender identities, and sexual orientations
Source: PLoS One. 2026 Sep 30;21(9):e0355986. doi: 10.1371/journal.pone.0355986 (PMC13626376; doi:10.1371/journal.pone.0355986)
Supplement: Supplementary file 2 [file pone.0355986.s002.docx]

Table A. Country-based Confirmatory Factor Analyses and Tests of Invariance on the Sexual Abuse History Questionnaire – Child Subscale (SAHQ)

| Model | WLSMV χ^2^ | df | | CFI | TLI | RMSEA | 90% CI | Comp. | Δdf | ΔCFI | ΔTLI | ΔRMSEA |
| --- | --- | --- | --- | --- | --- | --- | --- | --- | --- | --- | --- | --- |
| Australia | 4.981 | | 9 | 1 | 1.009 | 0 | 0- 0.027 | — | — | — | — | — |
| Austria | 23.239 | | 9 | 0.910 | 0.850 | 0.048 | 0.024- 0.073 | — | — | — | — | — |
| Belgium | 5.332 | | 9 | 1 | 1.019 | 0 | 0- 0.029 | — | — | — | — | — |
| Canada | 11.705 | | 9 | 0.998 | 0.997 | 0.011 | 0- 0.027 | — | — | — | — | — |
| Chile | 8.568 | | 9 | 1 | 1.001 | 0 | 0- 0.032 | — | — | — | — | — |
| Colombia | 18.535 | | 9 | 0.991 | 0.985 | 0.024 | 0.007- 0.041 | — | — | — | — | — |
| Croatia | 3.745 | | 9 | 1 | 1.013 | 0 | 0- 0.007 | — | — | — | — | — |
| France | 10.589 | | 9 | 0.998 | 0.997 | 0.010 | 0- 0.030 | — | — | — | — | — |
| Germany | 16.571 | | 9 | 0.990 | 0.984 | 0.016 | 0- 0.029 | — | — | — | — | — |
| Hungary | 34.985 | | 9 | 0.986 | 0.976 | 0.016 | 0.010- 0.022 | — | — | — | — | — |
| Ireland | 9.032 | | 9 | 0.999 | 0.999 | 0.001 | 0- 0.028 | — | — | — | — | — |
| Israel | 4.327 | | 9 | 1 | 1.017 | 0 | 0- 0.015 | — | — | — | — | — |
| Italy | 8.415 | | 9 | 1 | 1.001 | 0 | 0- 0.022 | — | — | — | — | — |
| Malaysia | 11.464 | | 9 | 0.994 | 0.991 | 0.015 | 0- 0.039 | — | — | — | — | — |
| Mexico | 25.980 | | 9 | 0.992 | 0.987 | 0.031 | 0.017- 0.046 | — | — | — | — | — |
| New Zealand | 10.927 | | 9 | 0.999 | 0.998 | 0.009 | 0- 0.025 | — | — | — | — | — |
| North Macedonia | 2.608 | | 9 | 1 | 1.046 | 0 | 0-0 | — | — | — | — | — |
| Peru | 21.408 | | 9 | 0.994 | 0.990 | 0.023 | 0.010- 0.037 | — | — | — | — | — |
| Poland | 32.135 | | 9 | 0.987 | 0.979 | 0.016 | 0.010- 0.023 | — | — | — | — | — |
| Portugal | 11.558 | | 9 | 0.996 | 0.994 | 0.011 | 0- 0.028 | — | — | — | — | — |
| South Africa | 8.675 | | 9 | 1 | 1.000 | 0 | 0- 0.026 | — | — | — | — | — |
| South Korea | 23.026 | | 9 | 0.977 | 0.963 | 0.034 | 0.017- 0.052 | — | — | — | — | — |
| Spain | 9.844 | | 9 | 0.998 | 0.997 | 0.006 | 0- 0.025 | — | — | — | — | — |
| Switzerland | 4.186 | | 9 | 1 | 1.022 | 0 | 0- 0.015 | — | — | — | — | — |
| United Kingdom | 7.488 | | 9 | 1 | 1.003 | 0 | 0- 0.027 | — | — | — | — | — |
| United States of America | 13.584 | | 9 | 0.998 | 0.997 | 0.015 | 0- 0.030 | — | — | — | — | — |
| M1. Configural | 342.918 | | 234 | 0.996 | 0.993 | 0.014 | 0.010- 0.017 | — | — | — | — | — |
| M2. Metric | 1246.164 | | 359 | 0.967 | 0.965 | 0.032 | 0.030- 0.034 | M2-M1 | 125 | -0.029 | -0.028 | 0.018 |
| M2a. Metric Partial ^a^ | 1028.002 | | 334 | 0.974 | 0.970 | 0.030 | 0.027- 0.032 | M2a-M1 | -25 | -0.022 | -0.023 | 0.016 |
| M2b. Metric Partial ^b^ | 931.295 | | 309 | 0.977 | 0.972 | 0.029 | 0.027- 0.031 | M2b-M1 | -25 | -0.019 | -0.021 | 0.015 |
| M3. Scalar | 1823.211 | | 434 | 0.949 | 0.954 | 0.037 | 0.035- 0.039 | M3-M2b | 125 | -0.028 | -0.018 | 0.008 |
| M3a. Scalar Partial ^c^ | 1622.3 | | 409 | 0.956 | 0.958 | 0.035 | 0.034- 0.037 | M3a-M2b | -25 | -0.021 | -0.014 | 0.006 |
| M3b. Scalar Partial ^d^ | 1431.999 | | 384 | 0.962 | 0.961 | 0.034 | 0.032- 0.036 | M3b-M2b | -25 | -0.015 | -0.011 | 0.005 |

*Note*. WLSMV = weighted least squares mean- and variance-adjusted estimator; χ^2^ = Chi-square; df = degrees of freedom; CFI = comparative fit index; TLI = Tucker-Lewis Index; RMSEA = root-mean-square error of approximation; 90% CI = 90% confidence interval of the RMSEA; Comp. = Comparison; ΔCFI = change in CFI value compared to the preceding model; ΔTLI = change in the TLI value compared to the preceding model; ΔRMSEA = change in the RMSEA value compared to the preceding model. a = Factor loading for item SAHQ2_c was allowed to vary between groups. b = In addition to the previous model, factor loading for item SAHQ3_c was allowed to vary between groups. c = The intercept for item SAHQ1_c was freed to vary between groups. d = In addition to the previous model, the intercept for item SAHQ2_c was freed to vary between groups.

Table B. Country-based Confirmatory Factor Analyses and Tests of Invariance on the Sexual Abuse History Questionnaire – Adult Subscale (SAHQ)

| Model | WLSMV χ^2^ | df | | CFI | TLI | RMSEA | 90% CI | Comp. | Δdf | ΔCFI | ΔTLI | ΔRMSEA |
| --- | --- | --- | --- | --- | --- | --- | --- | --- | --- | --- | --- | --- |
| Australia | 3.123 | | 9 | 1 | 1.006 | 0 | 0-0 | — | — | — | — | — |
| Austria | 8.477 | | 9 | 1 | 1.001 | 0 | 0- 0.041 | — | — | — | — | — |
| Belgium | 5.288 | | 9 | 1 | 1.015 | 0 | 0- 0.029 | — | — | — | — | — |
| Canada | 15.503 | | 9 | 0.998 | 0.997 | 0.017 | 0- 0.031 | — | — | — | — | — |
| Chile | 14.773 | | 9 | 0.989 | 0.983 | 0.024 | 0- 0.045 | — | — | — | — | — |
| Colombia | 11.720 | | 9 | 0.996 | 0.994 | 0.013 | 0- 0.032 | — | — | — | — | — |
| Croatia | 14.432 | | 9 | 0.996 | 0.994 | 0.016 | 0- 0.031 | — | — | — | — | — |
| France | 7.540 | | 9 | 1 | 1.001 | 0 | 0- 0.024 | — | — | — | — | — |
| Germany | 19.838 | | 9 | 0.994 | 0.990 | 0.020 | 0.007- 0.032 | — | — | — | — | — |
| Hungary | 54.867 | | 9 | 0.990 | 0.984 | 0.022 | 0.016- 0.028 | — | — | — | — | — |
| Ireland | 12.710 | | 9 | 0.998 | 0.997 | 0.016 | 0- 0.035 | — | — | — | — | — |
| Israel | 4.955 | | 9 | 1 | 1.006 | 0 | 0- 0.018 | — | — | — | — | — |
| Italy | 12.414 | | 9 | 0.997 | 0.996 | 0.013 | 0- 0.029 | — | — | — | — | — |
| Malaysia | 8.273 | | 9 | 1 | 1.001 | 0 | 0- 0.031 | — | — | — | — | — |
| Mexico | 19.918 | | 9 | 0.993 | 0.989 | 0.025 | 0.010- 0.041 | — | — | — | — | — |
| New Zealand | 19.011 | | 9 | 0.998 | 0.997 | 0.021 | 0.007- 0.034 | — | — | — | — | — |
| North Macedonia | 5.830 | | 9 | 1 | 1.008 | 0 | 0- 0.023 | — | — | — | — | — |
| Peru | 5.926 | | 9 | 1 | 1.003 | 0 | 0- 0.016 | — | — | — | — | — |
| Poland | 51.417 | | 9 | 0.992 | 0.986 | 0.022 | 0.016- 0.028 | — | — | — | — | — |
| Portugal | 12.747 | | 9 | 0.996 | 0.994 | 0.014 | 0- 0.030 | — | — | — | — | — |
| South Africa | 10.873 | | 9 | 0.998 | 0.998 | 0.011 | 0- 0.030 | — | — | — | — | — |
| South Korea | 7.623 | | 9 | 1 | 1.001 | 0 | 0- 0.027 | — | — | — | — | — |
| Spain | 16.229 | | 9 | 0.994 | 0.990 | 0.019 | 0- 0.033 | — | — | — | — | — |
| Switzerland | 10.536 | | 9 | 0.998 | 0.997 | 0.012 | 0- 0.038 | — | — | — | — | — |
| United Kingdom | 11.821 | | 9 | 0.998 | 0.997 | 0.015 | 0- 0.037 | — | — | — | — | — |
| United States of America | 16.884 | | 9 | 0.998 | 0.997 | 0.019 | 0- 0.034 | — | — | — | — | — |
| M1. Configural | 382.741 | | 234 | 0.997 | 0.995 | 0.016 | 0.013- 0.019 | — | — | — | — | — |
| M2. Metric | 1190.772 | | 359 | 0.984 | 0.983 | 0.031 | 0.029- 0.033 | M2-M1 | 125 | -0.013 | -0.012 | 0.015 |
| M3. Scalar | 2386.457 | | 484 | 0.964 | 0.971 | 0.041 | 0.039- 0.043 | M3-M2 | 125 | -0.02 | -0.012 | 0.01 |

*Note*. WLSMV = weighted least squares mean- and variance-adjusted estimator; χ^2^ = Chi-square; df = degrees of freedom; CFI = comparative fit index; TLI = Tucker-Lewis Index; RMSEA = root-mean-square error of approximation; 90% CI = 90% confidence interval of the RMSEA; Comp. = Comparison; ΔCFI = change in CFI value compared to the preceding model; ΔTLI = change in the TLI value compared to the preceding model; ΔRMSEA = change in the RMSEA value compared to the preceding model.

Table C. Country-based Confirmatory Factor Analyses and Tests of Invariance on the Alcohol, Smoking and Substance Involvement Screening Test (ASSIST)

| Model | WLSMV χ^2^ | df | CFI | TLI | RMSEA | 90% CI | Comp. | Δdf | ΔCFI | ΔTLI | ΔRMSEA |  |
| --- | --- | --- | --- | --- | --- | --- | --- | --- | --- | --- | --- | --- |
| Group 1 | | | | | | | | | | | |  |
| Australia | 40.862 | 35 | 0.977 | 0.872 | 0.018 | 0-0.037 | — | — | — | — | — |  |
| Austria | 46.797 | 35 | 0.958 | 0.946 | 0.023 | 0-0.385 | — | — | — | — | — |  |
| Belgium | 57.648 | 35 | 0.875 | 0.839 | 0.334 | 0.017-0.049 | — | — | — | — | — |  |
| Canada | 72.665 | 35 | 0.947 | 0.932 | 0.022 | 0.015-0.030 | — | — | — | — | — |  |
| Chile | 56.978 | 35 | 0.968 | 0.959 | 0.024 | 0.012-0.036 | — | — | — | — | — |  |
| Colombia | 82.728 | 35 | 0.948 | 0.934 | 0.028 | 0.020-0.036 | — | — | — | — | — |  |
| Croatia | 124.330 | 35 | 0.925 | 0.904 | 0.036 | 0.029-0.043 | — | — | — | — | — |  |
| France | 82.155 | 35 | 0.922 | 0.900 | 0.031 | 0.022-0.039 | — | — | — | — | — |  |
| Germany | 89.821 | 35 | 0.914 | 0.890 | 0.023 | 0.017-0.029 | — | — | — | — | — |  |
| Hungary | 309.631 | 35 | 0.925 | 0.904 | 0.029 | 0.026-0.032 | — | — | — | — | — |  |
| Ireland | 57.293 | 35 | 0.975 | 0.968 | 0.022 | 0.011-0.031 | — | — | — | — | — |  |
| Israel | 54.528 | 35 | 0.969 | 0.961 | 0.022 | 0.009-0.034 | — | — | — | — | — |  |
| Italy | 84.409 | 35 | 0.962 | 0.951 | 0.027 | 0.020-0.034 | — | — | — | — | — |  |
| M1. Configural | 1159.85 | 455 | 0.941 | 0.924 | 0.027 | 0.025-0.029 | — | — | — | — | — |  |
| M2. Metric | 2618.87 | 563 | 0.828 | 0.822 | 0.042 | 0.040-0.044 | M2-M1 | 108 | -0.113 | -0.102 | 0.015 |  |
| M2a. Metric Partial ^a^ | 2325.48 | 551 | 0.852 | 0.843 | 0.039 | 0.038-0.041 | M2a-M1 | 96 | -0.089 | -0.081 | 0.012 |  |
| M2b. Metric Partial ^b^ | 2037.26 | 539 | 0.875 | 0.864 | 0.037 | 0.035- 0.038 | M2b-M1 | 84 | -0.066 | -0.06 | 0.01 |  |
| M2c. Metric Partial ^c^ | 1824.01 | 527 | 0.892 | 0.880 | 0.034 | 0.033- 0.036 | M2c-M1 | 72 | -0.049 | -0.044 | 0.007 |  |
| M2d. Metric Partial ^d^ | 1653.40 | 515 | 0.905 | 0.892 | 0.033 | 0.031- 0.034 | M2d-M1 | 60 | -0.036 | -0.032 | 0.006 |  |
| M2e. Metric Partial ^e^ | 1479.36 | 503 | 0.919 | 0.905 | 0.031 | 0.028- 0.032 | M2e-M1 | 48 | -0.022 | -0.019 | 0.004 |  |
| M2f. Metric Partial ^f^ | 1394.14 | 491 | 0.925 | 0.910 | 0.030 | 0.028- 0.032 | M2f-M1 | 36 | -0.016 | -0.014 | 0.003 |  |
| M3. Scalar | 868.94 | 35 | 0.928 | 0.908 | 0.030 | 0.028- 0.031 | M3-M2f | -456 | 0.003 | -0.002 | 0 |  |
| Group 2 | | | | | | | | | | | |  |
| Malaysia | 33.78 | 27 | 0.967 | 0.956 | 0.015 | 0- 0.030 | — | — | — | — | — |  |
| Mexico | 64.845 | 27 | 0.947 | 0.930 | 0.028 | 0.020- 0.037 | — | — | — | — | — |  |
| New Zealand | 73.877 | 27 | 0.944 | 0.928 | 0.027 | 0.020- 0.035 | — | — | — | — | — |  |
| North Macedonia | 93.419 | 27 | 0.942 | 0.923 | 0.048 | 0.037- 0.058 | — | — | — | — | — |  |
| Peru | 68.963 | 27 | 0.957 | 0.943 | 0.026 | 0.019- 0.034 | — | — | — | — | — |  |
| Poland | 235.676 | 27 | 0.948 | 0.931 | 0.030 | 0.027- 0.034 | — | — | — | — | — |  |
| Portugal | 67.627 | 27 | 0.952 | 0.935 | 0.028 | 0.020- 0.037 |  |  |  |  |  |  |
| South Africa | 82.659 | 27 | 0.935 | 0.913 | 0.037 | 0.028- 0.046 | — | — | — | — | — |  |
| South Korea | 34.421 | 27 | 0.975 | 0.967 | 0.014 | 0- 0.027 | — | — | — | — | — |  |
| Spain | 83.138 | 27 | 0.950 | 0.933 | 0.032 | 0.024- 0.040 | — | — | — | — | — |  |
| Switzerland | 45.313 | 27 | 0.963 | 0.951 | 0.026 | 0.012- 0.039 | — | — | — | — | — |  |
| United Kingdom | 53.801 | 27 | 0.955 | 0.939 | 0.029 | 0.017- 0.040 | — | — | — | — | — |  |
| United States of America | 54.572 | 27 | 0.964 | 0.951 | 0.022 | 0.014-0.031 |  |  |  |  |  |  |
| M1. Configural | 992.08 | 351 | 0.950 | 0.934 | 0.029 | 0.027-0.031 | — | — | — | — | — |  |
| M2. Metric | 2496.48 | 447 | 0.841 | 0.833 | 0.046 | 0.045-0.048 | M2-M1 | 96 | -0.109 | -0.101 | 0.017 |  |
| M2g. Metric Partial ^g^ | 2233.79 | 435 | 0.860 | 0.849 | 0.044 | 0.042-0.046 | M2g-M1 | 84 | -0.09 | -0.085 | 0.015 |  |
| M2h. Metric Partial ^h^ | 2026.42 | 423 | 0.875 | 0.862 | 0.042 | 0.040-0.044 | M2h-M1 | 72 | -0.075 | -0.072 | 0.013 |  |
| M2i. Metric Partial ^i^ | 1869.21 | 411 | 0.887 | 0.871 | 0.041 | 0.039-0.043 | M2i-M1 | 60 | -0.063 | -0.063 | 0.012 |  |
| M2j. Metric Partial ^j^ | 1527.97 | 399 | 0.912 | 0.897 | 0.036 | 0.034-0.038 | M2j-M1 | 48 | -0.038 | -0.037 | 0.007 |  |
| M2k. Metric Partial ^k^ | 1296.98 | 387 | 0.929 | 0.914 | 0.033 | 0.031-0.035 | M2k-M1 | 36 | -0.021 | -0.02 | 0.004 |  |
| M2l Metric Partial ^l^ | 1183.11 | 375 | 0.937 | 0.922 | 0.031 | 0.030-0.034 | M2l-M1 | 24 | -0.013 | -0.012 | 0.002 |  |
| M3m. Scalar Partial ^m^ | 2085.17 | 64 | 0.917 | 0.907 | 0.034 | 0.033-0.035 | M3-M2l | -311 | -0.02 | -0.015 | 0.003 |  |

*Note*. WLSMV = weighted least squares mean- and variance-adjusted estimator; χ^2^ = Chi-square; df = degrees of freedom; CFI = comparative fit index; TLI = Tucker-Lewis Index; RMSEA = root-mean-square error of approximation; 90% CI = 90% confidence interval of the RMSEA; Comp. = Comparison; ΔCFI = change in CFI value compared to the preceding model; ΔTLI = change in the TLI value compared to the preceding model; ΔRMSEA = change in the RMSEA value compared to the preceding model. a = The intercept for item ASSIST5 was freed to vary between groups. b = In addition to the previous model, the intercept for item ASSIST8 was freed to vary between groups. c = In addition to the previous model, the intercept for item ASSIST4 was freed to vary between groups. d = In addition to the previous model, the intercept for item ASSIST3 was freed to vary between groups. e = In addition to the previous model, the intercept for item ASSIST6 was freed to vary between groups. f = In addition to the previous model, the intercept for item ASSIST2 was freed to vary between groups. g = The intercept for item ASSIST9 was freed to vary between groups. h = In addition to the previous model, the intercept for item ASSIST8 was freed to vary between groups. i = In addition to the previous model, the intercept for item ASSIST4 was freed to vary between groups. j = In addition to the previous model, the intercept for item ASSIST3 was freed to vary between groups. k = In addition to the previous model, the intercept for item ASSIST5 was freed to vary between groups. l = In addition to the previous model, the intercept for item ASSIST7 was freed to vary between groups.

`

Table D. Sexual Orientation Confirmatory Factor Analyses and Tests of Invariance on the Sexual Abuse History Questionnaire – Child Subscale (SAHQ)

| Model | WLSMV χ^2^ | df | | CFI | TLI | RMSEA | 90% CI | Comp. | Δdf | ΔCFI | ΔTLI | ΔRMSEA |
| --- | --- | --- | --- | --- | --- | --- | --- | --- | --- | --- | --- | --- |
| Heterosexual | 173.966 | | 9 | 0.990 | 0.985 | 0.018 | 0.016- 0.021 | — | — | — | — | — |
| Gay, Lesbian or Bisexual | 30.609 | | 9 | 0.995 | 0.991 | 0.024 | 0.015- 0.033 | — | — | — | — | — |
| Other Sexual Orientations | 94.426 | | 9 | 0.994 | 0.989 | 0.022 | 0.018- 0.026 | — | — | — | — | — |
| M1. Configural | 299.001 | | 27 | 0.992 | 0.987 | 0.020 | 0.018- 0.022 | — | — | — | — | — |
| M2. Metric | 553.153 | | 37 | 0.986 | 0.982 | 0.023 | 0.021- 0.025 | M2-M1 | 10 | -0.006 | -0.005 | 0.003 |
| M3. Scalar | 780.045 | | 47 | 0.980 | 0.980 | 0.025 | 0.023- 0.026 | M3-M2 | 10 | -0.002 | -0.002 | 0.002 |

*Note*. WLSMV = weighted least squares mean- and variance-adjusted estimator; χ^2^ = Chi-square; df = degrees of freedom; CFI = comparative fit index; TLI = Tucker-Lewis Index; RMSEA = root-mean-square error of approximation; 90% CI = 90% confidence interval of the RMSEA; Comp. = Comparison; ΔCFI = change in CFI value compared to the preceding model; ΔTLI = change in the TLI value compared to the preceding model; ΔRMSEA = change in the RMSEA value compared to the preceding model.

Table E. Sexual Orientation Confirmatory Factor Analyses and Tests of Invariance on the Sexual Abuse History Questionnaire – Adult Subscale (SAHQ)

| Model | WLSMV χ^2^ | df | | CFI | TLI | RMSEA | 90% CI | Comp. | Δdf | ΔCFI | ΔTLI | ΔRMSEA |
| --- | --- | --- | --- | --- | --- | --- | --- | --- | --- | --- | --- | --- |
| Heterosexual | 167.444 | | 9 | 0.995 | 0.992 | 0.019 | 0.016- 0.021 | — | — | — | — | — |
| Gay, Lesbian or Bisexual | 47.350 | | 9 | 0.991 | 0.985 | 0.032 | 0.023- 0.041 | — | — | — | — | — |
| Other Sexual Orientations | 93.211 | | 9 | 0.997 | 0.995 | 0.022 | 0.018- 0.026 | — | — | — | — | — |
| M1. Configural | 308.005 | | 27 | 0.996 | 0.993 | 0.020 | 0.018- 0.023 | — | — | — | — | — |
| M2. Metric | 458.712 | | 37 | 0.993 | 0.992 | 0.021 | 0.020- 0.023 | M2-M1 | 10 | -0.003 | -0.001 | 0.001 |
| M3. Scalar | 774.174 | | 47 | 0.988 | 0.989 | 0.025 | 0.023- 0.026 | M3-M2 | 10 | -0.005 | -0.003 | 0.004 |

*Note*. WLSMV = weighted least squares mean- and variance-adjusted estimator; χ^2^ = Chi-square; df = degrees of freedom; CFI = comparative fit index; TLI = Tucker-Lewis Index; RMSEA = root-mean-square error of approximation; 90% CI = 90% confidence interval of the RMSEA; Comp. = Comparison; ΔCFI = change in CFI value compared to the preceding model; ΔTLI = change in the TLI value compared to the preceding model; ΔRMSEA = change in the RMSEA value compared to the preceding model.

Table F. Sexual Orientation Confirmatory Factor Analyses and Tests of Invariance on the Alcohol, Smoking and Substance Involvement Screening Test (ASSIST)

| Model | WLSMV χ^2^ | df | | CFI | TLI | RMSEA | 90% CI | Comp. | Δdf | ΔCFI | ΔTLI | ΔRMSEA |
| --- | --- | --- | --- | --- | --- | --- | --- | --- | --- | --- | --- | --- |
| Heterosexual | 1248.138 | | 35 | 0.939 | 0.921 | 0.027 | 0.025- 0.028 | — | — | — | — | — |
| Gay, Lesbian or Bisexual | 213.446 | | 35 | 0.928 | 0.907 | 0.036 | 0.032- 0.041 | — | — | — | — | — |
| Other Sexual Orientations | 642.196 | | 35 | 0.943 | 0.927 | 0.031 | 0.029- 0.033 | — | — | — | — | — |
| M1. Configural | 2103.781 | | 105 | 0.939 | 0.922 | 0.028 | 0.027- 0.029 | — | — | — | — | — |
| M2. Metric | 2811.204 | | 123 | 0.918 | 0.910 | 0.030 | 0.029- 0.031 | M2-M1 | 18 | -0.021 | -0.012 | 0.002 |
| M2a. Metric Partial ^a^ | 2659.222 | | 121 | 0.923 | 0.914 | 0.030 | 0.029- 0.031 | M2a-M1 | 16 | -0.016 | -0.008 | 0.002 |
| M2b. Metric Partial ^b^ | 2548.789 | | 119 | 0.926 | 0.916 | 0.029 | 0.028- 0.030 | M2b-M1 | 14 | -0.013 | -0.006 | 0.001 |
| M2c. Metric Partial ^c^ | 2475.993 | | 117 | 0.928 | 0.917 | 0.029 | 0.028- 0.030 | M2c-M1 | 12 | -0.011 | -0.005 | 0.001 |
| M2d. Metric Partial ^d^ | 2435.275 | | 115 | 0.930 | 0.917 | 0.029 | 0.028- 0.030 | M2d-M1 | 10 | -0.009 | -0.005 | 0.001 |
| M3. Scalar | 3011.504 | | 133 | 0.913 | 0.911 | 0.030 | 0.029- 0.031 | M3-M2d | 18 | -0.017 | -0.006 | 0.001 |
| M3a. Scalar Partial ^e^ | 2848.846 | | 131 | 0.918 | 0.915 | 0.030 | 0.029- 0.030 | M3a-M2d | 16 | -0.012 | -0.002 | 0.001 |
| M3b. Scalar Partial ^f^ | 2781.609 | | 129 | 0.920 | 0.916 | 0.030 | 0.029- 0.031 | M3b-M2d | 14 | -0.01 | -0.001 | 0.001 |

*Note*. WLSMV = weighted least squares mean- and variance-adjusted estimator; χ^2^ = Chi-square; df = degrees of freedom; CFI = comparative fit index; TLI = Tucker-Lewis Index; RMSEA = root-mean-square error of approximation; 90% CI = 90% confidence interval of the RMSEA; Comp. = Comparison; ΔCFI = change in CFI value compared to the preceding model; ΔTLI = change in the TLI value compared to the preceding model; ΔRMSEA = change in the RMSEA value compared to the preceding model. a = Factor loading for item ASSIST2 was allowed to vary between groups. b = In addition to the previous model, factor loading for item ASSIST6 was allowed to vary between groups. c = In addition to the previous model, factor loading for item ASSIST8 was allowed to vary between groups. d = In addition to the previous model, factor loading for item ASSIST3 was allowed to vary between groups. e = The intercept for item ASSIST3 was freed to vary between groups. f = In addition to the previous model, the intercept for item ASSIST1 was freed to vary between groups

Table G. Gender Identity Based Confirmatory Factor Analyses and Tests of Invariance on the Sexual Abuse History Questionnaire – Child Subscale (SAHQ)

| Model | WLSMV χ^2^ | df | | CFI | TLI | RMSEA | 90% CI | Comp. | Δdf | ΔCFI | ΔTLI | ΔRMSEA |
| --- | --- | --- | --- | --- | --- | --- | --- | --- | --- | --- | --- | --- |
| Cis Men | 90.362 | | 9 | 0.993 | 0.988 | 0.017 | 0.014- 0.021 | — | — | — | — | — |
| Cis Women | 176.780 | | 9 | 0.991 | 0.986 | 0.021 | 0.018- 0.024 | — | — | — | — | — |
| Gender Diverse Individuals | 28.295 | | 9 | 0.996 | 0.993 | 0.024 | 0.014- 0.034 | — | — | — | — | — |
| M1. Configural | 295.437 | | 27 | 0.992 | 0.987 | 0.019 | 0.018- 0.022 | — | — | — | — | — |
| M2. Metric | 592.248 | | 37 | 0.984 | 0.981 | 0.024 | 0.023- 0.026 | M2-M1 | 10 | -0.008 | -0.006 | 0.005 |
| M3. Scalar | 1474.933 | | 47 | 0.960 | 0.961 | 0.035 | 0.033- 0.036 | M3-M2 | 10 | -0.024 | -0.02 | 0.011 |
| M3a. Scalar Partial ^a^ | 1173.345 | | 45 | 0.968 | 0.968 | 0.032 | 0.029- 0.033 | M3a-M2 | 8 | -0.016 | -0.013 | 0.008 |
| M3b. Scalar Partial ^b^ | 859.130 | | 43 | 0.977 | 0.976 | 0.027 | 0.025- 0.029 | M3b-M2 | 6 | -0.007 | -0.005 | 0.003 |

*Note*. WLSMV = weighted least squares mean- and variance-adjusted estimator; χ^2^ = Chi-square; df = degrees of freedom; CFI = comparative fit index; TLI = Tucker-Lewis Index; RMSEA = root-mean-square error of approximation; 90% CI = 90% confidence interval of the RMSEA; Comp. = Comparison; ΔCFI = change in CFI value compared to the preceding model; ΔTLI = change in the TLI value compared to the preceding model; ΔRMSEA = change in the RMSEA value compared to the preceding model. a = The intercept for item SAHQ1_c was freed to vary between groups. b = In addition to the previous model, the intercept for item SAHQ6_c was freed to vary between groups.

Table H. Gender Identity Based Confirmatory Factor Analyses and Tests of Invariance on the Sexual Abuse History Questionnaire – Adult Subscale (SAHQ)

| Model | WLSMV χ^2^ | df | | CFI | TLI | RMSEA | 90% CI | Comp. | Δdf | ΔCFI | ΔTLI | ΔRMSEA |
| --- | --- | --- | --- | --- | --- | --- | --- | --- | --- | --- | --- | --- |
| Cis Men | 91.072 | | 9 | 0.991 | 0.985 | 0.018 | 0.014-0.021 | — | — | — | — | — |
| Cis Women | 193.324 | | 9 | 0.996 | 0.993 | 0.022 | 0.019-0.024 | — | — | — | — | — |
| Gender Diverse Individuals | 27.959 | | 9 | 0.998 | 0.996 | 0.024 | 0.014-0.034 | — | — | — | — | — |
| M1. Configural | 312.356 | | 27 | 0.995 | 0.992 | 0.021 | 0.019-0.023 | — | — | — | — | — |
| M2. Metric | 649.939 | | 37 | 0.990 | 0.988 | 0.026 | 0.024-0.027 | M2-M1 | 10 | -0.005 | -0.004 | 0.005 |
| M3. Scalar | 1853.188 | | 47 | 0.970 | 0.971 | 0.039 | 0.037-0.041 | M3-M2 | 10 | -0.02 | -0.017 | 0.013 |
| M3a. Scalar Partial ^a^ | 1264.948 | | 45 | 0.980 | 0.980 | 0.033 | 0.031-0.034 | M3a-M2 | 8 | -0.01 | -0.008 | 0.007 |

*Note*. WLSMV = weighted least squares mean- and variance-adjusted estimator; χ^2^ = Chi-square; df = degrees of freedom; CFI = comparative fit index; TLI = Tucker-Lewis Index; RMSEA = root-mean-square error of approximation; 90% CI = 90% confidence interval of the RMSEA; Comp. = Comparison; ΔCFI = change in CFI value compared to the preceding model; ΔTLI = change in the TLI value compared to the preceding model; ΔRMSEA = change in the RMSEA value compared to the preceding model. a = The intercept for item SAHQ1_a was freed to vary between groups.

Table I. Gender Identity Based Confirmatory Factor Analyses and Tests of Invariance on the Alcohol, Smoking and Substance Involvement Screening Test (ASSIST)

| Model | WLSMV χ^2^ | df | | CFI | TLI | RMSEA | 90% CI | Comp. | Δdf | ΔCFI | ΔTLI | ΔRMSEA |
| --- | --- | --- | --- | --- | --- | --- | --- | --- | --- | --- | --- | --- |
| Cis Men | 942.685 | | 35 | 0.927 | 0.907 | 0.031 | 0.029- 0.033 | — | — | — | — | — |
| Cis Women | 1100.978 | | 35 | 0.945 | 0.929 | 0.027 | 0.026- 0.029 | — | — | — | — | — |
| Gender Diverse Individuals | 169.313 | | 35 | 0.939 | 0.921 | 0.033 | 0.027- 0.038 | — | — | — | — | — |
| M1. Configural | 2212.976 | | 105 | 0.938 | 0.920 | 0.029 | 0.028- 0.030 | — | — | — | — | — |
| M2. Metric | 2540.643 | | 123 | 0.929 | 0.922 | 0.029 | 0.028- 0.030 | M2-M1 | 18 | -0.009 | 0.002 | 0 |
| M3. Scalar | 3145.115 | | 141 | 0.912 | 0.915 | 0.030 | 0.029- 0.031 | M3-M2 | 18 | -0.017 | -0.007 | 0.01 |
| M3a. Scalar Partial ^a^ | 2909.288 | | 139 | 0.919 | 0.921 | 0.029 | 0.028- 0.030 | M3a-M2 | 16 | -0.01 | -0.001 | 0 |

*Note*. WLSMV = weighted least squares mean- and variance-adjusted estimator; χ^2^ = Chi-square; df = degrees of freedom; CFI = comparative fit index; TLI = Tucker-Lewis Index; RMSEA = root-mean-square error of approximation; 90% CI = 90% confidence interval of the RMSEA; Comp. = Comparison; ΔCFI = change in CFI value compared to the preceding model; ΔTLI = change in the TLI value compared to the preceding model; ΔRMSEA = change in the RMSEA value compared to the preceding model. a = The intercept for item ASSIST2 was freed to vary between groups.

Table J. Country Based Confirmatory Factor Analyses and Tests of Invariance on UPPS-P Impulsive Behavior Scale

| Model | WLSMV χ^2^ | df | | CFI | TLI | RMSEA | 90% CI | Comp. | Δdf | ΔCFI | ΔTLI | ΔRMSEA |
| --- | --- | --- | --- | --- | --- | --- | --- | --- | --- | --- | --- | --- |
| Australia | 627.052 | | 165 | 0.947 | 0.939 | 0.066 | 0.061-0.072 | — | — | — | — | — |
| Austria | 470.290 | | 165 | 0.965 | 0.959 | 0.050 | 0.044-0.055 | — | — | — | — | — |
| Belgium | 835.640 | | 165 | 0.907 | 0.893 | 0.080 | 0.074-0.085 | — | — | — | — | — |
| Canada | 1637.059 | | 165 | 0.959 | 0.952 | 0.059 | 0.057-0.062 | — | — | — | — | — |
| Chile | 1224.831 | | 165 | 0.910 | 0.896 | 0.074 | 0.070-0.078 | — | — | — | — | — |
| Colombia | 2510.425 | | 165 | 0.850 | 0.827 | 0.087 | 0.084-0.090 | — | — | — | — | — |
| Croatia | 2462.529 | | 165 | 0.922 | 0.910 | 0.077 | 0.074-0.079 | — | — | — | — | — |
| France | 1498.245 | | 165 | 0.929 | 0.918 | 0.069 | 0.066-0.072 | — | — | — | — | — |
| Germany | 2205.024 | | 165 | 0.936 | 0.927 | 0.061 | 0.059-0.064 | — | — | — | — | — |
| Hungary | 11031.9 | | 165 | 0.922 | 0.911 | 0.077 | 0.075-0.078 | — | — | — | — | — |
| Ireland | 1453.039 | | 165 | 0.940 | 0.930 | 0.070 | 0.064-0.071 | — | — | — | — | — |
| Israel | 1277.196 | | 165 | 0.933 | 0.923 | 0.071 | 0.068-0.075 | — | — | — | — | — |
| Italy | 2306.705 | | 165 | 0.927 | 0.916 | 0.074 | 0.071-0.076 | — | — | — | — | — |
| Malaysia | 1270.57 | | 165 | 0.904 | 0.890 | 0.076 | 0.072-0.080 | — | — | — | — | — |
| Mexico | 1791.579 | | 165 | 0.934 | 0.924 | 0.068 | 0.065-0.071 | — | — | — | — | — |
| New Zealand | 2460.689 | | 165 | 0.941 | 0.932 | 0.070 | 0.068-0.073 | — | — | — | — | — |
| North Macedonia | 1946.49 | | 165 | 0.860 | 0.839 | 0.094 | 0.090-0.097 | — | — | — | — | — |
| Peru | 2779.179 | | 165 | 0.905 | 0.891 | 0.077 | 0.075-0.080 | — | — | — | — | — |
| Poland | 5588.586 | | 165 | 0.951 | 0.943 | 0.057 | 0.056-0.059 | — | — | — | — | — |
| Portugal | 1850.651 | | 165 | 0.928 | 0.917 | 0.073 | 0.070-0.076 | — | — | — | — | — |
| South Africa | 1772.203 | | 165 | 0.934 | 0.924 | 0.073 | 0.070-0.076 | — | — | — | — | — |
| South Korea | 2475.328 | | 165 | 0.899 | 0.885 | 0.098 | 0.095-0.101 | — | — | — | — | — |
| Spain | 2400.365 | | 165 | 0.908 | 0.894 | 0.076 | 0.074-0.079 | — | — | — | — | — |
| Switzerland | 885.791 | | 165 | 0.947 | 0.939 | 0.062 | 0.058-0.066 | — | — | — | — | — |
| United Kingdom | 1286.645 | | 165 | 0.941 | 0.931 | 0.069 | 0.066-0.073 | — | — | — | — | — |
| United States of America | 2075.22 | | 165 | 0.948 | 0.940 | 0.070 | 0.067-0.072 | — | — | — | — | — |
| M1. Configural | 58123.23 | | 4290 | 0.930 | 0.919 | 0.072 | 0.071-0.072 | — | — | — | — | — |
| M2. Metric | 72834.64 | | 4765 | 0.911 | 0.908 | 0.076 | 0.076-0.077 | M2-M1 | 475 | -0.019 | -0.011 | 0.004 |
| M2a. Metric Partial ^a^ | 70476.42 | | 4740 | 0.914 | 0.910 | 0.075 | 0.075- 0.076 | M2a-M1 | 450 | -0.016 | -0.009 | 0.004 |
| M2b. Metric Partial ^b^ | 69802.39 | | 4715 | 0.915 | 0.911 | 0.075 | 0.075- 0.075 | M2b-M1 | 425 | -0.015 | -0.008 | 0 |
| M2c. Metric Partial ^c^ | 67124.83 | | 4690 | 0.918 | 0.914 | 0.073 | 0.073- 0.074 | M2c-M1 | 400 | -0.012 | -0.005 | 0.002 |
| M2d. Metric Partial ^d^ | 65161.71 | | 4665 | 0.921 | 0.916 | 0.072 | 0.072- 0.073 | M2d-M1 | 375 | -0.009 | -0.003 | 0.001 |
| M3. Scalar | 89564.98 | | 4890 | 0.919 | 0.915 | 0.072 | 0.072-0.072 | M3-M2d | 225 | -0.002 | -0.001 | 0 |

*Note*. WLSMV = weighted least squares mean- and variance-adjusted estimator; χ^2^ = Chi-square; df = degrees of freedom; CFI = comparative fit index; TLI = Tucker-Lewis Index; RMSEA = root-mean-square error of approximation; 90% CI = 90% confidence interval of the RMSEA; Comp. = Comparison; ΔCFI = change in CFI value compared to the preceding model; ΔTLI = change in the TLI value compared to the preceding model; ΔRMSEA = change in the RMSEA value compared to the preceding model. a = Factor loading for item UPPS11 was allowed to vary between groups. b = In addition to the previous model, factor loading for item UPPS7R was allowed to vary between groups. c = In addition to the previous model, factor loading for item UPPS_SE was allowed to vary between groups. d = In addition to the previous model, factor loading for item UPPS20R was allowed to vary between groups.

Table K. Country-based Pairwise Comparisons in the Kruskal-Wallis Test on the Alcohol, Smoking and Substance Involvement Screening Test (ASSIST)

| **Country comparisons** | **Test statistic** | **Standard error** | **Standardized test statistic**  **(z)** | ***p*-value** | **Adjusted *p*-value ^a^** |
| --- | --- | --- | --- | --- | --- |
| Malaysia-South Korea | -3607.536 | 667.254 | -5.407 | <.001 | <.001 |
| Malaysia-Colombia | 4471.892 | 627.831 | 7.123 | <.001 | <.001 |
| Malaysia-Peru | -6067.414 | 600.658 | -10.101 | <.001 | <.001 |
| Malaysia-Mexico | -7547.082 | 624.212 | -12.091 | <.001 | <.001 |
| Malaysia-Israel | 11046.448 | 688.26 | 16.05 | <.001 | <.001 |
| Malaysia-Belgium | 11206.991 | 839.447 | 13.35 | <.001 | <.001 |
| Malaysia-Chile | -11303.798 | 702.492 | -16.091 | <.001 | <.001 |
| Malaysia-Portugal | -11371.428 | 615.703 | -18.469 | <.001 | <.001 |
| Malaysia-Germany | 11472.407 | 577.081 | 19.88 | <.001 | <.001 |
| Malaysia-France | 12379.086 | 647.996 | 19.104 | <.001 | <.001 |
| Malaysia-Australia | 12461.145 | 839.447 | 14.844 | <.001 | <.001 |
| Malaysia-United States of America | -13002.108 | 607.916 | -21.388 | <.001 | <.001 |
| Malaysia-Spain | -13002.617 | 610.264 | -21.307 | <.001 | <.001 |
| Malaysia-United Kingdom | -14064.911 | 676.89 | -20.779 | <.001 | <.001 |
| Malaysia-Poland | -14293.416 | 524.854 | -27.233 | <.001 | <.001 |
| Malaysia-North Macedonia | -14295.227 | 691.15 | -20.683 | <.001 | <.001 |
| Malaysia-South Africa | -14700.815 | 640.114 | -22.966 | <.001 | <.001 |
| Malaysia-Italy | 15022.959 | 612.451 | 24.529 | <.001 | <.001 |
| Malaysia-Ireland | 15069.164 | 654.551 | 23.022 | <.001 | <.001 |
| Malaysia-Hungary | 15448.569 | 521.088 | 29.647 | <.001 | <.001 |
| Malaysia-Austria | 15628.228 | 796.83 | 19.613 | <.001 | <.001 |
| Malaysia-New Zealand | -15889.643 | 593.112 | -26.79 | <.001 | <.001 |
| Malaysia-Canada | 16118.776 | 601.717 | 26.788 | <.001 | <.001 |
| Malaysia-Switzerland | -16434.989 | 704.654 | -23.323 | <.001 | <.001 |
| Malaysia-Croatia | 19397.48 | 608.785 | 31.863 | <.001 | <.001 |
| South Korea-Colombia | 864.356 | 591.606 | 1.461 | 0.144 | 1.000 |
| South Korea-Peru | 2459.878 | 562.686 | 4.372 | *<.001* | 0.004 |
| South Korea-Mexico | 3939.546 | 587.763 | 6.703 | *<.001* | <.001 |
| South Korea-Israel | 7438.912 | 655.384 | 11.35 | *<.001* | <.001 |
| South Korea-Belgium | 7599.455 | 812.71 | 9.351 | *<.001* | <.001 |
| South Korea-Chile | -7696.262 | 670.314 | -11.482 | *<.001* | <.001 |
| South Korea-Portugal | 7763.892 | 578.719 | 13.416 | *<.001* | <.001 |
| South Korea-Germany | 7864.871 | 537.446 | 14.634 | *<.001* | <.001 |
| South Korea-France | 8771.55 | 612.964 | 14.31 | *<.001* | <.001 |
| South Korea-Australia | 8853.609 | 812.71 | 10.894 | *<.001* | <.001 |
| South Korea-United States of America | -9394.572 | 570.428 | -16.469 | *<.001* | <.001 |
| South Korea-Spain | -9395.081 | 572.929 | -16.398 | *<.001* | <.001 |
| South Korea-United Kingdom | -10457.375 | 643.433 | -16.252 | *<.001* | <.001 |
| South Korea-Poland | 10685.88 | 480.933 | 22.219 | *<.001* | <.001 |
| South Korea-North Macedonia | 10687.691 | 658.418 | 16.232 | *<.001* | <.001 |
| South Korea-South Africa | 11093.279 | 604.625 | 18.347 | *<.001* | <.001 |
| South Korea-Italy | 11415.423 | 575.258 | 19.844 | *<.001* | <.001 |
| South Korea-Ireland | 11461.628 | 619.889 | 18.49 | *<.001* | <.001 |
| South Korea-Hungary | 11841.033 | 476.821 | 24.833 | *<.001* | <.001 |
| South Korea-Austria | 12020.692 | 768.612 | 15.639 | *<.001* | <.001 |
| South Korea-New Zealand | 12282.107 | 554.624 | 22.145 | *<.001* | <.001 |
| South Korea-Canada | 12511.24 | 563.816 | 22.19 | *<.001* | <.001 |
| South Korea-Switzerland | -12827.453 | 672.58 | -19.072 | *<.001* | <.001 |
| South Korea-Croatia | 15789.944 | 571.354 | 27.636 | *<.001* | <.001 |
| Colombia-Peru | -1595.522 | 515.326 | -3.096 | *0.002* | 0.637 |
| Colombia-Mexico | -3075.191 | 542.596 | -5.668 | *<.001* | <.001 |
| Colombia-Israel | -6574.556 | 615.201 | -10.687 | *<.001* | <.001 |
| Colombia-Belgium | 6735.099 | 780.667 | 8.627 | *<.001* | <.001 |
| Colombia-Chile | -6831.906 | 631.083 | -10.826 | *<.001* | <.001 |
| Colombia-Portugal | -6899.536 | 532.785 | -12.95 | *<.001* | <.001 |
| Colombia-Germany | -7000.515 | 487.64 | -14.356 | *<.001* | <.001 |
| Colombia-France | -7907.194 | 569.798 | -13.877 | *<.001* | <.001 |
| Colombia-Australia | 7989.253 | 780.667 | 10.234 | *<.001* | <.001 |
| Colombia-United States of America | -8530.216 | 523.768 | -16.286 | *<.001* | <.001 |
| Colombia-Spain | -8530.726 | 526.49 | -16.203 | *<.001* | <.001 |
| Colombia-United Kingdom | -9593.019 | 602.453 | -15.923 | *<.001* | <.001 |
| Colombia-Poland | -9821.524 | 424.549 | -23.134 | *<.001* | <.001 |
| Colombia-North Macedonia | -9823.336 | 618.432 | -15.884 | *<.001* | <.001 |
| Colombia-South Africa | -10228.923 | 560.817 | -18.239 | *<.001* | <.001 |
| Colombia-Italy | -10551.067 | 529.025 | -19.944 | *<.001* | <.001 |
| Colombia-Ireland | -10597.272 | 577.241 | -18.359 | *<.001* | <.001 |
| Colombia-Hungary | -10976.677 | 419.885 | -26.142 | *<.001* | <.001 |
| Colombia-Austria | 11156.337 | 734.648 | 15.186 | *<.001* | <.001 |
| Colombia-New Zealand | -11417.752 | 506.51 | -22.542 | *<.001* | <.001 |
| Colombia-Canada | 11646.884 | 516.559 | 22.547 | *<.001* | <.001 |
| Colombia-Switzerland | -11963.098 | 633.489 | -18.884 | *<.001* | <.001 |
| Colombia-Croatia | -14925.588 | 524.776 | -28.442 | *<.001* | <.001 |
| Peru-Mexico | 1479.668 | 510.91 | 2.896 | *0.004* | 1.000 |
| Peru-Israel | 4979.034 | 587.445 | 8.476 | *<.001* | <.001 |
| Peru-Belgium | 5139.577 | 758.986 | 6.772 | *<.001* | <.001 |
| Peru-Chile | -5236.383 | 604.056 | -8.669 | *<.001* | <.001 |
| Peru-Portugal | -5304.014 | 500.478 | -10.598 | *<.001* | <.001 |
| Peru-Germany | 5404.993 | 452.119 | 11.955 | *<.001* | <.001 |
| Peru-France | 6311.672 | 539.711 | 11.695 | *<.001* | <.001 |
| Peru-Australia | 6393.731 | 758.986 | 8.424 | *<.001* | <.001 |
| Peru-United States of America | -6934.694 | 490.868 | -14.127 | *<.001* | <.001 |
| Peru-Spain | -6935.203 | 493.772 | -14.045 | *<.001* | <.001 |
| Peru-United Kingdom | -7997.497 | 574.081 | -13.931 | *<.001* | <.001 |
| Peru-Poland | -8226.002 | 383.223 | -21.465 | *<.001* | <.001 |
| Peru-North Macedonia | 8227.813 | 590.828 | 13.926 | *<.001* | <.001 |
| Peru-South Africa | -8633.401 | 530.221 | -16.283 | *<.001* | <.001 |
| Peru-Italy | 8955.545 | 496.473 | 18.038 | *<.001* | <.001 |
| Peru-Ireland | 9001.75 | 547.563 | 16.44 | *<.001* | <.001 |
| Peru-Hungary | 9381.155 | 378.05 | 24.815 | *<.001* | <.001 |
| Peru-Austria | 9560.814 | 711.567 | 13.436 | *<.001* | <.001 |
| Peru-New Zealand | 9822.229 | 472.41 | 20.792 | *<.001* | <.001 |
| Peru-Canada | 10051.362 | 483.168 | 20.803 | *<.001* | <.001 |
| Peru-Switzerland | -10367.575 | 606.57 | -17.092 | *<.001* | <.001 |
| Peru-Croatia | 13330.066 | 491.943 | 27.097 | *<.001* | <.001 |
| Mexico-Israel | 3499.365 | 611.507 | 5.723 | *<.001* | <.001 |
| Mexico-Belgium | 3659.909 | 777.759 | 4.706 | *<.001* | 0.001 |
| Mexico-Chile | -3756.715 | 627.482 | -5.987 | *<.001* | <.001 |
| Mexico-Portugal | -3824.345 | 528.515 | -7.236 | *<.001* | <.001 |
| Mexico-Germany | 3925.324 | 482.971 | 8.127 | *<.001* | <.001 |
| Mexico-France | 4832.003 | 565.807 | 8.54 | *<.001* | <.001 |
| Mexico-Australia | 4914.062 | 777.759 | 6.318 | *<.001* | <.001 |
| Mexico-United States of America | -5455.026 | 519.423 | -10.502 | *<.001* | <.001 |
| Mexico-Spain | -5455.535 | 522.169 | -10.448 | *<.001* | <.001 |
| Mexico-United Kingdom | -6517.829 | 598.68 | -10.887 | *<.001* | <.001 |
| Mexico-Poland | -6746.334 | 419.177 | -16.094 | *<.001* | <.001 |
| Mexico-North Macedonia | -6748.145 | 614.758 | -10.977 | *<.001* | <.001 |
| Mexico-South Africa | -7153.733 | 556.762 | -12.849 | *<.001* | <.001 |
| Mexico-Italy | 7475.877 | 524.724 | 14.247 | *<.001* | <.001 |
| Mexico-Ireland | 7522.082 | 573.302 | 13.121 | *<.001* | <.001 |
| Mexico-Hungary | 7901.486 | 414.453 | 19.065 | *<.001* | <.001 |
| Mexico-Austria | 8081.146 | 731.558 | 11.046 | *<.001* | <.001 |
| Mexico-New Zealand | -8342.561 | 502.016 | -16.618 | *<.001* | <.001 |
| Mexico-Canada | 8571.694 | 512.154 | 16.737 | *<.001* | <.001 |
| Mexico-Switzerland | -8887.907 | 629.902 | -14.11 | *<.001* | <.001 |
| Mexico-Croatia | 11850.398 | 520.44 | 22.77 | *<.001* | <.001 |
| Israel-Belgium | 160.543 | 830.043 | 0.193 | *0.847* | 1.000 |
| Israel-Chile | -257.35 | 691.228 | -0.372 | *0.71* | 1.000 |
| Israel-Portugal | -324.98 | 602.819 | -0.539 | *0.59* | 1.000 |
| Israel-Germany | 425.959 | 563.314 | 0.756 | *0.45* | 1.000 |
| Israel-France | 1332.638 | 635.767 | 2.096 | *0.036* | 1.000 |
| Israel-Australia | 1414.697 | 830.043 | 1.704 | *0.088* | 1.000 |
| Israel-United States of America | -1955.66 | 594.864 | -3.288 | *0.001* | 0.328 |
| Israel-Spain | -1956.17 | 597.262 | -3.275 | *0.001* | 0.343 |
| Israel-United Kingdom | -3018.463 | 665.192 | -4.538 | *<.001* | 0.002 |
| Israel-Poland | -3246.968 | 509.678 | -6.371 | *<.001* | <.001 |
| Israel-North Macedonia | -3248.779 | 679.698 | -4.78 | *<.001* | 0.001 |
| Israel-South Africa | -3654.367 | 627.731 | -5.822 | *<.001* | <.001 |
| Israel-Italy | -3976.511 | 599.498 | -6.633 | *<.001* | <.001 |
| Israel-Ireland | 4022.716 | 642.446 | 6.262 | *<.001* | <.001 |
| Israel-Hungary | 4402.121 | 505.8 | 8.703 | *<.001* | <.001 |
| Israel-Austria | 4581.78 | 786.917 | 5.822 | *<.001* | <.001 |
| Israel-New Zealand | -4843.196 | 579.726 | -8.354 | *<.001* | <.001 |
| Israel-Canada | 5072.328 | 588.527 | 8.619 | *<.001* | <.001 |
| Israel-Switzerland | -5388.542 | 693.425 | -7.771 | *<.001* | <.001 |
| Israel-Croatia | 8351.032 | 595.752 | 14.018 | *<.001* | <.001 |
| Belgium-Chile | -96.806 | 841.882 | -0.115 | *0.908* | 1.000 |
| Belgium-Portugal | -164.437 | 770.947 | -0.213 | *0.831* | 1.000 |
| Belgium-Germany | -265.416 | 740.467 | -0.358 | *0.72* | 1.000 |
| Belgium-France | -1172.095 | 796.975 | -1.471 | *0.141* | 1.000 |
| Belgium-Australia | 1254.154 | 959.135 | 1.308 | *0.191* | 1.000 |
| Belgium-United States of America | -1795.117 | 764.743 | -2.347 | *0.019* | 1.000 |
| Belgium-Spain | -1795.626 | 766.61 | -2.342 | *0.019* | 1.000 |
| Belgium-United Kingdom | -2857.92 | 820.64 | -3.483 | *<.001* | 0.161 |
| Belgium-Poland | -3086.425 | 700.529 | -4.406 | *<.001* | 0.003 |
| Belgium-North Macedonia | -3088.236 | 832.441 | -3.71 | *<.001* | 0.067 |
| Belgium-South Africa | -3493.824 | 790.579 | -4.419 | *<.001* | 0.003 |
| Belgium-Italy | -3815.968 | 768.353 | -4.966 | *<.001* | <.001 |
| Belgium-Ireland | -3862.173 | 802.313 | -4.814 | *<.001* | <.001 |
| Belgium-Hungary | -4241.578 | 697.712 | -6.079 | *<.001* | <.001 |
| Belgium-Austria | 4421.237 | 922.067 | 4.795 | *<.001* | 0.001 |
| Belgium-New Zealand | -4682.652 | 753.028 | -6.218 | *<.001* | <.001 |
| Belgium-Canada | -4911.785 | 759.824 | -6.464 | *<.001* | <.001 |
| Belgium-Switzerland | -5227.998 | 843.687 | -6.197 | *<.001* | <.001 |
| Belgium-Croatia | -8190.489 | 765.434 | -10.7 | *<.001* | <.001 |
| Chile-Portugal | 67.63 | 619.018 | 0.109 | *0.913* | 1.000 |
| Chile-Germany | 168.609 | 580.617 | 0.29 | *0.772* | 1.000 |
| Chile-France | 1075.288 | 651.147 | 1.651 | *0.099* | 1.000 |
| Chile-Australia | 1157.347 | 841.882 | 1.375 | *0.169* | 1.000 |
| Chile-United States of America | 1698.311 | 611.274 | 2.778 | *0.005* | 1.000 |
| Chile-Spain | 1698.82 | 613.608 | 2.769 | *0.006* | 1.000 |
| Chile-United Kingdom | 2761.114 | 679.907 | 4.061 | *<.001* | 0.016 |
| Chile-Poland | 2989.619 | 528.739 | 5.654 | *<.001* | <.001 |
| Chile-North Macedonia | 2991.43 | 694.105 | 4.31 | *<.001* | 0.005 |
| Chile-South Africa | 3397.018 | 643.303 | 5.281 | *<.001* | <.001 |
| Chile-Italy | 3719.162 | 615.784 | 6.04 | *<.001* | <.001 |
| Chile-Ireland | 3765.367 | 657.67 | 5.725 | *<.001* | <.001 |
| Chile-Hungary | 4144.771 | 525.001 | 7.895 | *<.001* | <.001 |
| Chile-Austria | 4324.431 | 799.395 | 5.41 | *<.001* | <.001 |
| Chile-New Zealand | 4585.846 | 596.553 | 7.687 | *<.001* | <.001 |
| Chile-Canada | 4814.979 | 605.109 | 7.957 | *<.001* | <.001 |
| Chile-Switzerland | 5131.192 | 707.553 | 7.252 | *<.001* | <.001 |
| Chile-Croatia | 8093.683 | 612.138 | 13.222 | *<.001* | <.001 |
| Portugal-Germany | 100.979 | 471.922 | 0.214 | *0.831* | 1.000 |
| Portugal-France | 1007.658 | 556.405 | 1.811 | *0.07* | 1.000 |
| Portugal-Australia | 1089.717 | 770.947 | 1.413 | *0.158* | 1.000 |
| Portugal-United States of America | -1630.681 | 509.166 | -3.203 | *0.001* | 0.443 |
| Portugal-Spain | -1631.19 | 511.967 | -3.186 | *0.001* | 0.469 |
| Portugal-United Kingdom | -2693.483 | 589.803 | -4.567 | *<.001* | 0.002 |
| Portugal-Poland | 2921.988 | 406.398 | 7.19 | *<.001* | <.001 |
| Portugal-North Macedonia | 2923.8 | 606.116 | 4.824 | *<.001* | <.001 |
| Portugal-South Africa | -3329.388 | 547.205 | -6.084 | *<.001* | <.001 |
| Portugal-Italy | 3651.531 | 514.572 | 7.096 | *<.001* | <.001 |
| Portugal-Ireland | 3697.737 | 564.025 | 6.556 | *<.001* | <.001 |
| Portugal-Hungary | 4077.141 | 401.523 | 10.154 | *<.001* | <.001 |
| Portugal-Austria | 4256.801 | 724.311 | 5.877 | *<.001* | <.001 |
| Portugal-New Zealand | 4518.216 | 491.396 | 9.195 | *<.001* | <.001 |
| Portugal-Canada | 4747.349 | 501.748 | 9.462 | *<.001* | <.001 |
| Portugal-Switzerland | -5063.562 | 621.471 | -8.148 | *<.001* | <.001 |
| Portugal-Croatia | 8026.053 | 510.203 | 15.731 | *<.001* | <.001 |
| Germany-France | 906.679 | 513.342 | 1.766 | *0.077* | 1.000 |
| Germany-Australia | 988.738 | 740.467 | 1.335 | *0.182* | 1.000 |
| Germany-United States of America | -1529.701 | 461.717 | -3.313 | *<.001* | 0.300 |
| Germany-Spain | -1530.211 | 464.804 | -3.292 | *<.001* | 0.323 |
| Germany-United Kingdom | -2592.504 | 549.364 | -4.719 | *<.001* | 0.001 |
| Germany-Poland | -2821.009 | 345.097 | -8.175 | *<.001* | <.001 |
| Germany-North Macedonia | -2822.82 | 566.841 | -4.98 | *<.001* | <.001 |
| Germany-South Africa | -3228.408 | 503.355 | -6.414 | *<.001* | <.001 |
| Germany-Italy | -3550.552 | 467.672 | -7.592 | *<.001* | <.001 |
| Germany-Ireland | -3596.757 | 521.591 | -6.896 | *<.001* | <.001 |
| Germany-Hungary | -3976.162 | 339.343 | -11.717 | *<.001* | <.001 |
| Germany-Austria | 4155.821 | 691.779 | 6.007 | *<.001* | <.001 |
| Germany-New Zealand | -4417.237 | 442.044 | -9.993 | *<.001* | <.001 |
| Germany-Canada | 4646.369 | 453.524 | 10.245 | *<.001* | <.001 |
| Germany-Switzerland | -4962.583 | 583.231 | -8.509 | *<.001* | <.001 |
| Germany-Croatia | 7925.073 | 462.861 | 17.122 | *<.001* | <.001 |
| France-Australia | 82.059 | 796.975 | 0.103 | *0.918* | 1.000 |
| France-United States of America | -623.022 | 547.777 | -1.137 | *0.255* | 1.000 |
| France-Spain | -623.532 | 550.381 | -1.133 | *0.257* | 1.000 |
| France-United Kingdom | -1685.825 | 623.44 | -2.704 | *0.007* | 1.000 |
| France-Poland | -1914.33 | 453.838 | -4.218 | *<.001* | 0.008 |
| France-North Macedonia | -1916.141 | 638.894 | -2.999 | *0.003* | 0.880 |
| France-South Africa | -2321.729 | 583.304 | -3.98 | *<.001* | 0.022 |
| France-Italy | -2643.873 | 552.806 | -4.783 | *<.001* | 0.001 |
| France-Ireland | -2690.078 | 599.111 | -4.49 | *<.001* | 0.002 |
| France-Hungary | -3069.483 | 449.478 | -6.829 | *<.001* | <.001 |
| France-Austria | 3249.142 | 751.954 | 4.321 | *<.001* | 0.005 |
| France-New Zealand | -3510.558 | 531.3 | -6.607 | *<.001* | <.001 |
| France-Canada | 3739.69 | 540.888 | 6.914 | *<.001* | <.001 |
| France-Switzerland | -4055.903 | 653.479 | -6.207 | *<.001* | <.001 |
| France-Croatia | 7018.394 | 548.741 | 12.79 | *<.001* | <.001 |
| Australia-United States of America | -540.963 | 764.743 | -0.707 | *0.479* | 1.000 |
| Australia-Spain | -541.473 | 766.61 | -0.706 | *0.48* | 1.000 |
| Australia-United Kingdom | -1603.766 | 820.64 | -1.954 | *0.051* | 1.000 |
| Australia-Poland | -1832.271 | 700.529 | -2.616 | *0.009* | 1.000 |
| Australia-North Macedonia | -1834.082 | 832.441 | -2.203 | *0.028* | 1.000 |
| Australia-South Africa | -2239.67 | 790.579 | -2.833 | *0.005* | 1.000 |
| Australia-Italy | -2561.814 | 768.353 | -3.334 | *<.001* | 0.278 |
| Australia-Ireland | -2608.019 | 802.313 | -3.251 | *0.001* | 0.374 |
| Australia-Hungary | -2987.424 | 697.712 | -4.282 | *<.001* | 0.006 |
| Australia-Austria | -3167.083 | 922.067 | -3.435 | *<.001* | 0.193 |
| Australia-New Zealand | -3428.499 | 753.028 | -4.553 | *<.001* | 0.002 |
| Australia-Canada | -3657.631 | 759.824 | -4.814 | *<.001* | <.001 |
| Australia-Switzerland | -3973.845 | 843.687 | -4.71 | *<.001* | 0.001 |
| Australia-Croatia | -6936.335 | 765.434 | -9.062 | *<.001* | <.001 |
| United States of America-Spain | 0.509 | 502.576 | 0.001 | *0.999* | 1.000 |
| United States of America-United Kingdom | 1062.803 | 581.67 | 1.827 | *0.068* | 1.000 |
| United States of America-Poland | 1291.308 | 394.502 | 3.273 | *0.001* | 0.346 |
| United States of America-North Macedonia | 1293.119 | 598.205 | 2.162 | *0.031* | 1.000 |
| United States of America-South Africa | 1698.707 | 538.429 | 3.155 | *0.002* | 0.522 |
| United States of America-Italy | 2020.851 | 505.23 | 4 | *<.001* | 0.021 |
| United States of America-Ireland | 2067.056 | 555.515 | 3.721 | *<.001* | 0.064 |
| United States of America-Hungary | 2446.46 | 389.478 | 6.281 | *<.001* | <.001 |
| United States of America-Austria | 2626.12 | 717.704 | 3.659 | *<.001* | 0.082 |
| United States of America-New Zealand | 2887.535 | 481.604 | 5.996 | *<.001* | <.001 |
| United States of America-Canada | 3116.668 | 492.162 | 6.333 | *<.001* | <.001 |
| United States of America-Switzerland | 3432.881 | 613.758 | 5.593 | *<.001* | <.001 |
| United States of America-Croatia | 6395.372 | 500.779 | 12.771 | *<.001* | <.001 |
| Spain-United Kingdom | -1062.294 | 584.123 | -1.819 | *0.069* | 1.000 |
| Spain-Poland | 1290.799 | 398.11 | 3.242 | *0.001* | 0.385 |
| Spain-North Macedonia | 1292.61 | 600.59 | 2.152 | *0.031* | 1.000 |
| Spain-South Africa | 1698.198 | 541.078 | 3.139 | *0.002* | 0.552 |
| Spain-Italy | 2020.342 | 508.052 | 3.977 | *<.001* | 0.023 |
| Spain-Ireland | 2066.547 | 558.083 | 3.703 | *<.001* | 0.069 |
| Spain-Hungary | 2445.951 | 393.132 | 6.222 | *<.001* | <.001 |
| Spain-Austria | 2625.611 | 719.693 | 3.648 | *<.001* | 0.086 |
| Spain-New Zealand | 2887.026 | 484.564 | 5.958 | *<.001* | <.001 |
| Spain-Canada | 3116.159 | 495.058 | 6.295 | *<.001* | <.001 |
| Spain-Switzerland | -3432.372 | 616.083 | -5.571 | *<.001* | <.001 |
| Spain-Croatia | 6394.863 | 503.626 | 12.698 | *<.001* | <.001 |
| United Kingdom-Poland | 228.505 | 494.216 | 0.462 | *0.644* | 1.000 |
| United Kingdom-North Macedonia | 230.316 | 668.182 | 0.345 | *0.73* | 1.000 |
| United Kingdom-South Africa | 635.904 | 615.243 | 1.034 | *0.301* | 1.000 |
| United Kingdom-Italy | 958.048 | 586.409 | 1.634 | *0.102* | 1.000 |
| United Kingdom-Ireland | 1004.253 | 630.25 | 1.593 | *0.111* | 1.000 |
| United Kingdom-Hungary | 1383.658 | 490.215 | 2.823 | *0.005* | 1.000 |
| United Kingdom-Austria | 1563.317 | 776.992 | 2.012 | *0.044* | 1.000 |
| United Kingdom-New Zealand | 1824.732 | 566.18 | 3.223 | *0.001* | 0.412 |
| United Kingdom-Canada | 2053.865 | 575.188 | 3.571 | *<.001* | 0.116 |
| United Kingdom-Switzerland | 2370.078 | 682.141 | 3.474 | *<.001* | 0.166 |
| United Kingdom-Croatia | 5332.569 | 582.578 | 9.153 | *<.001* | <.001 |
| Poland-North Macedonia | 1.811 | 513.574 | 0.004 | *0.997* | 1.000 |
| Poland-South Africa | -407.399 | 442.51 | -0.921 | *0.357* | 1.000 |
| Poland-Italy | 729.543 | 401.455 | 1.817 | *0.069* | 1.000 |
| Poland-Ireland | 775.748 | 463.148 | 1.675 | *0.094* | 1.000 |
| Poland-Hungary | 1155.153 | 240.005 | 4.813 | *<.001* | <.001 |
| Poland-Austria | 1334.812 | 648.851 | 2.057 | *0.04* | 1.000 |
| Poland-New Zealand | 1596.227 | 371.284 | 4.299 | *<.001* | 0.006 |
| Poland-Canada | 1825.36 | 384.88 | 4.743 | *<.001* | 0.001 |
| Poland-Switzerland | -2141.573 | 531.608 | -4.028 | *<.001* | 0.018 |
| Poland-Croatia | 5104.064 | 395.84 | 12.894 | *<.001* | <.001 |
| North Macedonia-South Africa | -405.588 | 630.898 | -0.643 | *0.52* | 1.000 |
| North Macedonia-Italy | 727.732 | 602.813 | 1.207 | *0.227* | 1.000 |
| North Macedonia-Ireland | 773.937 | 645.541 | 1.199 | *0.231* | 1.000 |
| North Macedonia-Hungary | 1153.341 | 509.725 | 2.263 | *0.024* | 1.000 |
| North Macedonia-Austria | 1333.001 | 789.446 | 1.689 | *0.091* | 1.000 |
| North Macedonia-New Zealand | 1594.416 | 583.154 | 2.734 | *0.006* | 1.000 |
| North Macedonia-Canada | 1823.549 | 591.903 | 3.081 | *0.002* | 0.671 |
| North Macedonia-Switzerland | -2139.762 | 696.293 | -3.073 | *0.002* | 0.689 |
| North Macedonia-Croatia | 5102.253 | 599.088 | 8.517 | *<.001* | <.001 |
| South Africa-Italy | 322.144 | 543.545 | 0.593 | *0.553* | 1.000 |
| South Africa-Ireland | 368.349 | 590.576 | 0.624 | *0.533* | 1.000 |
| South Africa-Hungary | 747.753 | 438.037 | 1.707 | *0.088* | 1.000 |
| South Africa-Austria | 927.413 | 745.173 | 1.245 | *0.213* | 1.000 |
| South Africa-New Zealand | 1188.828 | 521.657 | 2.279 | *0.023* | 1.000 |
| South Africa-Canada | 1417.961 | 531.42 | 2.668 | *0.008* | 1.000 |
| South Africa-Switzerland | -1734.174 | 645.664 | -2.686 | *0.007* | 1.000 |
| South Africa-Croatia | 4696.665 | 539.41 | 8.707 | *<.001* | <.001 |
| Italy-Ireland | 46.205 | 560.474 | 0.082 | *0.934* | 1.000 |
| Italy-Hungary | 425.61 | 396.52 | 1.073 | *0.283* | 1.000 |
| Italy-Austria | 605.269 | 721.549 | 0.839 | *0.402* | 1.000 |
| Italy-New Zealand | -866.684 | 487.316 | -1.778 | *0.075* | 1.000 |
| Italy-Canada | 1095.817 | 497.753 | 2.202 | *0.028* | 1.000 |
| Italy-Switzerland | -1412.03 | 618.25 | -2.284 | *0.022* | 1.000 |
| Italy-Croatia | 4374.521 | 506.275 | 8.641 | *<.001* | <.001 |
| Ireland-Hungary | 379.404 | 458.876 | 0.827 | *0.408* | 1.000 |
| Ireland-Austria | 559.064 | 757.61 | 0.738 | *0.461* | 1.000 |
| Ireland-New Zealand | -820.479 | 539.274 | -1.521 | *0.128* | 1.000 |
| Ireland-Canada | 1049.612 | 548.724 | 1.913 | *0.056* | 1.000 |
| Ireland-Switzerland | -1365.825 | 659.979 | -2.069 | *0.038* | 1.000 |
| Ireland-Croatia | 4328.316 | 556.466 | 7.778 | *<.001* | <.001 |
| Hungary-Austria | 179.66 | 645.809 | 0.278 | *0.781* | 1.000 |
| Hungary-New Zealand | -441.075 | 365.941 | -1.205 | *0.228* | 1.000 |
| Hungary-Canada | 670.208 | 379.729 | 1.765 | *0.078* | 1.000 |
| Hungary-Switzerland | -986.421 | 527.891 | -1.869 | *0.062* | 1.000 |
| Hungary-Croatia | 3948.912 | 390.833 | 10.104 | *<.001* | <.001 |
| Austria-New Zealand | -261.415 | 705.208 | -0.371 | *0.711* | 1.000 |
| Austria-Canada | -490.548 | 712.46 | -0.689 | *0.491* | 1.000 |
| Austria-Switzerland | -806.761 | 801.295 | -1.007 | *0.314* | 1.000 |
| Austria-Croatia | -3769.252 | 718.44 | -5.246 | *<.001* | <.001 |
| New Zealand-Canada | 229.133 | 473.754 | 0.484 | *0.629* | 1.000 |
| New Zealand-Switzerland | -545.346 | 599.098 | -0.91 | *0.363* | 1.000 |
| New Zealand-Croatia | 3507.837 | 482.7 | 7.267 | *<.001* | <.001 |
| Canada-Switzerland | -316.213 | 607.618 | -0.52 | *0.603* | 1.000 |
| Canada-Croatia | -3278.704 | 493.235 | -6.647 | *<.001* | <.001 |
| Switzerland-Croatia | 2962.491 | 614.618 | 4.82 | *<.001* | <.001 |

Note. a = Significance values have been adjusted by the Bonferroni correction for multiple tests.

Table L. Country-based Pairwise Comparisons in the Kruskal-Wallis Test on the Sexual Abuse History Questionnaire – Child Subscale (SAHQ)

| **Country comparisons** | **Test statistic** | **Standard error** | **Standardized test statistic**  **(z)** | ***p*-value** | **Adjusted *p*-value ^a^** |
| --- | --- | --- | --- | --- | --- |
| North Macedonia-Spain | -467.497 | 524.251 | -0.892 | *0.373* | 1.000 |
| North Macedonia-Hungary | 604.091 | 446.554 | 1.353 | *0.176* | 1.000 |
| North Macedonia-Belgium | 1616.031 | 721.771 | 2.239 | *0.025* | 1.000 |
| North Macedonia-Croatia | 1669.779 | 522.223 | 3.197 | *0.001* | 0.451 |
| North Macedonia-Germany | 1791.649 | 497.107 | 3.604 | *<.001* | 0.102 |
| North Macedonia-Italy | 2133.551 | 522.185 | 4.086 | *<.001* | 0.014 |
| North Macedonia-Austria | 2148.757 | 689.663 | 3.116 | *0.002* | 0.596 |
| North Macedonia-Switzerland | -2380.494 | 609.755 | -3.904 | *<.001* | 0.031 |
| North Macedonia-Poland | -2725.315 | 449.480 | -6.063 | *<.001* | <.001 |
| North Macedonia-Malaysia | 2762.315 | 611.734 | 4.516 | *<.001* | 0.002 |
| North Macedonia-Chile | -3348.551 | 606.594 | -5.520 | *<.001* | <.001 |
| North Macedonia-France | 3573.806 | 554.386 | 6.446 | *<.001* | <.001 |
| North Macedonia-South Africa | -3613.131 | 548.187 | -6.591 | *<.001* | <.001 |
| North Macedonia-Portugal | -3718.184 | 526.574 | -7.061 | *<.001* | <.001 |
| North Macedonia-Ireland | 3781.768 | 559.026 | 6.765 | *<.001* | <.001 |
| North Macedonia-Israel | 3920.018 | 588.954 | 6.656 | *<.001* | <.001 |
| North Macedonia-United Kingdom | -4141.743 | 581.729 | -7.120 | *<.001* | <.001 |
| North Macedonia-Canada | 4182.449 | 516.540 | 8.097 | *<.001* | <.001 |
| North Macedonia-South Korea | -4393.232 | 579.365 | -7.583 | *<.001* | <.001 |
| North Macedonia-Colombia | 4600.661 | 546.730 | 8.415 | *<.001* | <.001 |
| North Macedonia-New Zealand | 5012.373 | 509.102 | 9.846 | *<.001* | <.001 |
| North Macedonia-Australia | 5116.448 | 725.937 | 7.048 | *<.001* | <.001 |
| North Macedonia-United States of America | -5732.420 | 521.540 | -10.991 | *<.001* | <.001 |
| North Macedonia-Peru | -7548.065 | 513.527 | -14.698 | *<.001* | <.001 |
| North Macedonia-Mexico | 9842.493 | 534.198 | 18.425 | *<.001* | <.001 |
| Spain-Hungary | 136.594 | 338.294 | 0.404 | *0.686* | 1.000 |
| Spain-Belgium | 1148.534 | 660.292 | 1.739 | *0.082* | 1.000 |
| Spain-Croatia | 1202.282 | 433.301 | 2.775 | *0.006* | 1.000 |
| Spain-Germany | 1324.152 | 402.676 | 3.288 | *0.001* | 0.327 |
| Spain-Italy | 1666.054 | 433.255 | 3.845 | *<.001* | 0.039 |
| Spain-Austria | 1681.260 | 625.034 | 2.690 | *0.007* | 1.000 |
| Spain-Switzerland | -1912.997 | 535.568 | -3.572 | *<.001* | 0.115 |
| Spain-Poland | 2257.818 | 342.147 | 6.599 | *<.001* | <.001 |
| Spain-Malaysia | 2294.818 | 537.821 | 4.267 | *<.001* | 0.006 |
| Spain-Chile | -2881.054 | 531.966 | -5.416 | *<.001* | <.001 |
| Spain-France | 3106.308 | 471.568 | 6.587 | *<.001* | <.001 |
| Spain-South Africa | 3145.634 | 464.265 | 6.776 | *<.001* | <.001 |
| Spain-Portugal | 3250.687 | 438.535 | 7.413 | *<.001* | <.001 |
| Spain-Ireland | 3314.271 | 477.015 | 6.948 | *<.001* | <.001 |
| Spain-Israel | 3452.521 | 511.761 | 6.746 | *<.001* | <.001 |
| Spain-United Kingdom | -3674.246 | 503.429 | -7.298 | *<.001* | <.001 |
| Spain-Canada | 3714.952 | 426.434 | 8.712 | *<.001* | <.001 |
| Spain-South Korea | 3925.735 | 500.696 | 7.841 | *<.001* | <.001 |
| Spain-Colombia | 4133.164 | 462.543 | 8.936 | *<.001* | <.001 |
| Spain-New Zealand | 4544.876 | 417.394 | 10.889 | *<.001* | <.001 |
| Spain-Australia | 4648.951 | 664.843 | 6.993 | *<.001* | <.001 |
| Spain-United States of America | -5264.923 | 432.477 | -12.174 | *<.001* | <.001 |
| Spain-Peru | 7080.568 | 422.779 | 16.748 | *<.001* | <.001 |
| Spain-Mexico | 9374.996 | 447.660 | 20.942 | *<.001* | <.001 |
| Hungary-Belgium | 1011.940 | 600.463 | 1.685 | *0.092* | 1.000 |
| Hungary-Croatia | 1065.688 | 335.143 | 3.180 | *0.001* | 0.479 |
| Hungary-Germany | 1187.558 | 294.481 | 4.033 | *<.001* | 0.018 |
| Hungary-Italy | -1529.460 | 335.083 | -4.564 | *<.001* | 0.002 |
| Hungary-Austria | 1544.666 | 561.461 | 2.751 | *0.006* | 1.000 |
| Hungary-Switzerland | -1776.403 | 459.787 | -3.864 | *<.001* | 0.036 |
| Hungary-Poland | -2121.224 | 204.047 | -10.396 | *<.001* | <.001 |
| Hungary-Malaysia | -2158.224 | 462.409 | -4.667 | *<.001* | 0.001 |
| Hungary-Chile | -2744.459 | 455.587 | -6.024 | *<.001* | <.001 |
| Hungary-France | 2969.714 | 383.337 | 7.747 | *<.001* | <.001 |
| Hungary-South Africa | -3009.040 | 374.316 | -8.039 | *<.001* | <.001 |
| Hungary-Portugal | -3114.093 | 341.883 | -9.109 | *<.001* | <.001 |
| Hungary-Ireland | -3177.677 | 390.018 | -8.148 | *<.001* | <.001 |
| Hungary-Israel | -3315.927 | 431.823 | -7.679 | *<.001* | <.001 |
| Hungary-United Kingdom | -3537.652 | 421.915 | -8.385 | *<.001* | <.001 |
| Hungary-Canada | 3578.358 | 326.216 | 10.969 | *<.001* | <.001 |
| Hungary-South Korea | -3789.141 | 418.650 | -9.051 | *<.001* | <.001 |
| Hungary-Colombia | 3996.569 | 372.179 | 10.738 | *<.001* | <.001 |
| Hungary-New Zealand | -4408.282 | 314.307 | -14.025 | *<.001* | <.001 |
| Hungary-Australia | 4512.356 | 605.465 | 7.453 | *<.001* | <.001 |
| Hungary-United States of America | -5128.329 | 334.078 | -15.351 | *<.001* | <.001 |
| Hungary-Peru | -6943.973 | 321.423 | -21.604 | *<.001* | <.001 |
| Hungary-Mexico | -9238.402 | 353.512 | -26.133 | *<.001* | <.001 |
| Belgium-Croatia | -53.748 | 658.683 | -0.082 | *0.935* | 1.000 |
| Belgium-Germany | -175.618 | 638.953 | -0.275 | *0.783* | 1.000 |
| Belgium-Italy | -517.520 | 658.652 | -0.786 | *0.432* | 1.000 |
| Belgium-Austria | 532.726 | 797.986 | 0.668 | *0.504* | 1.000 |
| Belgium-Switzerland | -764.463 | 730.032 | -1.047 | *0.295* | 1.000 |
| Belgium-Poland | -1109.284 | 602.642 | -1.841 | *0.066* | 1.000 |
| Belgium-Malaysia | -1146.284 | 731.686 | -1.567 | *0.117* | 1.000 |
| Belgium-Chile | -1732.520 | 727.394 | -2.382 | *0.017* | 1.000 |
| Belgium-France | -1957.774 | 684.463 | -2.860 | *0.004* | 1.000 |
| Belgium-South Africa | -1997.100 | 679.452 | -2.939 | *0.003* | 1.000 |
| Belgium-Portugal | -2102.153 | 662.137 | -3.175 | *0.001* | 0.487 |
| Belgium-Ireland | -2165.737 | 688.227 | -3.147 | *0.002* | 0.536 |
| Belgium-Israel | -2303.987 | 712.750 | -3.233 | *0.001* | 0.399 |
| Belgium-United Kingdom | -2525.712 | 706.792 | -3.573 | *<.001* | 0.114 |
| Belgium-Canada | -2566.418 | 654.186 | -3.923 | *<.001* | 0.028 |
| Belgium-South Korea | -2777.201 | 704.847 | -3.940 | *<.001* | 0.026 |
| Belgium-Colombia | -2984.630 | 678.277 | -4.400 | *<.001* | 0.004 |
| Belgium-New Zealand | -3396.342 | 648.329 | -5.239 | *<.001* | <.001 |
| Belgium-Australia | 3500.416 | 829.536 | 4.220 | *<.001* | 0.008 |
| Belgium-United States of America | -4116.389 | 658.141 | -6.255 | *<.001* | <.001 |
| Belgium-Peru | -5932.033 | 651.809 | -9.101 | *<.001* | <.001 |
| Belgium-Mexico | -8226.462 | 668.216 | -12.311 | *<.001* | <.001 |
| Croatia-Germany | -121.870 | 400.032 | -0.305 | *0.761* | 1.000 |
| Croatia-Italy | -463.772 | 430.798 | -1.077 | *0.282* | 1.000 |
| Croatia-Austria | 478.978 | 623.334 | 0.768 | *0.442* | 1.000 |
| Croatia-Switzerland | -710.715 | 533.583 | -1.332 | *0.183* | 1.000 |
| Croatia-Poland | -1055.536 | 339.031 | -3.113 | *0.002* | 0.601 |
| Croatia-Malaysia | -1092.536 | 535.844 | -2.039 | *0.041* | 1.000 |
| Croatia-Chile | -1678.772 | 529.968 | -3.168 | *0.002* | 0.499 |
| Croatia-France | -1904.027 | 469.312 | -4.057 | *<.001* | 0.016 |
| Croatia-South Africa | -1943.352 | 461.973 | -4.207 | *<.001* | 0.008 |
| Croatia-Portugal | -2048.405 | 436.108 | -4.697 | *<.001* | 0.001 |
| Croatia-Ireland | -2111.989 | 474.785 | -4.448 | *<.001* | 0.003 |
| Croatia-Israel | -2250.239 | 509.684 | -4.415 | *<.001* | 0.003 |
| Croatia-United Kingdom | -2471.964 | 501.317 | -4.931 | *<.001* | <.001 |
| Croatia-Canada | 2512.670 | 423.938 | 5.927 | *<.001* | <.001 |
| Croatia-South Korea | -2723.453 | 498.572 | -5.463 | *<.001* | <.001 |
| Croatia-Colombia | 2930.882 | 460.243 | 6.368 | *<.001* | <.001 |
| Croatia-New Zealand | -3342.594 | 414.844 | -8.057 | *<.001* | <.001 |
| Croatia-Australia | 3446.669 | 663.245 | 5.197 | *<.001* | <.001 |
| Croatia-United States of America | -4062.641 | 430.017 | -9.448 | *<.001* | <.001 |
| Croatia-Peru | -5878.286 | 420.261 | -13.987 | *<.001* | <.001 |
| Croatia-Mexico | -8172.714 | 445.283 | -18.354 | *<.001* | <.001 |
| Germany-Italy | -341.902 | 399.982 | -0.855 | *0.393* | 1.000 |
| Germany-Austria | 357.108 | 602.448 | 0.593 | *0.553* | 1.000 |
| Germany-Switzerland | -588.845 | 509.027 | -1.157 | *0.247* | 1.000 |
| Germany-Poland | -933.666 | 298.898 | -3.124 | *0.002* | 0.580 |
| Germany-Malaysia | -970.666 | 511.397 | -1.898 | *0.058* | 1.000 |
| Germany-Chile | -1556.901 | 505.237 | -3.082 | *0.002* | 0.669 |
| Germany-France | 1782.156 | 441.194 | 4.039 | *<.001* | 0.017 |
| Germany-South Africa | -1821.482 | 433.379 | -4.203 | *<.001* | 0.009 |
| Germany-Portugal | -1926.535 | 405.695 | -4.749 | *<.001* | 0.001 |
| Germany-Ireland | -1990.119 | 447.011 | -4.452 | *<.001* | 0.003 |
| Germany-Israel | -2128.369 | 483.917 | -4.398 | *<.001* | 0.004 |
| Germany-United Kingdom | -2350.094 | 475.097 | -4.947 | *<.001* | <.001 |
| Germany-Canada | 2390.800 | 392.583 | 6.090 | *<.001* | <.001 |
| Germany-South Korea | -2601.583 | 472.200 | -5.509 | *<.001* | <.001 |
| Germany-Colombia | 2809.011 | 431.534 | 6.509 | *<.001* | <.001 |
| Germany-New Zealand | -3220.724 | 382.745 | -8.415 | *<.001* | <.001 |
| Germany-Australia | 3324.798 | 643.656 | 5.165 | *<.001* | <.001 |
| Germany-United States of America | -3940.771 | 399.140 | -9.873 | *<.001* | <.001 |
| Germany-Peru | -5756.415 | 388.610 | -14.813 | *<.001* | <.001 |
| Germany-Mexico | -8050.844 | 415.542 | -19.374 | *<.001* | <.001 |
| Italy-Austria | 15.206 | 623.302 | 0.024 | *0.981* | 1.000 |
| Italy-Switzerland | -246.943 | 533.545 | -0.463 | *0.643* | 1.000 |
| Italy-Poland | -591.764 | 338.972 | -1.746 | *0.081* | 1.000 |
| Italy-Malaysia | -628.764 | 535.807 | -1.173 | *0.241* | 1.000 |
| Italy-Chile | -1215.000 | 529.930 | -2.293 | *0.022* | 1.000 |
| Italy-France | 1440.254 | 469.270 | 3.069 | *0.002* | 0.698 |
| Italy-South Africa | -1479.580 | 461.930 | -3.203 | *0.001* | 0.442 |
| Italy-Portugal | -1584.633 | 436.062 | -3.634 | *<.001* | 0.091 |
| Italy-Ireland | 1648.217 | 474.743 | 3.472 | *<.001* | 0.168 |
| Italy-Israel | 1786.467 | 509.644 | 3.505 | *<.001* | 0.148 |
| Italy-United Kingdom | -2008.192 | 501.277 | -4.006 | *<.001* | 0.020 |
| Italy-Canada | 2048.898 | 423.891 | 4.834 | *<.001* | <.001 |
| Italy-South Korea | -2259.681 | 498.532 | -4.533 | *<.001* | 0.002 |
| Italy-Colombia | 2467.110 | 460.200 | 5.361 | *<.001* | <.001 |
| Italy-New Zealand | -2878.822 | 414.796 | -6.940 | *<.001* | <.001 |
| Italy-Australia | 2982.897 | 663.215 | 4.498 | *<.001* | 0.002 |
| Italy-United States of America | -3598.869 | 429.970 | -8.370 | *<.001* | <.001 |
| Italy-Peru | -5414.514 | 420.214 | -12.885 | *<.001* | <.001 |
| Italy-Mexico | -7708.942 | 445.239 | -17.314 | *<.001* | <.001 |
| Austria-Switzerland | -231.737 | 698.304 | -0.332 | *0.740* | 1.000 |
| Austria-Poland | -576.558 | 563.791 | -1.023 | *0.306* | 1.000 |
| Austria-Malaysia | -613.558 | 700.034 | -0.876 | *0.381* | 1.000 |
| Austria-Chile | -1199.794 | 695.546 | -1.725 | *0.085* | 1.000 |
| Austria-France | -1425.048 | 650.517 | -2.191 | *0.028* | 1.000 |
| Austria-South Africa | -1464.374 | 645.242 | -2.269 | *0.023* | 1.000 |
| Austria-Portugal | -1569.427 | 626.984 | -2.503 | *0.012* | 1.000 |
| Austria-Ireland | -1633.011 | 654.476 | -2.495 | *0.013* | 1.000 |
| Austria-Israel | -1771.261 | 680.217 | -2.604 | *0.009* | 1.000 |
| Austria-United Kingdom | -1992.986 | 673.971 | -2.957 | *0.003* | 1.000 |
| Austria-Canada | -2033.692 | 618.580 | -3.288 | *0.001* | 0.328 |
| Austria-South Korea | -2244.475 | 671.932 | -3.340 | *<.001* | 0.272 |
| Austria-Colombia | -2451.904 | 644.005 | -3.807 | *<.001* | 0.046 |
| Austria-New Zealand | -2863.616 | 612.384 | -4.676 | *<.001* | 0.001 |
| Austria-Australia | 2967.691 | 801.756 | 3.701 | *<.001* | 0.070 |
| Austria-United States of America | -3583.663 | 622.762 | -5.754 | *<.001* | <.001 |
| Austria-Peru | -5399.308 | 616.066 | -8.764 | *<.001* | <.001 |
| Austria-Mexico | -7693.736 | 633.400 | -12.147 | *<.001* | <.001 |
| Switzerland-Poland | 344.821 | 462.629 | 0.745 | *0.456* | 1.000 |
| Switzerland-Malaysia | 381.820 | 621.460 | 0.614 | *0.539* | 1.000 |
| Switzerland-Chile | -968.056 | 616.400 | -1.570 | *0.116* | 1.000 |
| Switzerland-France | 1193.311 | 565.100 | 2.112 | *0.035* | 1.000 |
| Switzerland-South Africa | 1232.637 | 559.019 | 2.205 | *0.027* | 1.000 |
| Switzerland-Portugal | 1337.690 | 537.842 | 2.487 | *0.013* | 1.000 |
| Switzerland-Ireland | 1401.274 | 569.653 | 2.460 | *0.014* | 1.000 |
| Switzerland-Israel | 1539.524 | 599.050 | 2.570 | *0.010* | 1.000 |
| Switzerland-United Kingdom | -1761.249 | 591.948 | -2.975 | *0.003* | 0.951 |
| Switzerland-Canada | 1801.955 | 528.022 | 3.413 | *<.001* | 0.209 |
| Switzerland-South Korea | 2012.738 | 589.625 | 3.414 | *<.001* | 0.208 |
| Switzerland-Colombia | 2220.166 | 557.590 | 3.982 | *<.001* | 0.022 |
| Switzerland-New Zealand | 2631.879 | 520.748 | 5.054 | *<.001* | <.001 |
| Switzerland-Australia | 2735.953 | 734.151 | 3.727 | *<.001* | 0.063 |
| Switzerland-United States of America | -3351.926 | 532.914 | -6.290 | *<.001* | <.001 |
| Switzerland-Peru | 5167.570 | 525.074 | 9.842 | *<.001* | <.001 |
| Switzerland-Mexico | 7461.999 | 545.308 | 13.684 | *<.001* | <.001 |
| Poland-Malaysia | 37.000 | 465.235 | 0.080 | *0.937* | 1.000 |
| Poland-Chile | -623.235 | 458.455 | -1.359 | *0.174* | 1.000 |
| Poland-France | 848.490 | 386.741 | 2.194 | *0.028* | 1.000 |
| Poland-South Africa | -887.816 | 377.802 | -2.350 | *0.019* | 1.000 |
| Poland-Portugal | -992.869 | 345.695 | -2.872 | *0.004* | 1.000 |
| Poland-Ireland | 1056.453 | 393.364 | 2.686 | *0.007* | 1.000 |
| Poland-Israel | 1194.703 | 434.847 | 2.747 | *0.006* | 1.000 |
| Poland-United Kingdom | -1416.428 | 425.010 | -3.333 | *<.001* | 0.280 |
| Poland-Canada | 1457.134 | 330.209 | 4.413 | *<.001* | 0.003 |
| Poland-South Korea | -1667.917 | 421.769 | -3.955 | *<.001* | 0.025 |
| Poland-Colombia | 1875.345 | 375.684 | 4.992 | *<.001* | <.001 |
| Poland-New Zealand | 2287.058 | 318.450 | 7.182 | *<.001* | <.001 |
| Poland-Australia | 2391.132 | 607.626 | 3.935 | *<.001* | 0.027 |
| Poland-United States of America | -3007.105 | 337.978 | -8.897 | *<.001* | <.001 |
| Poland-Peru | 4822.749 | 325.476 | 14.818 | *<.001* | <.001 |
| Poland-Mexico | 7117.178 | 357.200 | 19.925 | *<.001* | <.001 |
| Malaysia-Chile | -586.236 | 618.359 | -0.948 | *0.343* | 1.000 |
| Malaysia-France | 811.491 | 567.235 | 1.431 | *0.153* | 1.000 |
| Malaysia-South Africa | -850.816 | 561.178 | -1.516 | *0.129* | 1.000 |
| Malaysia-Portugal | -955.869 | 540.085 | -1.770 | *0.077* | 1.000 |
| Malaysia-Ireland | 1019.453 | 571.771 | 1.783 | *0.075* | 1.000 |
| Malaysia-Israel | 1157.703 | 601.065 | 1.926 | *0.054* | 1.000 |
| Malaysia-United Kingdom | -1379.428 | 593.987 | -2.322 | *0.020* | 1.000 |
| Malaysia-Canada | 1420.134 | 530.307 | 2.678 | *0.007* | 1.000 |
| Malaysia-South Korea | -1630.917 | 591.672 | -2.756 | *0.006* | 1.000 |
| Malaysia-Colombia | 1838.346 | 559.755 | 3.284 | *0.001* | 0.332 |
| Malaysia-New Zealand | -2250.058 | 523.065 | -4.302 | *<.001* | 0.006 |
| Malaysia-Australia | 2354.133 | 735.796 | 3.199 | *0.001* | 0.448 |
| Malaysia-United States of America | -2970.105 | 535.178 | -5.550 | *<.001* | <.001 |
| Malaysia-Peru | -4785.750 | 527.372 | -9.075 | *<.001* | <.001 |
| Malaysia-Mexico | -7080.178 | 547.521 | -12.931 | *<.001* | <.001 |
| Chile-France | 225.255 | 561.688 | 0.401 | *0.688* | 1.000 |
| Chile-South Africa | 264.580 | 555.570 | 0.476 | *0.634* | 1.000 |
| Chile-Portugal | 369.633 | 534.256 | 0.692 | *0.489* | 1.000 |
| Chile-Ireland | 433.217 | 566.268 | 0.765 | *0.444* | 1.000 |
| Chile-Israel | 571.467 | 595.832 | 0.959 | *0.338* | 1.000 |
| Chile-United Kingdom | 793.192 | 588.692 | 1.347 | *0.178* | 1.000 |
| Chile-Canada | 833.898 | 524.369 | 1.590 | *0.112* | 1.000 |
| Chile-South Korea | 1044.682 | 586.356 | 1.782 | *0.075* | 1.000 |
| Chile-Colombia | 1252.110 | 554.132 | 2.260 | *0.024* | 1.000 |
| Chile-New Zealand | 1663.822 | 517.044 | 3.218 | *0.001* | 0.420 |
| Chile-Australia | 1767.897 | 731.528 | 2.417 | *0.016* | 1.000 |
| Chile-United States of America | 2383.869 | 529.295 | 4.504 | *<.001* | 0.002 |
| Chile-Peru | 4199.514 | 521.400 | 8.054 | *<.001* | <.001 |
| Chile-Mexico | 6493.942 | 541.771 | 11.987 | *<.001* | <.001 |
| France-South Africa | -39.326 | 498.042 | -0.079 | *0.937* | 1.000 |
| France-Portugal | -144.379 | 474.149 | -0.305 | *0.761* | 1.000 |
| France-Ireland | -207.963 | 509.949 | -0.408 | *0.683* | 1.000 |
| France-Israel | -346.213 | 542.590 | -0.638 | *0.523* | 1.000 |
| France-United Kingdom | -567.938 | 534.739 | -1.062 | *0.288* | 1.000 |
| France-Canada | 608.643 | 462.980 | 1.315 | *0.189* | 1.000 |
| France-South Korea | -819.427 | 532.166 | -1.540 | *0.124* | 1.000 |
| France-Colombia | 1026.855 | 496.438 | 2.068 | *0.039* | 1.000 |
| France-New Zealand | -1438.568 | 454.667 | -3.164 | *0.002* | 0.506 |
| France-Australia | 1542.642 | 688.855 | 2.239 | *0.025* | 1.000 |
| France-United States of America | -2158.614 | 468.552 | -4.607 | *<.001* | 0.001 |
| France-Peru | -3974.259 | 459.616 | -8.647 | *<.001* | <.001 |
| France-Mexico | -6268.688 | 482.602 | -12.989 | *<.001* | <.001 |
| South Africa-Portugal | 105.053 | 466.886 | 0.225 | *0.822* | 1.000 |
| South Africa-Ireland | 168.637 | 503.202 | 0.335 | *0.738* | 1.000 |
| South Africa-Israel | 306.887 | 536.255 | 0.572 | *0.567* | 1.000 |
| South Africa-United Kingdom | -528.612 | 528.310 | -1.001 | *0.317* | 1.000 |
| South Africa-Canada | 569.318 | 455.539 | 1.250 | *0.211* | 1.000 |
| South Africa-South Korea | -780.101 | 525.706 | -1.484 | *0.138* | 1.000 |
| South Africa-Colombia | 987.530 | 489.506 | 2.017 | *0.044* | 1.000 |
| South Africa-New Zealand | 1399.242 | 447.088 | 3.130 | *0.002* | 0.569 |
| South Africa-Australia | 1503.317 | 683.876 | 2.198 | *0.028* | 1.000 |
| South Africa-United States of America | -2119.289 | 461.201 | -4.595 | *<.001* | 0.001 |
| South Africa-Peru | 3934.933 | 452.119 | 8.703 | *<.001* | <.001 |
| South Africa-Mexico | 6229.362 | 475.468 | 13.102 | *<.001* | <.001 |
| Portugal-Ireland | 63.584 | 479.566 | 0.133 | *0.895* | 1.000 |
| Portugal-Israel | 201.834 | 514.141 | 0.393 | *0.695* | 1.000 |
| Portugal-United Kingdom | -423.559 | 505.848 | -0.837 | *0.402* | 1.000 |
| Portugal-Canada | 464.265 | 429.286 | 1.081 | *0.279* | 1.000 |
| Portugal-South Korea | -675.048 | 503.128 | -1.342 | *0.180* | 1.000 |
| Portugal-Colombia | 882.477 | 465.174 | 1.897 | *0.058* | 1.000 |
| Portugal-New Zealand | 1294.189 | 420.308 | 3.079 | *0.002* | 0.675 |
| Portugal-Australia | 1398.263 | 666.676 | 2.097 | *0.036* | 1.000 |
| Portugal-United States of America | -2014.236 | 435.290 | -4.627 | *<.001* | 0.001 |
| Portugal-Peru | 3829.880 | 425.656 | 8.998 | *<.001* | <.001 |
| Portugal-Mexico | 6124.309 | 450.378 | 13.598 | *<.001* | <.001 |
| Ireland-Israel | -138.250 | 547.331 | -0.253 | *0.801* | 1.000 |
| Ireland-United Kingdom | -359.975 | 539.548 | -0.667 | *0.505* | 1.000 |
| Ireland-Canada | 400.681 | 468.526 | 0.855 | *0.392* | 1.000 |
| Ireland-South Korea | -611.464 | 536.999 | -1.139 | *0.255* | 1.000 |
| Ireland-Colombia | 818.893 | 501.614 | 1.633 | *0.103* | 1.000 |
| Ireland-New Zealand | -1230.605 | 460.314 | -2.673 | *0.008* | 1.000 |
| Ireland-Australia | 1334.679 | 692.595 | 1.927 | *0.054* | 1.000 |
| Ireland-United States of America | -1950.652 | 474.034 | -4.115 | *<.001* | 0.013 |
| Ireland-Peru | -3766.296 | 465.202 | -8.096 | *<.001* | <.001 |
| Ireland-Mexico | -6060.725 | 487.925 | -12.421 | *<.001* | <.001 |
| Israel-United Kingdom | -221.725 | 570.499 | -0.389 | *0.698* | 1.000 |
| Israel-Canada | 262.431 | 503.859 | 0.521 | *0.602* | 1.000 |
| Israel-South Korea | -473.214 | 568.088 | -0.833 | *0.405* | 1.000 |
| Israel-Colombia | 680.643 | 534.765 | 1.273 | *0.203* | 1.000 |
| Israel-New Zealand | -1092.355 | 496.231 | -2.201 | *0.028* | 1.000 |
| Israel-Australia | 1196.429 | 716.969 | 1.669 | *0.095* | 1.000 |
| Israel-United States of America | -1812.402 | 508.984 | -3.561 | *<.001* | 0.120 |
| Israel-Peru | -3628.046 | 500.769 | -7.245 | *<.001* | <.001 |
| Israel-Mexico | -5922.475 | 521.946 | -11.347 | *<.001* | <.001 |
| United Kingdom-Canada | 40.706 | 495.394 | 0.082 | *0.935* | 1.000 |
| United Kingdom-South Korea | 251.489 | 560.594 | 0.449 | *0.654* | 1.000 |
| United Kingdom-Colombia | 458.918 | 526.797 | 0.871 | *0.384* | 1.000 |
| United Kingdom-New Zealand | 870.630 | 487.634 | 1.785 | *0.074* | 1.000 |
| United Kingdom-Australia | 974.705 | 711.046 | 1.371 | *0.170* | 1.000 |
| United Kingdom-United States of America | -1590.677 | 500.606 | -3.178 | *0.001* | 0.483 |
| United Kingdom-Peru | 3406.322 | 492.251 | 6.920 | *<.001* | <.001 |
| United Kingdom-Mexico | 5700.750 | 513.779 | 11.096 | *<.001* | <.001 |
| Canada-South Korea | -210.783 | 492.616 | -0.428 | *0.669* | 1.000 |
| Canada-Colombia | -418.212 | 453.784 | -0.922 | *0.357* | 1.000 |
| Canada-New Zealand | -829.924 | 407.666 | -2.036 | *0.042* | 1.000 |
| Canada-Australia | 933.999 | 658.780 | 1.418 | *0.156* | 1.000 |
| Canada-United States of America | -1549.971 | 423.097 | -3.663 | *<.001* | 0.081 |
| Canada-Peru | -3365.616 | 413.178 | -8.146 | *<.001* | <.001 |
| Canada-Mexico | -5660.044 | 438.604 | -12.905 | *<.001* | <.001 |
| South Korea-Colombia | 207.428 | 524.186 | 0.396 | *0.692* | 1.000 |
| South Korea-New Zealand | 619.141 | 484.812 | 1.277 | *0.202* | 1.000 |
| South Korea-Australia | 723.215 | 709.113 | 1.020 | *0.308* | 1.000 |
| South Korea-United States of America | -1339.188 | 497.857 | -2.690 | *0.007* | 1.000 |
| South Korea-Peru | 3154.832 | 489.455 | 6.446 | *<.001* | <.001 |
| South Korea-Mexico | 5449.261 | 511.101 | 10.662 | *<.001* | <.001 |
| Colombia-New Zealand | -411.712 | 445.300 | -0.925 | *0.355* | 1.000 |
| Colombia-Australia | 515.787 | 682.708 | 0.756 | *0.450* | 1.000 |
| Colombia-United States of America | -1131.759 | 459.468 | -2.463 | *0.014* | 1.000 |
| Colombia-Peru | -2947.404 | 450.351 | -6.545 | *<.001* | <.001 |
| Colombia-Mexico | -5241.832 | 473.787 | -11.064 | *<.001* | <.001 |
| New Zealand-Australia | 104.074 | 652.964 | 0.159 | *0.873* | 1.000 |
| New Zealand-United States of America | -720.047 | 413.984 | -1.739 | *0.082* | 1.000 |
| New Zealand-Peru | -2535.691 | 403.841 | -6.279 | *<.001* | <.001 |
| New Zealand-Mexico | 4830.120 | 429.820 | 11.238 | *<.001* | <.001 |
| Australia-United States of America | -615.972 | 662.708 | -0.929 | *0.353* | 1.000 |
| Australia-Peru | -2431.617 | 656.420 | -3.704 | *<.001* | 0.069 |
| Australia-Mexico | -4726.046 | 672.714 | -7.025 | *<.001* | <.001 |
| United States of America-Peru | 1815.645 | 419.412 | 4.329 | *<.001* | 0.005 |
| United States of America-Mexico | 4110.073 | 444.482 | 9.247 | *<.001* | <.001 |
| Peru-Mexico | 2294.429 | 435.051 | 5.274 | *<.001* | <.001 |

Note. a = Significance values have been adjusted by the Bonferroni correction for multiple tests.

Table M. Country-based Pairwise Comparisons in the Kruskal-Wallis Test on the Sexual Abuse History Questionnaire – Adult Subscale (SAHQ)

| **Country comparisons** | **Test statistic** | **Standard error** | **Standardized test statistic**  **(z)** | ***p*-value** | **Adjusted *p*-value ^a^** |
| --- | --- | --- | --- | --- | --- |
| Colombia-North Macedonia | -743.226 | 617.354 | -1.204 | *0.229* | 1.000 |
| Colombia-Hungary | -784.305 | 423.814 | -1.851 | *0.064* | 1.000 |
| Colombia-South Korea | -920.144 | 587.599 | -1.566 | *0.117* | 1.000 |
| Colombia-Malaysia | -1805.747 | 625.552 | -2.887 | *0.004* | 1.000 |
| Colombia-Chile | -1894.009 | 627.201 | -3.02 | *0.003* | 0.822 |
| Colombia-Belgium | 2540.394 | 767.147 | 3.311 | *<.001* | 0.302 |
| Colombia-Germany | -2673.015 | 489.961 | -5.456 | *<.001* | <.001 |
| Colombia-Peru | -3444.231 | 514.259 | -6.697 | *<.001* | <.001 |
| Colombia-Spain | -3537.186 | 523.404 | -6.758 | *<.001* | <.001 |
| Colombia-Portugal | -4461.37 | 527.797 | -8.453 | *<.001* | <.001 |
| Colombia-Italy | -4603.837 | 520.809 | -8.84 | *<.001* | <.001 |
| Colombia-Israel | -5014.028 | 605.169 | -8.285 | *<.001* | <.001 |
| Colombia-Croatia | -5360.573 | 520.713 | -10.295 | *<.001* | <.001 |
| Colombia-Austria | 5431.453 | 726.085 | 7.48 | *<.001* | <.001 |
| Colombia-South Africa | -5457.275 | 554.361 | -9.844 | *<.001* | <.001 |
| Colombia-Poland | -5545.738 | 426.758 | -12.995 | *<.001* | <.001 |
| Colombia-France | -5618.449 | 564.623 | -9.951 | *<.001* | <.001 |
| Colombia-Switzerland | -5774.379 | 630.401 | -9.16 | *<.001* | <.001 |
| Colombia-Mexico | -7717.231 | 540.314 | -14.283 | *<.001* | <.001 |
| Colombia-Australia | 8285.567 | 771.221 | 10.743 | *<.001* | <.001 |
| Colombia-United States of America | -8740.905 | 520.094 | -16.806 | *<.001* | <.001 |
| Colombia-United Kingdom | -8913.765 | 594.643 | -14.99 | *<.001* | <.001 |
| Colombia-Canada | 9380.607 | 513.573 | 18.265 | *<.001* | <.001 |
| Colombia-New Zealand | -10257.723 | 503.571 | -20.37 | *<.001* | <.001 |
| Colombia-Ireland | -10578.716 | 565.945 | -18.692 | *<.001* | <.001 |
| North Macedonia-Hungary | 41.08 | 502.733 | 0.082 | *0.935* | 1.000 |
| North Macedonia-South Korea | -176.919 | 646.834 | -0.274 | *0.784* | 1.000 |
| North Macedonia-Malaysia | 1062.521 | 681.497 | 1.559 | *0.119* | 1.000 |
| North Macedonia-Chile | -1150.783 | 683.01 | -1.685 | *0.092* | 1.000 |
| North Macedonia-Belgium | 1797.168 | 813.411 | 2.209 | *0.027* | 1.000 |
| North Macedonia-Germany | 1929.79 | 559.628 | 3.448 | *<.001* | 0.183 |
| North Macedonia-Peru | -2701.006 | 581.02 | -4.649 | *<.001* | 0.001 |
| North Macedonia-Spain | -2793.96 | 589.13 | -4.743 | *<.001* | 0.001 |
| North Macedonia-Portugal | -3718.145 | 593.036 | -6.27 | *<.001* | <.001 |
| North Macedonia-Italy | 3860.611 | 586.826 | 6.579 | *<.001* | <.001 |
| North Macedonia-Israel | 4270.803 | 662.836 | 6.443 | *<.001* | <.001 |
| North Macedonia-Croatia | 4617.347 | 586.741 | 7.869 | *<.001* | <.001 |
| North Macedonia-Austria | 4688.228 | 774.804 | 6.051 | *<.001* | <.001 |
| North Macedonia-South Africa | -4714.049 | 616.797 | -7.643 | *<.001* | <.001 |
| North Macedonia-Poland | -4802.512 | 505.217 | -9.506 | *<.001* | <.001 |
| North Macedonia-France | 4875.224 | 626.036 | 7.787 | *<.001* | <.001 |
| North Macedonia-Switzerland | -5031.153 | 685.95 | -7.335 | *<.001* | <.001 |
| North Macedonia-Mexico | 6974.005 | 604.203 | 11.542 | *<.001* | <.001 |
| North Macedonia-Australia | 7542.342 | 817.254 | 9.229 | *<.001* | <.001 |
| North Macedonia-United States of America | -7997.68 | 586.191 | -13.643 | *<.001* | <.001 |
| North Macedonia-United Kingdom | -8170.539 | 653.24 | -12.508 | *<.001* | <.001 |
| North Macedonia-Canada | 8637.381 | 580.413 | 14.881 | *<.001* | <.001 |
| North Macedonia-New Zealand | 9514.497 | 571.582 | 16.646 | *<.001* | <.001 |
| North Macedonia-Ireland | 9835.49 | 627.229 | 15.681 | *<.001* | <.001 |
| Hungary-South Korea | -135.839 | 465.711 | -0.292 | *0.771* | 1.000 |
| Hungary-Malaysia | -1021.442 | 512.768 | -1.992 | *0.046* | 1.000 |
| Hungary-Chile | -1109.703 | 514.778 | -2.156 | *0.031* | 1.000 |
| Hungary-Belgium | 1756.088 | 678.329 | 2.589 | *0.01* | 1.000 |
| Hungary-Germany | 1888.71 | 334.18 | 5.652 | *<.001* | <.001 |
| Hungary-Peru | -2659.926 | 368.887 | -7.211 | *<.001* | <.001 |
| Hungary-Spain | -2752.881 | 381.533 | -7.215 | *<.001* | <.001 |
| Hungary-Portugal | -3677.065 | 387.537 | -9.488 | *<.001* | <.001 |
| Hungary-Italy | -3819.531 | 377.965 | -10.106 | *<.001* | <.001 |
| Hungary-Israel | -4229.723 | 487.693 | -8.673 | *<.001* | <.001 |
| Hungary-Croatia | 4576.267 | 377.833 | 12.112 | *<.001* | <.001 |
| Hungary-Austria | 4647.148 | 631.518 | 7.359 | *<.001* | <.001 |
| Hungary-South Africa | -4672.97 | 423.003 | -11.047 | *<.001* | <.001 |
| Hungary-Poland | -4761.432 | 231.813 | -20.54 | *<.001* | <.001 |
| Hungary-France | 4834.144 | 436.365 | 11.078 | *<.001* | <.001 |
| Hungary-Switzerland | -4990.074 | 518.672 | -9.621 | *<.001* | <.001 |
| Hungary-Mexico | -6932.925 | 404.418 | -17.143 | *<.001* | <.001 |
| Hungary-Australia | 7501.262 | 682.933 | 10.984 | *<.001* | <.001 |
| Hungary-United States of America | -7956.6 | 376.978 | -21.106 | *<.001* | <.001 |
| Hungary-United Kingdom | -8129.459 | 474.569 | -17.13 | *<.001* | <.001 |
| Hungary-Canada | 8596.302 | 367.929 | 23.364 | *<.001* | <.001 |
| Hungary-New Zealand | -9473.418 | 353.835 | -26.774 | *<.001* | <.001 |
| Hungary-Ireland | -9794.411 | 438.074 | -22.358 | *<.001* | <.001 |
| South Korea-Malaysia | 885.603 | 654.664 | 1.353 | *0.176* | 1.000 |
| South Korea-Chile | -973.864 | 656.239 | -1.484 | *0.138* | 1.000 |
| South Korea-Belgium | 1620.249 | 791.065 | 2.048 | *0.041* | 1.000 |
| South Korea-Germany | 1752.871 | 526.621 | 3.329 | *<.001* | 0.284 |
| South Korea-Peru | 2524.087 | 549.3 | 4.595 | *<.001* | 0.001 |
| South Korea-Spain | -2617.042 | 557.872 | -4.691 | *<.001* | 0.001 |
| South Korea-Portugal | 3541.226 | 561.995 | 6.301 | *<.001* | <.001 |
| South Korea-Italy | 3683.692 | 555.438 | 6.632 | *<.001* | <.001 |
| South Korea-Israel | 4093.884 | 635.215 | 6.445 | *<.001* | <.001 |
| South Korea-Croatia | 4440.428 | 555.348 | 7.996 | *<.001* | <.001 |
| South Korea-Austria | 4511.309 | 751.311 | 6.005 | *<.001* | <.001 |
| South Korea-South Africa | 4537.131 | 587.014 | 7.729 | *<.001* | <.001 |
| South Korea-Poland | 4625.593 | 468.392 | 9.875 | *<.001* | <.001 |
| South Korea-France | 4698.305 | 596.714 | 7.874 | *<.001* | <.001 |
| South Korea-Switzerland | -4854.235 | 659.299 | -7.363 | *<.001* | <.001 |
| South Korea-Mexico | 6797.086 | 573.766 | 11.846 | *<.001* | <.001 |
| South Korea-Australia | 7365.423 | 795.016 | 9.264 | *<.001* | <.001 |
| South Korea-United States of America | -7820.761 | 554.767 | -14.097 | *<.001* | <.001 |
| South Korea-United Kingdom | -7993.62 | 625.195 | -12.786 | *<.001* | <.001 |
| South Korea-Canada | 8460.463 | 548.658 | 15.42 | *<.001* | <.001 |
| South Korea-New Zealand | 9337.579 | 539.308 | 17.314 | *<.001* | <.001 |
| South Korea-Ireland | 9658.572 | 597.965 | 16.152 | *<.001* | <.001 |
| Malaysia-Chile | -88.262 | 690.43 | -0.128 | *0.898* | 1.000 |
| Malaysia-Belgium | 734.647 | 819.651 | 0.896 | *0.37* | 1.000 |
| Malaysia-Germany | 867.269 | 568.66 | 1.525 | *0.127* | 1.000 |
| Malaysia-Peru | -1638.485 | 589.724 | -2.778 | *0.005* | 1.000 |
| Malaysia-Spain | -1731.439 | 597.716 | -2.897 | *0.004* | 1.000 |
| Malaysia-Portugal | -2655.624 | 601.567 | -4.415 | *<.001* | 0.003 |
| Malaysia-Italy | 2798.09 | 595.445 | 4.699 | *<.001* | 0.001 |
| Malaysia-Israel | 3208.282 | 670.479 | 4.785 | *<.001* | 0.001 |
| Malaysia-Croatia | 3554.826 | 595.361 | 5.971 | *<.001* | <.001 |
| Malaysia-Austria | 3625.707 | 781.353 | 4.64 | *<.001* | 0.001 |
| Malaysia-South Africa | -3651.528 | 625.003 | -5.842 | *<.001* | <.001 |
| Malaysia-Poland | -3739.991 | 515.203 | -7.259 | *<.001* | <.001 |
| Malaysia-France | 3812.703 | 634.122 | 6.013 | *<.001* | <.001 |
| Malaysia-Switzerland | -3968.632 | 693.338 | -5.724 | *<.001* | <.001 |
| Malaysia-Mexico | -5911.484 | 612.577 | -9.65 | *<.001* | <.001 |
| Malaysia-Australia | 6479.821 | 823.465 | 7.869 | *<.001* | <.001 |
| Malaysia-United States of America | -6935.158 | 594.819 | -11.659 | *<.001* | <.001 |
| Malaysia-United Kingdom | -7108.018 | 660.994 | -10.754 | *<.001* | <.001 |
| Malaysia-Canada | 7574.86 | 589.126 | 12.858 | *<.001* | <.001 |
| Malaysia-New Zealand | -8451.976 | 580.428 | -14.562 | *<.001* | <.001 |
| Malaysia-Ireland | 8772.969 | 635.3 | 13.809 | *<.001* | <.001 |
| Chile-Belgium | 646.385 | 820.91 | 0.787 | *0.431* | 1.000 |
| Chile-Germany | 779.007 | 570.473 | 1.366 | *0.172* | 1.000 |
| Chile-Peru | 1550.223 | 591.473 | 2.621 | *0.009* | 1.000 |
| Chile-Spain | 1643.177 | 599.441 | 2.741 | *0.006* | 1.000 |
| Chile-Portugal | 2567.362 | 603.281 | 4.256 | *<.001* | 0.007 |
| Chile-Italy | 2709.828 | 597.177 | 4.538 | *<.001* | 0.002 |
| Chile-Israel | 3120.02 | 672.017 | 4.643 | *<.001* | 0.001 |
| Chile-Croatia | 3466.564 | 597.093 | 5.806 | *<.001* | <.001 |
| Chile-Austria | 3537.445 | 782.673 | 4.52 | *<.001* | 0.002 |
| Chile-South Africa | 3563.266 | 626.653 | 5.686 | *<.001* | <.001 |
| Chile-Poland | 3651.729 | 517.204 | 7.061 | *<.001* | <.001 |
| Chile-France | 3724.441 | 635.749 | 5.858 | *<.001* | <.001 |
| Chile-Switzerland | 3880.37 | 694.826 | 5.585 | *<.001* | <.001 |
| Chile-Mexico | 5823.222 | 614.261 | 9.48 | *<.001* | <.001 |
| Chile-Australia | 6391.559 | 824.718 | 7.75 | *<.001* | <.001 |
| Chile-United States of America | 6846.897 | 596.553 | 11.477 | *<.001* | <.001 |
| Chile-United Kingdom | 7019.756 | 662.554 | 10.595 | *<.001* | <.001 |
| Chile-Canada | 7486.598 | 590.876 | 12.67 | *<.001* | <.001 |
| Chile-New Zealand | 8363.714 | 582.204 | 14.366 | *<.001* | <.001 |
| Chile-Ireland | 8684.707 | 636.923 | 13.635 | *<.001* | <.001 |
| Belgium-Germany | -132.622 | 721.507 | -0.184 | *0.854* | 1.000 |
| Belgium-Peru | -903.838 | 738.223 | -1.224 | *0.221* | 1.000 |
| Belgium-Spain | -996.792 | 744.623 | -1.339 | *0.181* | 1.000 |
| Belgium-Portugal | -1920.977 | 747.718 | -2.569 | *0.01* | 1.000 |
| Belgium-Italy | -2063.443 | 742.801 | -2.778 | *0.005* | 1.000 |
| Belgium-Israel | -2473.635 | 804.202 | -3.076 | *0.002* | 0.682 |
| Belgium-Croatia | -2820.179 | 742.734 | -3.797 | *<.001* | 0.048 |
| Belgium-Austria | 2891.06 | 898.727 | 3.217 | *0.001* | 0.421 |
| Belgium-South Africa | -2916.881 | 766.699 | -3.804 | *<.001* | 0.046 |
| Belgium-Poland | -3005.344 | 680.172 | -4.419 | *<.001* | 0.003 |
| Belgium-France | -3078.056 | 774.152 | -3.976 | *<.001* | 0.023 |
| Belgium-Switzerland | -3233.985 | 823.357 | -3.928 | *<.001* | 0.028 |
| Belgium-Mexico | -5176.837 | 756.605 | -6.842 | *<.001* | <.001 |
| Belgium-Australia | 5745.174 | 935.571 | 6.141 | *<.001* | <.001 |
| Belgium-United States of America | -6200.512 | 742.3 | -8.353 | *<.001* | <.001 |
| Belgium-United Kingdom | -6373.371 | 796.311 | -8.004 | *<.001* | <.001 |
| Belgium-Canada | -6840.213 | 737.746 | -9.272 | *<.001* | <.001 |
| Belgium-New Zealand | -7717.329 | 730.819 | -10.56 | *<.001* | <.001 |
| Belgium-Ireland | -8038.322 | 775.116 | -10.37 | *<.001* | <.001 |
| Germany-Peru | -771.216 | 443.306 | -1.74 | *0.082* | 1.000 |
| Germany-Spain | -864.17 | 453.884 | -1.904 | *0.057* | 1.000 |
| Germany-Portugal | -1788.355 | 458.942 | -3.897 | *<.001* | 0.032 |
| Germany-Italy | -1930.821 | 450.889 | -4.282 | *<.001* | 0.006 |
| Germany-Israel | -2341.013 | 546.157 | -4.286 | *<.001* | 0.006 |
| Germany-Croatia | 2687.557 | 450.778 | 5.962 | *<.001* | <.001 |
| Germany-Austria | 2758.438 | 677.685 | 4.07 | *<.001* | 0.015 |
| Germany-South Africa | -2784.259 | 489.259 | -5.691 | *<.001* | <.001 |
| Germany-Poland | -2872.722 | 337.906 | -8.502 | *<.001* | <.001 |
| Germany-France | 2945.434 | 500.856 | 5.881 | *<.001* | <.001 |
| Germany-Switzerland | -3101.363 | 573.99 | -5.403 | *<.001* | <.001 |
| Germany-Mexico | -5044.215 | 473.283 | -10.658 | *<.001* | <.001 |
| Germany-Australia | 5612.552 | 725.837 | 7.733 | *<.001* | <.001 |
| Germany-United States of America | -6067.89 | 450.062 | -13.482 | *<.001* | <.001 |
| Germany-United Kingdom | -6240.749 | 534.47 | -11.677 | *<.001* | <.001 |
| Germany-Canada | 6707.591 | 442.51 | 15.158 | *<.001* | <.001 |
| Germany-New Zealand | -7584.708 | 430.862 | -17.604 | *<.001* | <.001 |
| Germany-Ireland | -7905.701 | 502.346 | -15.738 | *<.001* | <.001 |
| Peru-Spain | -92.954 | 480.012 | -0.194 | *0.846* | 1.000 |
| Peru-Portugal | -1017.139 | 484.798 | -2.098 | *0.036* | 1.000 |
| Peru-Italy | 1159.605 | 477.181 | 2.43 | *0.015* | 1.000 |
| Peru-Israel | 1569.797 | 568.056 | 2.763 | *0.006* | 1.000 |
| Peru-Croatia | 1916.341 | 477.076 | 4.017 | *<.001* | 0.019 |
| Peru-Austria | 1987.222 | 695.456 | 2.857 | *0.004* | 1.000 |
| Peru-South Africa | -2013.043 | 513.591 | -3.92 | *<.001* | 0.029 |
| Peru-Poland | -2101.506 | 372.265 | -5.645 | *<.001* | <.001 |
| Peru-France | 2174.218 | 524.65 | 4.144 | *<.001* | 0.011 |
| Peru-Switzerland | -2330.147 | 594.865 | -3.917 | *<.001* | 0.029 |
| Peru-Mexico | 4272.999 | 498.395 | 8.574 | *<.001* | <.001 |
| Peru-Australia | 4841.336 | 742.456 | 6.521 | *<.001* | <.001 |
| Peru-United States of America | -5296.674 | 476.4 | -11.118 | *<.001* | <.001 |
| Peru-United Kingdom | -5469.533 | 556.83 | -9.823 | *<.001* | <.001 |
| Peru-Canada | 5936.375 | 469.272 | 12.65 | *<.001* | <.001 |
| Peru-New Zealand | 6813.492 | 458.305 | 14.867 | *<.001* | <.001 |
| Peru-Ireland | 7134.485 | 526.073 | 13.562 | *<.001* | <.001 |
| Spain-Portugal | 924.185 | 494.489 | 1.869 | *0.062* | 1.000 |
| Spain-Italy | 1066.651 | 487.023 | 2.19 | *0.029* | 1.000 |
| Spain-Israel | 1476.843 | 576.349 | 2.562 | *0.01* | 1.000 |
| Spain-Croatia | 1823.387 | 486.92 | 3.745 | *<.001* | 0.059 |
| Spain-Austria | 1894.267 | 702.245 | 2.697 | *0.007* | 1.000 |
| Spain-South Africa | 1920.089 | 522.748 | 3.673 | *<.001* | 0.078 |
| Spain-Poland | 2008.552 | 384.8 | 5.22 | *<.001* | <.001 |
| Spain-France | 2081.264 | 533.618 | 3.9 | *<.001* | 0.031 |
| Spain-Switzerland | -2237.193 | 602.789 | -3.711 | *<.001* | 0.067 |
| Spain-Mexico | 4180.045 | 507.827 | 8.231 | *<.001* | <.001 |
| Spain-Australia | 4748.382 | 748.82 | 6.341 | *<.001* | <.001 |
| Spain-United States of America | -5203.719 | 486.258 | -10.702 | *<.001* | <.001 |
| Spain-United Kingdom | -5376.579 | 565.287 | -9.511 | *<.001* | <.001 |
| Spain-Canada | 5843.421 | 479.277 | 12.192 | *<.001* | <.001 |
| Spain-New Zealand | 6720.537 | 468.544 | 14.343 | *<.001* | <.001 |
| Spain-Ireland | 7041.53 | 535.016 | 13.161 | *<.001* | <.001 |
| Portugal-Italy | 142.466 | 491.741 | 0.29 | *0.772* | 1.000 |
| Portugal-Israel | 552.658 | 580.341 | 0.952 | *0.341* | 1.000 |
| Portugal-Croatia | 899.202 | 491.639 | 1.829 | *0.067* | 1.000 |
| Portugal-Austria | 970.083 | 705.526 | 1.375 | *0.169* | 1.000 |
| Portugal-South Africa | -995.904 | 527.146 | -1.889 | *0.059* | 1.000 |
| Portugal-Poland | 1084.367 | 390.754 | 2.775 | *0.006* | 1.000 |
| Portugal-France | 1157.079 | 537.927 | 2.151 | *0.031* | 1.000 |
| Portugal-Switzerland | -1313.008 | 606.607 | -2.165 | *0.03* | 1.000 |
| Portugal-Mexico | 3255.86 | 512.353 | 6.355 | *<.001* | <.001 |
| Portugal-Australia | 3824.197 | 751.897 | 5.086 | *<.001* | <.001 |
| Portugal-United States of America | -4279.535 | 490.983 | -8.716 | *<.001* | <.001 |
| Portugal-United Kingdom | -4452.394 | 569.357 | -7.82 | *<.001* | <.001 |
| Portugal-Canada | 4919.236 | 484.07 | 10.162 | *<.001* | <.001 |
| Portugal-New Zealand | 5796.352 | 473.446 | 12.243 | *<.001* | <.001 |
| Portugal-Ireland | 6117.346 | 539.315 | 11.343 | *<.001* | <.001 |
| Italy-Israel | 410.192 | 573.993 | 0.715 | *0.475* | 1.000 |
| Italy-Croatia | 756.736 | 484.13 | 1.563 | *0.118* | 1.000 |
| Italy-Austria | 827.617 | 700.313 | 1.182 | *0.237* | 1.000 |
| Italy-South Africa | -853.438 | 520.15 | -1.641 | *0.101* | 1.000 |
| Italy-Poland | -941.901 | 381.263 | -2.47 | *0.013* | 1.000 |
| Italy-France | 1014.613 | 531.073 | 1.91 | *0.056* | 1.000 |
| Italy-Switzerland | -1170.542 | 600.537 | -1.949 | *0.051* | 1.000 |
| Italy-Mexico | -3113.394 | 505.151 | -6.163 | *<.001* | <.001 |
| Italy-Australia | 3681.731 | 747.008 | 4.929 | *<.001* | <.001 |
| Italy-United States of America | -4137.069 | 483.463 | -8.557 | *<.001* | <.001 |
| Italy-United Kingdom | -4309.928 | 562.885 | -7.657 | *<.001* | <.001 |
| Italy-Canada | 4776.77 | 476.441 | 10.026 | *<.001* | <.001 |
| Italy-New Zealand | -5653.886 | 465.643 | -12.142 | *<.001* | <.001 |
| Italy-Ireland | 5974.879 | 532.478 | 11.221 | *<.001* | <.001 |
| Israel-Croatia | 346.544 | 573.906 | 0.604 | *0.546* | 1.000 |
| Israel-Austria | 417.425 | 765.131 | 0.546 | *0.585* | 1.000 |
| Israel-South Africa | -443.246 | 604.601 | -0.733 | *0.463* | 1.000 |
| Israel-Poland | -531.709 | 490.253 | -1.085 | *0.278* | 1.000 |
| Israel-France | 604.421 | 614.024 | 0.984 | *0.325* | 1.000 |
| Israel-Switzerland | -760.35 | 675.005 | -1.126 | *0.26* | 1.000 |
| Israel-Mexico | -2703.202 | 591.747 | -4.568 | *<.001* | 0.002 |
| Israel-Australia | 3271.539 | 808.089 | 4.048 | *<.001* | 0.017 |
| Israel-United States of America | -3726.877 | 573.344 | -6.5 | *<.001* | <.001 |
| Israel-United Kingdom | -3899.736 | 641.737 | -6.077 | *<.001* | <.001 |
| Israel-Canada | 4366.578 | 567.435 | 7.695 | *<.001* | <.001 |
| Israel-New Zealand | -5243.694 | 558.4 | -9.391 | *<.001* | <.001 |
| Israel-Ireland | 5564.687 | 615.239 | 9.045 | *<.001* | <.001 |
| Croatia-Austria | 70.881 | 700.242 | 0.101 | *0.919* | 1.000 |
| Croatia-South Africa | -96.702 | 520.054 | -0.186 | *0.852* | 1.000 |
| Croatia-Poland | -185.165 | 381.132 | -0.486 | *0.627* | 1.000 |
| Croatia-France | -257.877 | 530.979 | -0.486 | *0.627* | 1.000 |
| Croatia-Switzerland | -413.806 | 600.454 | -0.689 | *0.491* | 1.000 |
| Croatia-Mexico | -2356.658 | 505.053 | -4.666 | *<.001* | 0.001 |
| Croatia-Australia | 2924.995 | 746.941 | 3.916 | *<.001* | 0.029 |
| Croatia-United States of America | -3380.333 | 483.36 | -6.993 | *<.001* | <.001 |
| Croatia-United Kingdom | -3553.192 | 562.796 | -6.313 | *<.001* | <.001 |
| Croatia-Canada | 4020.034 | 476.336 | 8.439 | *<.001* | <.001 |
| Croatia-New Zealand | -4897.15 | 465.536 | -10.519 | *<.001* | <.001 |
| Croatia-Ireland | -5218.143 | 532.384 | -9.801 | *<.001* | <.001 |
| Austria-South Africa | -25.821 | 725.612 | -0.036 | *0.972* | 1.000 |
| Austria-Poland | -114.284 | 633.497 | -0.18 | *0.857* | 1.000 |
| Austria-France | -186.996 | 733.482 | -0.255 | *0.799* | 1.000 |
| Austria-Switzerland | -342.926 | 785.24 | -0.437 | *0.662* | 1.000 |
| Austria-Mexico | -2285.777 | 714.937 | -3.197 | *0.001* | 0.451 |
| Austria-Australia | 2854.114 | 902.207 | 3.163 | *0.002* | 0.507 |
| Austria-United States of America | -3309.452 | 699.781 | -4.729 | *<.001* | 0.001 |
| Austria-United Kingdom | -3482.311 | 756.833 | -4.601 | *<.001* | 0.001 |
| Austria-Canada | -3949.153 | 694.948 | -5.683 | *<.001* | <.001 |
| Austria-New Zealand | -4826.27 | 687.59 | -7.019 | *<.001* | <.001 |
| Austria-Ireland | -5147.263 | 734.5 | -7.008 | *<.001* | <.001 |
| South Africa-Poland | 88.463 | 425.952 | 0.208 | *0.835* | 1.000 |
| South Africa-France | 161.175 | 564.014 | 0.286 | *0.775* | 1.000 |
| South Africa-Switzerland | -317.104 | 629.856 | -0.503 | *0.615* | 1.000 |
| South Africa-Mexico | 2259.956 | 539.678 | 4.188 | *<.001* | 0.009 |
| South Africa-Australia | 2828.293 | 770.776 | 3.669 | *<.001* | 0.079 |
| South Africa-United States of America | -3283.63 | 519.433 | -6.322 | *<.001* | <.001 |
| South Africa-United Kingdom | -3456.49 | 594.065 | -5.818 | *<.001* | <.001 |
| South Africa-Canada | 3923.332 | 512.904 | 7.649 | *<.001* | <.001 |
| South Africa-New Zealand | 4800.448 | 502.889 | 9.546 | *<.001* | <.001 |
| South Africa-Ireland | 5121.441 | 565.338 | 9.059 | *<.001* | <.001 |
| Poland-France | 72.712 | 439.224 | 0.166 | *0.869* | 1.000 |
| Poland-Switzerland | -228.641 | 521.08 | -0.439 | *0.661* | 1.000 |
| Poland-Mexico | 2171.493 | 407.502 | 5.329 | *<.001* | <.001 |
| Poland-Australia | 2739.83 | 684.763 | 4.001 | *<.001* | 0.020 |
| Poland-United States of America | -3195.168 | 380.285 | -8.402 | *<.001* | <.001 |
| Poland-United Kingdom | -3368.027 | 477.199 | -7.058 | *<.001* | <.001 |
| Poland-Canada | 3834.869 | 371.316 | 10.328 | *<.001* | <.001 |
| Poland-New Zealand | 4711.985 | 357.356 | 13.186 | *<.001* | <.001 |
| Poland-Ireland | 5032.978 | 440.922 | 11.415 | *<.001* | <.001 |
| France-Switzerland | -155.929 | 638.907 | -0.244 | *0.807* | 1.000 |
| France-Mexico | -2098.781 | 550.213 | -3.814 | *<.001* | 0.044 |
| France-Australia | 2667.118 | 778.189 | 3.427 | *<.001* | 0.198 |
| France-United States of America | -3122.456 | 530.371 | -5.887 | *<.001* | <.001 |
| France-United Kingdom | -3295.315 | 603.652 | -5.459 | *<.001* | <.001 |
| France-Canada | 3762.157 | 523.978 | 7.18 | *<.001* | <.001 |
| France-New Zealand | -4639.274 | 514.179 | -9.023 | *<.001* | <.001 |
| France-Ireland | -4960.267 | 575.404 | -8.62 | *<.001* | <.001 |
| Switzerland-Mexico | 1942.852 | 617.529 | 3.146 | *0.002* | 0.538 |
| Switzerland-Australia | 2511.189 | 827.154 | 3.036 | *0.002* | 0.779 |
| Switzerland-United States of America | -2966.526 | 599.917 | -4.945 | *<.001* | <.001 |
| Switzerland-United Kingdom | -3139.386 | 665.585 | -4.717 | *<.001* | 0.001 |
| Switzerland-Canada | 3606.228 | 594.272 | 6.068 | *<.001* | <.001 |
| Switzerland-New Zealand | 4483.344 | 585.651 | 7.655 | *<.001* | <.001 |
| Switzerland-Ireland | 4804.337 | 640.075 | 7.506 | *<.001* | <.001 |
| Mexico-Australia | 568.337 | 760.735 | 0.747 | *0.455* | 1.000 |
| Mexico-United States of America | -1023.675 | 504.414 | -2.029 | *0.042* | 1.000 |
| Mexico-United Kingdom | -1196.534 | 580.978 | -2.06 | *0.039* | 1.000 |
| Mexico-Canada | 1663.376 | 497.687 | 3.342 | *<.001* | 0.270 |
| Mexico-New Zealand | -2540.492 | 487.36 | -5.213 | *<.001* | <.001 |
| Mexico-Ireland | 2861.485 | 551.57 | 5.188 | *<.001* | <.001 |
| Australia-United States of America | -455.338 | 746.509 | -0.61 | *0.542* | 1.000 |
| Australia-United Kingdom | -628.197 | 800.237 | -0.785 | *0.432* | 1.000 |
| Australia-Canada | -1095.039 | 741.981 | -1.476 | *0.14* | 1.000 |
| Australia-New Zealand | -1972.155 | 735.094 | -2.683 | *0.007* | 1.000 |
| Australia-Ireland | -2293.149 | 779.148 | -2.943 | *0.003* | 1.000 |
| United States of America-United Kingdom | 172.859 | 562.223 | 0.307 | *0.758* | 1.000 |
| United States of America-Canada | 639.702 | 475.659 | 1.345 | *0.179* | 1.000 |
| United States of America-New Zealand | 1516.818 | 464.842 | 3.263 | *0.001* | 0.358 |
| United States of America-Ireland | 1837.811 | 531.778 | 3.456 | *<.001* | 0.178 |
| United Kingdom-Canada | 466.842 | 556.196 | 0.839 | *0.401* | 1.000 |
| United Kingdom-New Zealand | 1343.958 | 546.974 | 2.457 | *0.014* | 1.000 |
| United Kingdom-Ireland | 1664.951 | 604.889 | 2.752 | *0.006* | 1.000 |
| Canada-New Zealand | -877.116 | 457.535 | -1.917 | *0.055* | 1.000 |
| Canada-Ireland | -1198.109 | 525.402 | -2.28 | *0.023* | 1.000 |
| New Zealand-Ireland | 320.993 | 515.63 | 0.623 | *0.534* | 1.000 |

Note. a = Significance values have been adjusted by the Bonferroni correction for multiple tests.

Table N. Country-based Pairwise Comparisons in the Kruskal-Wallis Test on the Sexual Abuse History Questionnaire

| **Country comparisons** | **Test statistic** | **Standard error** | **Standardized test statistic**  **(z)** | ***p*-value** | **Adjusted *p*-value ^a^** |
| --- | --- | --- | --- | --- | --- |
| North Macedonia-Hungary | 361.302 | 528.105 | 0.684 | *0.494* | 1.000 |
| North Macedonia-Malaysia | 2644.134 | 719.489 | 3.675 | *<.001* | 0.077 |
| North Macedonia-Colombia | 2889.125 | 645.485 | 4.476 | *<.001* | 0.002 |
| North Macedonia-Belgium | 2897.092 | 857.239 | 3.38 | *<.001* | 0.236 |
| North Macedonia-Germany | 2972.051 | 588.629 | 5.049 | *<.001* | <.001 |
| North Macedonia-Spain | -2980.463 | 620.531 | -4.803 | *<.001* | 0.001 |
| North Macedonia-Chile | -3325.137 | 719.489 | -4.622 | *<.001* | 0.001 |
| North Macedonia-South Korea | -3348.598 | 681.709 | -4.912 | *<.001* | <.001 |
| North Macedonia-Italy | 4808.689 | 617.615 | 7.786 | *<.001* | <.001 |
| North Macedonia-Croatia | 5007.29 | 618.241 | 8.099 | *<.001* | <.001 |
| North Macedonia-Austria | 5406.971 | 817.918 | 6.611 | *<.001* | <.001 |
| North Macedonia-Portugal | -5607.699 | 623.746 | -8.99 | *<.001* | <.001 |
| North Macedonia-Poland | -5765.127 | 531.463 | -10.848 | *<.001* | <.001 |
| North Macedonia-Israel | 5813.027 | 697.351 | 8.336 | *<.001* | <.001 |
| North Macedonia-Switzerland | -5947.538 | 723.053 | -8.226 | *<.001* | <.001 |
| North Macedonia-France | 6249.849 | 658.752 | 9.487 | *<.001* | <.001 |
| North Macedonia-South Africa | -6327.846 | 647.784 | -9.768 | *<.001* | <.001 |
| North Macedonia-Peru | -7276.551 | 607.516 | -11.978 | *<.001* | <.001 |
| North Macedonia-United Kingdom | -8981.001 | 687.627 | -13.061 | *<.001* | <.001 |
| North Macedonia-Australia | 9000.192 | 860.798 | 10.456 | *<.001* | <.001 |
| North Macedonia-Canada | 9786.546 | 611.352 | 16.008 | *<.001* | <.001 |
| North Macedonia-United States of America | -9875.541 | 617.172 | -16.001 | *<.001* | <.001 |
| North Macedonia-Ireland | 10358.724 | 660.324 | 15.687 | *<.001* | <.001 |
| North Macedonia-New Zealand | 10441.541 | 601.543 | 17.358 | *<.001* | <.001 |
| North Macedonia-Mexico | 11736.448 | 631.619 | 18.582 | *<.001* | <.001 |
| Hungary-Malaysia | -2282.832 | 542.814 | -4.206 | *<.001* | 0.008 |
| Hungary-Colombia | 2527.823 | 440.039 | 5.745 | *<.001* | <.001 |
| Hungary-Belgium | 2535.79 | 715.431 | 3.544 | *<.001* | 0.128 |
| Hungary-Germany | 2610.749 | 351.38 | 7.43 | *<.001* | <.001 |
| Hungary-Spain | -2619.161 | 402.545 | -6.506 | *<.001* | <.001 |
| Hungary-Chile | -2963.835 | 542.814 | -5.46 | *<.001* | <.001 |
| Hungary-South Korea | -2987.296 | 491.64 | -6.076 | *<.001* | <.001 |
| Hungary-Italy | -4447.387 | 398.035 | -11.173 | *<.001* | <.001 |
| Hungary-Croatia | 4645.988 | 399.005 | 11.644 | *<.001* | <.001 |
| Hungary-Austria | 5045.669 | 667.812 | 7.556 | *<.001* | <.001 |
| Hungary-Portugal | -5246.397 | 407.484 | -12.875 | *<.001* | <.001 |
| Hungary-Poland | -5403.825 | 243.795 | -22.165 | *<.001* | <.001 |
| Hungary-Israel | -5451.725 | 513.11 | -10.625 | *<.001* | <.001 |
| Hungary-Switzerland | -5586.236 | 547.531 | -10.203 | *<.001* | <.001 |
| Hungary-France | 5888.547 | 459.279 | 12.821 | *<.001* | <.001 |
| Hungary-South Africa | -5966.544 | 443.405 | -13.456 | *<.001* | <.001 |
| Hungary-Peru | -6915.249 | 382.176 | -18.094 | *<.001* | <.001 |
| Hungary-United Kingdom | -8619.699 | 499.814 | -17.246 | *<.001* | <.001 |
| Hungary-Australia | 8638.89 | 719.692 | 12.004 | *<.001* | <.001 |
| Hungary-Canada | 9425.244 | 388.246 | 24.276 | *<.001* | <.001 |
| Hungary-United States of America | -9514.239 | 397.348 | -23.944 | *<.001* | <.001 |
| Hungary-Ireland | -9997.422 | 461.532 | -21.661 | *<.001* | <.001 |
| Hungary-New Zealand | -10080.239 | 372.609 | -27.053 | *<.001* | <.001 |
| Hungary-Mexico | -11375.146 | 419.436 | -27.12 | *<.001* | <.001 |
| Malaysia-Colombia | 244.991 | 657.574 | 0.373 | *0.709* | 1.000 |
| Malaysia-Belgium | 252.957 | 866.378 | 0.292 | *0.77* | 1.000 |
| Malaysia-Germany | 327.917 | 601.861 | 0.545 | *0.586* | 1.000 |
| Malaysia-Spain | -336.328 | 633.097 | -0.531 | *0.595* | 1.000 |
| Malaysia-Chile | -681.003 | 730.353 | -0.932 | *0.351* | 1.000 |
| Malaysia-South Korea | -704.464 | 693.166 | -1.016 | *0.309* | 1.000 |
| Malaysia-Italy | 2164.555 | 630.239 | 3.435 | *<.001* | 0.193 |
| Malaysia-Croatia | 2363.156 | 630.852 | 3.746 | *<.001* | 0.058 |
| Malaysia-Austria | 2762.837 | 827.491 | 3.339 | *<.001* | 0.273 |
| Malaysia-Portugal | -2963.565 | 636.248 | -4.658 | *<.001* | 0.001 |
| Malaysia-Poland | -3120.992 | 546.081 | -5.715 | *<.001* | <.001 |
| Malaysia-Israel | 3168.893 | 708.555 | 4.472 | *<.001* | 0.003 |
| Malaysia-Switzerland | -3303.403 | 733.865 | -4.501 | *<.001* | 0.002 |
| Malaysia-France | 3605.715 | 670.601 | 5.377 | *<.001* | <.001 |
| Malaysia-South Africa | -3683.712 | 659.831 | -5.583 | *<.001* | <.001 |
| Malaysia-Peru | -4632.417 | 620.344 | -7.467 | *<.001* | <.001 |
| Malaysia-United Kingdom | -6336.867 | 698.987 | -9.066 | *<.001* | <.001 |
| Malaysia-Australia | 6356.058 | 869.9 | 7.307 | *<.001* | <.001 |
| Malaysia-Canada | 7142.412 | 624.102 | 11.444 | *<.001* | <.001 |
| Malaysia-United States of America | -7231.407 | 629.805 | -11.482 | *<.001* | <.001 |
| Malaysia-Ireland | 7714.59 | 672.146 | 11.478 | *<.001* | <.001 |
| Malaysia-New Zealand | -7797.406 | 614.496 | -12.689 | *<.001* | <.001 |
| Malaysia-Mexico | -9092.314 | 643.968 | -14.119 | *<.001* | <.001 |
| Colombia-Belgium | 7.966 | 805.976 | 0.01 | *0.992* | 1.000 |
| Colombia-Germany | -82.926 | 511.101 | -0.162 | *0.871* | 1.000 |
| Colombia-Spain | -91.337 | 547.539 | -0.167 | *0.868* | 1.000 |
| Colombia-Chile | -436.012 | 657.574 | -0.663 | *0.507* | 1.000 |
| Colombia-South Korea | -459.473 | 616.008 | -0.746 | *0.456* | 1.000 |
| Colombia-Italy | -1919.564 | 544.232 | -3.527 | *<.001* | 0.137 |
| Colombia-Croatia | -2118.165 | 544.941 | -3.887 | *<.001* | 0.033 |
| Colombia-Austria | 2517.846 | 764.022 | 3.296 | *<.001* | 0.319 |
| Colombia-Portugal | -2718.574 | 551.18 | -4.932 | *<.001* | <.001 |
| Colombia-Poland | -2876.001 | 444.063 | -6.477 | *<.001* | <.001 |
| Colombia-Israel | -2923.902 | 633.275 | -4.617 | *<.001* | 0.001 |
| Colombia-Switzerland | -3058.412 | 661.472 | -4.624 | *<.001* | 0.001 |
| Colombia-France | -3360.724 | 590.502 | -5.691 | *<.001* | <.001 |
| Colombia-South Africa | -3438.721 | 578.242 | -5.947 | *<.001* | <.001 |
| Colombia-Peru | -4387.426 | 532.742 | -8.236 | *<.001* | <.001 |
| Colombia-United Kingdom | -6091.876 | 622.551 | -9.785 | *<.001* | <.001 |
| Colombia-Australia | 6111.067 | 809.761 | 7.547 | *<.001* | <.001 |
| Colombia-Canada | 6897.421 | 537.113 | 12.842 | *<.001* | <.001 |
| Colombia-United States of America | -6986.416 | 543.729 | -12.849 | *<.001* | <.001 |
| Colombia-Ireland | -7469.599 | 592.256 | -12.612 | *<.001* | <.001 |
| Colombia-New Zealand | -7552.415 | 525.921 | -14.36 | *<.001* | <.001 |
| Colombia-Mexico | -8847.323 | 560.074 | -15.797 | *<.001* | <.001 |
| Belgium-Germany | -74.96 | 761.204 | -0.098 | *0.922* | 1.000 |
| Belgium-Spain | -83.371 | 786.133 | -0.106 | *0.916* | 1.000 |
| Belgium-Chile | -428.045 | 866.378 | -0.494 | *0.621* | 1.000 |
| Belgium-South Korea | -451.507 | 835.268 | -0.541 | *0.589* | 1.000 |
| Belgium-Italy | -1911.598 | 783.834 | -2.439 | *0.015* | 1.000 |
| Belgium-Croatia | -2110.198 | 784.327 | -2.69 | *0.007* | 1.000 |
| Belgium-Austria | 2509.879 | 949.703 | 2.643 | *0.008* | 1.000 |
| Belgium-Portugal | -2710.607 | 788.674 | -3.437 | *<.001* | 0.191 |
| Belgium-Poland | -2868.035 | 717.913 | -3.995 | *<.001* | 0.021 |
| Belgium-Israel | -2915.936 | 848.083 | -3.438 | *<.001* | 0.190 |
| Belgium-Switzerland | -3050.446 | 869.34 | -3.509 | *<.001* | 0.146 |
| Belgium-France | -3352.758 | 816.64 | -4.106 | *<.001* | 0.013 |
| Belgium-South Africa | -3430.754 | 807.818 | -4.247 | *<.001* | 0.007 |
| Belgium-Peru | -4379.459 | 775.9 | -5.644 | *<.001* | <.001 |
| Belgium-United Kingdom | -6083.909 | 840.106 | -7.242 | *<.001* | <.001 |
| Belgium-Australia | 6103.1 | 986.874 | 6.184 | *<.001* | <.001 |
| Belgium-Canada | -6889.455 | 778.908 | -8.845 | *<.001* | <.001 |
| Belgium-United States of America | -6978.449 | 783.485 | -8.907 | *<.001* | <.001 |
| Belgium-Ireland | -7461.632 | 817.909 | -9.123 | *<.001* | <.001 |
| Belgium-New Zealand | -7544.449 | 771.233 | -9.782 | *<.001* | <.001 |
| Belgium-Mexico | -8839.357 | 794.915 | -11.12 | *<.001* | <.001 |
| Germany-Spain | -8.411 | 479.199 | -0.018 | *0.986* | 1.000 |
| Germany-Chile | -353.085 | 601.861 | -0.587 | *0.557* | 1.000 |
| Germany-South Korea | -376.547 | 556.147 | -0.677 | *0.498* | 1.000 |
| Germany-Italy | -1836.638 | 475.417 | -3.863 | *<.001* | 0.036 |
| Germany-Croatia | 2035.238 | 476.229 | 4.274 | *<.001* | 0.006 |
| Germany-Austria | 2434.92 | 716.633 | 3.398 | *<.001* | 0.221 |
| Germany-Portugal | -2635.648 | 483.355 | -5.453 | *<.001* | <.001 |
| Germany-Poland | -2793.075 | 356.406 | -7.837 | *<.001* | <.001 |
| Germany-Israel | -2840.976 | 575.214 | -4.939 | *<.001* | <.001 |
| Germany-Switzerland | -2975.486 | 606.118 | -4.909 | *<.001* | <.001 |
| Germany-France | 3277.798 | 527.756 | -0.376.211 | *<.001* | <.001 |
| Germany-South Africa | -3355.795 | 514.001 | -6.529 | *<.001* | <.001 |
| Germany-Peru | -4304.5 | 462.221 | -9.313 | *<.001* | <.001 |
| Germany-United Kingdom | -6008.949 | 563.386 | -10.666 | *<.001* | <.001 |
| Germany-Australia | 6028.141 | 765.21 | 7.878 | *<.001* | <.001 |
| Germany-Canada | 6814.495 | 467.252 | 14.584 | *<.001* | <.001 |
| Germany-United States of America | -6903.489 | 474.842 | -14.539 | *<.001* | <.001 |
| Germany-Ireland | -7386.672 | 529.718 | -13.945 | *<.001* | <.001 |
| Germany-New Zealand | -7469.489 | 454.342 | -16.44 | *<.001* | <.001 |
| Germany-Mexico | -8764.397 | 493.473 | -17.761 | *<.001* | <.001 |
| Spain-Chile | -344.674 | 633.097 | -0.544 | *0.586* | 1.000 |
| Spain-South Korea | 368.136 | 589.809 | 0.624 | *0.533* | 1.000 |
| Spain-Italy | 1828.227 | 514.389 | 3.554 | *<.001* | 0.123 |
| Spain-Croatia | 2026.827 | 515.14 | 3.935 | *<.001* | 0.027 |
| Spain-Austria | 2426.508 | 743.06 | 3.266 | *0.001* | 0.355 |
| Spain-Portugal | 2627.236 | 521.735 | 5.036 | *<.001* | <.001 |
| Spain-Poland | 2784.664 | 406.94 | 6.843 | *<.001* | <.001 |
| Spain-Israel | 2832.565 | 607.821 | 4.66 | *<.001* | 0.001 |
| Spain-Switzerland | -2967.075 | 637.145 | -4.657 | *<.001* | 0.001 |
| Spain-France | 3269.387 | 563.117 | 5.806 | *<.001* | <.001 |
| Spain-South Africa | 3347.383 | 550.247 | 6.083 | *<.001* | <.001 |
| Spain-Peru | 4296.088 | 502.218 | 8.554 | *<.001* | <.001 |
| Spain-United Kingdom | -6000.538 | 596.639 | -10.057 | *<.001* | <.001 |
| Spain-Australia | 6019.729 | 790.014 | 7.62 | *<.001* | <.001 |
| Spain-Canada | 6806.083 | 506.852 | 13.428 | *<.001* | <.001 |
| Spain-United States of America | -6895.078 | 513.858 | -13.418 | *<.001* | <.001 |
| Spain-Ireland | 7378.261 | 564.957 | 13.06 | *<.001* | <.001 |
| Spain-New Zealand | 7461.078 | 494.976 | 15.074 | *<.001* | <.001 |
| Spain-Mexico | 8755.986 | 531.122 | 16.486 | *<.001* | <.001 |
| Chile-South Korea | 23.462 | 693.166 | 0.034 | *0.973* | 1.000 |
| Chile-Italy | 1483.553 | 630.239 | 2.354 | *0.019* | 1.000 |
| Chile-Croatia | 1682.153 | 630.852 | 2.666 | *0.008* | 1.000 |
| Chile-Austria | 2081.834 | 827.491 | 2.516 | *0.012* | 1.000 |
| Chile-Portugal | 2282.562 | 636.248 | 3.588 | *<.001* | 0.108 |
| Chile-Poland | 2439.99 | 546.081 | 4.468 | *<.001* | 0.003 |
| Chile-Israel | 2487.891 | 708.555 | 3.511 | *<.001* | 0.145 |
| Chile-Switzerland | 2622.401 | 733.865 | 3.573 | *<.001* | 0.115 |
| Chile-France | 2924.712 | 670.601 | 4.361 | *<.001* | 0.004 |
| Chile-South Africa | 3002.709 | 659.831 | 4.551 | *<.001* | 0.002 |
| Chile-Peru | 3951.414 | 620.344 | 6.37 | *<.001* | <.001 |
| Chile-United Kingdom | 5655.864 | 698.987 | 8.092 | *<.001* | <.001 |
| Chile-Australia | 5675.055 | 869.9 | 6.524 | *<.001* | <.001 |
| Chile-Canada | 6461.409 | 624.102 | 10.353 | *<.001* | <.001 |
| Chile-United States of America | 6550.404 | 629.805 | 10.401 | *<.001* | <.001 |
| Chile-Ireland | 7033.587 | 672.146 | 10.464 | *<.001* | <.001 |
| Chile-New Zealand | 7116.404 | 614.496 | 11.581 | *<.001* | <.001 |
| Chile-Mexico | 8411.312 | 643.968 | 13.062 | *<.001* | <.001 |
| South Korea-Italy | 1460.091 | 586.74 | 2.488 | *0.013* | 1.000 |
| South Korea-Croatia | 1658.691 | 587.398 | 2.824 | *0.005* | 1.000 |
| South Korea-Austria | 2058.373 | 794.862 | 2.59 | *0.01* | 1.000 |
| South Korea-Portugal | 2259.101 | 593.19 | 3.808 | *<.001* | 0.045 |
| South Korea-Poland | 2416.528 | 495.245 | 4.879 | *<.001* | <.001 |
| South Korea-Israel | 2464.429 | 670.159 | 3.677 | *<.001* | 0.077 |
| South Korea-Switzerland | -2598.939 | 696.865 | -3.729 | *<.001* | 0.062 |
| South Korea-France | 2901.251 | 629.896 | 4.606 | *<.001* | 0.001 |
| South Korea-South Africa | 2979.248 | 618.417 | 4.818 | *<.001* | <.001 |
| South Korea-Peru | 3927.953 | 576.099 | 6.818 | *<.001* | <.001 |
| South Korea-United Kingdom | -5632.402 | 660.035 | -8.533 | *<.001* | <.001 |
| South Korea-Australia | 5651.594 | 838.921 | 6.737 | *<.001* | <.001 |
| South Korea-Canada | 6437.948 | 580.143 | 11.097 | *<.001* | <.001 |
| South Korea-United States of America | -6526.942 | 586.274 | -11.133 | *<.001* | <.001 |
| South Korea-Ireland | 7010.125 | 631.54 | 11.1 | *<.001* | <.001 |
| South Korea-New Zealand | 7092.942 | 569.797 | 12.448 | *<.001* | <.001 |
| South Korea-Mexico | 8387.85 | 601.463 | 13.946 | *<.001* | <.001 |
| Italy-Croatia | 198.601 | 511.624 | 0.388 | *0.698* | 1.000 |
| Italy-Austria | 598.282 | 740.626 | 0.808 | *0.419* | 1.000 |
| Italy-Portugal | -799.01 | 518.263 | -1.542 | *0.123* | 1.000 |
| Italy-Poland | -956.437 | 402.479 | -2.376 | *0.017* | 1.000 |
| Italy-Israel | 1004.338 | 604.843 | 1.66 | *0.097* | 1.000 |
| Italy-Switzerland | -1138.848 | 634.305 | -1.795 | *0.073* | 1.000 |
| Italy-France | 1441.16 | 559.902 | 2.574 | *0.01* | 1.000 |
| Italy-South Africa | -1519.157 | 546.956 | -2.777 | *0.005* | 1.000 |
| Italy-Peru | -2467.862 | 498.61 | -4.949 | *<.001* | <.001 |
| Italy-United Kingdom | -4172.311 | 593.606 | -7.029 | *<.001* | <.001 |
| Italy-Australia | 4191.503 | 787.725 | 5.321 | *<.001* | <.001 |
| Italy-Canada | 4977.857 | 503.277 | 9.891 | *<.001* | <.001 |
| Italy-United States of America | -5066.852 | 510.332 | -9.929 | *<.001* | <.001 |
| Italy-Ireland | 5550.035 | 561.752 | 9.88 | *<.001* | <.001 |
| Italy-New Zealand | -5632.851 | 491.315 | -11.465 | *<.001* | <.001 |
| Italy-Mexico | -6927.759 | 527.712 | -13.128 | *<.001* | <.001 |
| Croatia-Austria | 399.681 | 741.148 | 0.539 | *0.59* | 1.000 |
| Croatia-Portugal | -600.409 | 519.008 | -1.157 | *0.247* | 1.000 |
| Croatia-Poland | -757.837 | 403.439 | -1.878 | *0.06* | 1.000 |
| Croatia-Israel | -805.737 | 605.482 | -1.331 | *0.183* | 1.000 |
| Croatia-Switzerland | -940.248 | 634.914 | -1.481 | *0.139* | 1.000 |
| Croatia-France | -1242.559 | 560.592 | -2.217 | *0.027* | 1.000 |
| Croatia-South Africa | -1320.556 | 547.663 | -2.411 | *0.016* | 1.000 |
| Croatia-Peru | -2269.261 | 499.385 | -4.544 | *<.001* | 0.002 |
| Croatia-United Kingdom | -3973.711 | 594.257 | -6.687 | *<.001* | <.001 |
| Croatia-Australia | 3992.902 | 788.216 | 5.066 | *<.001* | <.001 |
| Croatia-Canada | 4779.256 | 504.045 | 9.482 | *<.001* | <.001 |
| Croatia-United States of America | -4868.251 | 511.089 | -9.525 | *<.001* | <.001 |
| Croatia-Ireland | -5351.434 | 562.44 | -9.515 | *<.001* | <.001 |
| Croatia-New Zealand | -5434.251 | 492.101 | -11.043 | *<.001* | <.001 |
| Croatia-Mexico | -6729.159 | 528.444 | -12.734 | *<.001* | <.001 |
| Austria-Portugal | -200.728 | 745.747 | -0.269 | *0.788* | 1.000 |
| Austria-Poland | -358.156 | 670.471 | -0.534 | *0.593* | 1.000 |
| Austria-Israel | -406.056 | 808.317 | -0.502 | *0.615* | 1.000 |
| Austria-Switzerland | -540.567 | 830.593 | -0.651 | *0.515* | 1.000 |
| Austria-France | -842.878 | 775.263 | -1.087 | *0.277* | 1.000 |
| Austria-South Africa | -920.875 | 765.965 | -1.202 | *0.229* | 1.000 |
| Austria-Peru | -1869.58 | 732.225 | -2.553 | *0.011* | 1.000 |
| Austria-United Kingdom | -3574.03 | 799.943 | -4.468 | *<.001* | 0.003 |
| Austria-Australia | 3593.221 | 952.918 | 3.771 | *<.001* | 0.053 |
| Austria-Canada | -4379.575 | 735.411 | -5.955 | *<.001* | <.001 |
| Austria-United States of America | -4468.57 | 740.257 | -6.037 | *<.001* | <.001 |
| Austria-Ireland | -4951.753 | 776.599 | -6.376 | *<.001* | <.001 |
| Austria-New Zealand | -5034.57 | 727.277 | -6.922 | *<.001* | <.001 |
| Austria-Mexico | -6329.477 | 752.344 | -8.413 | *<.001* | <.001 |
| Portugal-Poland | 157.428 | 411.826 | 0.382 | *0.702* | 1.000 |
| Portugal-Israel | 205.328 | 611.103 | 0.336 | *0.737* | 1.000 |
| Portugal-Switzerland | -339.839 | 640.277 | -0.531 | *0.596* | 1.000 |
| Portugal-France | 642.15 | 566.658 | 1.133 | *0.257* | 1.000 |
| Portugal-South Africa | -720.147 | 553.87 | -1.3 | *0.194* | 1.000 |
| Portugal-Peru | 1668.852 | 506.185 | 3.297 | *<.001* | 0.318 |
| Portugal-United Kingdom | -3373.302 | 599.982 | -5.622 | *<.001* | <.001 |
| Portugal-Australia | 3392.493 | 792.541 | 4.281 | *<.001* | 0.006 |
| Portugal-Canada | 4178.847 | 510.783 | 8.181 | *<.001* | <.001 |
| Portugal-United States of America | -4267.842 | 517.735 | -8.243 | *<.001* | <.001 |
| Portugal-Ireland | 4751.025 | 568.486 | 8.357 | *<.001* | <.001 |
| Portugal-New Zealand | 4833.842 | 499.001 | 9.687 | *<.001* | <.001 |
| Portugal-Mexico | 6128.749 | 534.875 | 11.458 | *<.001* | <.001 |
| Poland-Israel | 47.901 | 516.565 | 0.093 | *0.926* | 1.000 |
| Poland-Switzerland | -182.411 | 550.77 | -0.331 | *0.74* | 1.000 |
| Poland-France | 484.723 | 463.136 | 1.047 | *0.295* | 1.000 |
| Poland-South Africa | -562.719 | 447.398 | -1.258 | *0.208* | 1.000 |
| Poland-Peru | 1511.424 | 386.803 | 3.907 | *<.001* | 0.030 |
| Poland-United Kingdom | -3215.874 | 503.361 | -6.389 | *<.001* | <.001 |
| Poland-Australia | 3235.065 | 722.16 | 4.48 | *<.001* | 0.002 |
| Poland-Canada | 4021.42 | 392.801 | 10.238 | *<.001* | <.001 |
| Poland-United States of America | -4110.414 | 401.8 | -10.23 | *<.001* | <.001 |
| Poland-Ireland | 4593.597 | 465.37 | 9.871 | *<.001* | <.001 |
| Poland-New Zealand | 4676.414 | 377.352 | 12.393 | *<.001* | <.001 |
| Poland-Mexico | 5971.322 | 423.656 | 14.095 | *<.001* | <.001 |
| Israel-Switzerland | -134.51 | 712.175 | -0.189 | *0.85* | 1.000 |
| Israel-France | 436.822 | 646.792 | 0.675 | *0.499* | 1.000 |
| Israel-South Africa | -514.819 | 635.619 | -0.81 | *0.418* | 1.000 |
| Israel-Peru | -1463.524 | 594.526 | -2.462 | *0.014* | 1.000 |
| Israel-United Kingdom | -3167.973 | 676.179 | -4.685 | *<.001* | 0.001 |
| Israel-Australia | 3187.165 | 851.681 | 3.742 | *<.001* | 0.059 |
| Israel-Canada | 3973.519 | 598.446 | 6.64 | *<.001* | <.001 |
| Israel-United States of America | -4062.514 | 604.391 | -6.722 | *<.001* | <.001 |
| Israel-Ireland | 4545.697 | 648.394 | 7.011 | *<.001* | <.001 |
| Israel-New Zealand | -4628.513 | 588.422 | -7.866 | *<.001* | <.001 |
| Israel-Mexico | -5923.421 | 619.136 | -9.567 | *<.001* | <.001 |
| Switzerland-France | 302.312 | 674.424 | 0.448 | *0.654* | 1.000 |
| Switzerland-South Africa | 380.308 | 663.716 | 0.573 | *0.567* | 1.000 |
| Switzerland-Peru | 1329.013 | 624.476 | 2.128 | *0.033* | 1.000 |
| Switzerland-United Kingdom | -3033.463 | 702.656 | -4.317 | *<.001* | 0.005 |
| Switzerland-Australia | 3052.654 | 872.851 | 3.497 | *<.001* | 0.153 |
| Switzerland-Canada | 3839.008 | 628.208 | 6.111 | *<.001* | <.001 |
| Switzerland-United States of America | -3928.003 | 633.874 | -6.197 | *<.001* | <.001 |
| Switzerland-Ireland | 4411.186 | 675.961 | 6.526 | *<.001* | <.001 |
| Switzerland-New Zealand | 4494.003 | 618.667 | 7.264 | *<.001* | <.001 |
| Switzerland-Mexico | 5788.911 | 647.949 | 8.934 | *<.001* | <.001 |
| France-South Africa | -77.997 | 593.015 | -0.132 | *0.895* | 1.000 |
| France-Peru | -1026.702 | 548.741 | -1.871 | *0.061* | 1.000 |
| France-United Kingdom | -2731.152 | 636.296 | -4.292 | *<.001* | 0.006 |
| France-Australia | 2750.343 | 820.375 | 3.353 | *<.001* | 0.260 |
| France-Canada | 3536.697 | 552.986 | 6.396 | *<.001* | <.001 |
| France-United States of America | -3625.692 | 559.414 | -6.481 | *<.001* | <.001 |
| France-Ireland | -4108.875 | 606.688 | -6.773 | *<.001* | <.001 |
| France-New Zealand | -4191.691 | 542.121 | -7.732 | *<.001* | <.001 |
| France-Mexico | -5486.599 | 575.313 | -9.537 | *<.001* | <.001 |
| South Africa-Peru | 948.705 | 535.526 | 1.772 | *0.076* | 1.000 |
| South Africa-United Kingdom | -2653.155 | 624.935 | -4.245 | *<.001* | 0.007 |
| South Africa-Australia | 2672.346 | 811.595 | 3.293 | *<.001* | 0.322 |
| South Africa-Canada | 3458.7 | 539.874 | 6.406 | *<.001* | <.001 |
| South Africa-United States of America | -3547.695 | 546.456 | -6.492 | *<.001* | <.001 |
| South Africa-Ireland | 4030.878 | 594.761 | 6.777 | *<.001* | <.001 |
| South Africa-New Zealand | 4113.695 | 528.74 | 7.78 | *<.001* | <.001 |
| South Africa-Mexico | 5408.602 | 562.722 | 9.612 | *<.001* | <.001 |
| Peru-United Kingdom | -1704.45 | 583.09 | -2.923 | *0.003* | 1.000 |
| Peru-Australia | 1723.641 | 779.832 | 2.21 | *0.027* | 1.000 |
| Peru-Canada | 2509.995 | 490.831 | 5.114 | *<.001* | <.001 |
| Peru-United States of America | -2598.99 | 498.062 | -5.218 | *<.001* | <.001 |
| Peru-Ireland | 3082.173 | 550.628 | 5.598 | *<.001* | <.001 |
| Peru-New Zealand | 3164.99 | 478.557 | 6.614 | *<.001* | <.001 |
| Peru-Mexico | 4459.897 | 515.855 | 8.646 | *<.001* | <.001 |
| United Kingdom-Australia | 19.191 | 843.737 | 0.023 | *0.982* | 1.000 |
| United Kingdom-Canada | 805.545 | 587.086 | 1.372 | *0.17* | 1.000 |
| United Kingdom-United States of America | -894.54 | 593.145 | -1.508 | *0.132* | 1.000 |
| United Kingdom-Ireland | 1377.723 | 637.924 | 2.16 | *0.031* | 1.000 |
| United Kingdom-New Zealand | 1460.54 | 576.865 | 2.532 | *0.011* | 1.000 |
| United Kingdom-Mexico | 2755.448 | 608.163 | 4.531 | *<.001* | 0.002 |
| Australia-Canada | -786.354 | 782.824 | -1.005 | *0.315* | 1.000 |
| Australia-United States of America | -875.349 | 787.378 | -1.112 | *0.266* | 1.000 |
| Australia-Ireland | -1358.532 | 821.639 | -1.653 | *0.098* | 1.000 |
| Australia-New Zealand | -1441.349 | 775.188 | -1.859 | *0.063* | 1.000 |
| Australia-Mexico | -2736.256 | 798.752 | -3.426 | *<.001* | 0.199 |
| Canada-United States of America | -88.995 | 502.734 | -0.177 | *0.859* | 1.000 |
| Canada-Ireland | -572.178 | 554.858 | -1.031 | *0.302* | 1.000 |
| Canada-New Zealand | -654.994 | 483.418 | -1.355 | *0.175* | 1.000 |
| Canada-Mexico | -1949.902 | 520.368 | -3.747 | *<.001* | 0.058 |
| United States of America-Ireland | 483.183 | 561.265 | 0.861 | *0.389* | 1.000 |
| United States of America-New Zealand | 566 | 490.759 | 1.153 | *0.249* | 1.000 |
| United States of America-Mexico | 1860.908 | 527.194 | 3.53 | *<.001* | 0.135 |
| Ireland-New Zealand | -82.817 | 544.031 | -0.152 | *0.879* | 1.000 |
| Ireland-Mexico | -1377.725 | 577.113 | -2.387 | *0.017* | 1.000 |
| New Zealand-Mexico | 1294.908 | 508.808 | 2.545 | *0.011* | 1.000 |

*Note*. a = Significance values have been adjusted by the Bonferroni correction for multiple tests.
