# Supplementary material for: The moderating role of impulsivity in the association between sexual abuse and substance use frequency across countries, gender identities, and sexual orientations
Source: PLoS One. 2026 Sep 30;21(9):e0355986. doi: 10.1371/journal.pone.0355986 (PMC13626376; doi:10.1371/journal.pone.0355986)
Supplement: Supplementary file 3 [file pone.0355986.s003.docx]

*Table A. Latent Variable Interaction and Simple Slopes Analysis for Lack of Premeditation Moderating the Relationship Between CSA and ASSIST Scores*

| Predictor | B | *β* | SE | z | p |
| --- | --- | --- | --- | --- | --- |
| CSA | -0.08 | -0.01 | 0.15 | -0.50 | .617 |
| UPPS-PR | 0.24 | 0.16 | 0.01 | 35.72 | <.001 |
| CSA x UPPS-PR | -0.04 | -0.03 | 0.02 | -2.11 | .035 |

*Note*. CSA = Childhood Sexual Abuse; UPPS_PR = Lack of Premeditation sub-facet of the SUPPS-P scale; SE = Standard Error.

*Table B. Simple Slopes Analysis for the Interaction Between CSA and Lack of Premeditation on ASSIST Scores*

| Level of UPPS_PR | B | SE | p |
| --- | --- | --- | --- |
| Low (-1 *SD*) | -0.29 | 0.06 | <.001 |
| Mean | -0.39 | 0.04 | <.001 |
| High (+1 *SD*) | -0.48 | 0.06 | <.001 |

*Note*. UPPS_PR = Lack of Premeditation sub-facet of the SUPPS-P scale; SE = Standard Error.

*Table C. Latent Variable Interaction and Simple Slopes Analysis for Lack of Perseverance Moderating the Relationship Between CSA and ASSIST Scores*

| Predictor | B | *β* | SE | z | p |
| --- | --- | --- | --- | --- | --- |
| CSA | 0.07 | 0.01 | 0.14 | 0.46 | .642 |
| UPPS-PE | 0.13 | 0.10 | 0.01 | 21.93 | <.001 |
| CSA x UPPS-PE | -0.06 | -0.05 | 0.02 | -3.41 | .001 |

*Note*. CSA = Childhood Sexual Abuse; UPPS_PE = Lack of Perseverance sub-facet of the SUPPS-P scale; SE = Standard Error.

*Table D. Simple Slopes Analysis for the Interaction Between CSA and Lack of Perseverance on ASSIST Scores*

| Level of UPPS_PE | B | SE | p |
| --- | --- | --- | --- |
| Low (-1 *SD*) | -0.25 | 0.06 | <.001 |
| Mean | -0.40 | 0.04 | <.001 |
| High (+1 *SD*) | -0.55 | 0.06 | <.001 |

*Note*. UPPS_PE = Lack of Perseverance sub-facet of the SUPPS-P scale; SE = Standard Error.

*Table E. Latent Variable Interaction and Simple Slopes Analysis for Negative Urgency Moderating the Relationship Between CSA and ASSIST Scores*

| Predictor | B | *β* | SE | z | p |
| --- | --- | --- | --- | --- | --- |
| CSA | 0.04 | 0.004 | 0.15 | 0.33 | .742 |
| UPPS-NU | 0.12 | 0.10 | 0.01 | 23.55 | <.001 |
| CSA x UPPS-NU | -0.05 | -0.04 | 0.02 | -3.15 | .002 |

*Note*. CSA = Childhood Sexual Abuse; UPPS_NU = Lack of Perseverance sub-facet of the SUPPS-P scale; SE = Standard Error.

*Table F. Simple Slopes Analysis for the Interaction Between CSA and Negative Urgency on ASSIST Scores*

| Level of UPPS_NU | B | SE | p |
| --- | --- | --- | --- |
| Low (-1 *SD*) | -0.26 | 0.06 | <.001 |
| Mean | -0.40 | 0.04 | <.001 |
| High (+1 *SD*) | -0.54 | 0.06 | <.001 |

*Note*. UPPS_NU = Negative Urgency sub-facet of the SUPPS-P scale; SE = Standard Error.

*Table G. Latent Variable Interaction and Simple Slopes Analysis for Sensation Seeking Moderating the Relationship Between CSA and ASSIST Scores*

| Predictor | B | *β* | SE | z | p |
| --- | --- | --- | --- | --- | --- |
| CSA | 0.10 | 0.10 | 0.16 | 0.64 | .523 |
| UPPS-SE | 0.26 | 0.21 | 0.01 | 49.44 | <.001 |
| CSA x UPPS-SE | -0.04 | -0.04 | 0.02 | -2.76 | .006 |

*Note*. CSA = Childhood Sexual Abuse; UPPS_SE = Sensation Seeking sub-facet of the SUPPS-P scale; SE = Standard Error.

*Table H. Simple Slopes Analysis for the Interaction Between CSA and Sensation Seeking on ASSIST Scores*

| Level of UPPS_SE | B | SE | p |
| --- | --- | --- | --- |
| Low (-1 *SD*) | -0.21 | 0.06 | <.001 |
| Mean | -0.33 | 0.04 | <.001 |
| High (+1 *SD*) | -0.45 | 0.06 | <.001 |

*Note*. UPPS_SE = Sensation Seeking sub-facet of the SUPPS-P scale; SE = Standard Error.

*Table I. Latent Variable Interaction and Simple Slopes Analysis for Positive Urgency Moderating the Relationship Between CSA and ASSIST Scores*

| Predictor | B | *β* | SE | z | p |
| --- | --- | --- | --- | --- | --- |
| CSA | 0.02 | 0.001 | 0.17 | 0.09 | .921 |
| UPPS-PU | 0.23 | 0.18 | 0.01 | 40.42 | <.001 |
| CSA x UPPS-PU | -0.04 | -0.04 | 0.02 | -2.43 | .015 |

*Note*. CSA = Childhood Sexual Abuse; UPPS_PU = Positive Urgency sub-facet of the SUPPS-P scale; SE = Standard Error.

*Table J. Simple Slopes Analysis for the Interaction Between CSA and Sensation Seeking on ASSIST Scores*

| Level of UPPS_PU | B | SE | p |
| --- | --- | --- | --- |
| Low (-1 *SD*) | -0.27 | 0.06 | <.001 |
| Mean | -0.38 | 0.04 | <.001 |
| High (+1 *SD*) | -0.48 | 0.06 | <.001 |

*Note*. UPPS_PU = Positive Urgency sub-facet of the SUPPS-P scale; SE = Standard Error.

*Table K. Latent Variable Interaction and Simple Slopes Analysis for Lack of Premeditation Moderating the Relationship Between AASA and ASSIST Scores*

| Predictor | B | *β* | SE | z | p |
| --- | --- | --- | --- | --- | --- |
| AASA | 0.49 | 0.06 | 0.11 | 4.56 | <.001 |
| UPPS-PR | 0.23 | 0.15 | 0.01 | 30.55 | <.001 |
| AASA x UPPS-PR | 0.01 | 0.01 | 0.01 | 0.84 | .399 |

*Note*. AASA = Adult Adolescent Sexual Abuse; UPPS_PR = Lack of Premeditation sub-facet of the SUPPS-P scale; SE = Standard Error.

*Table L. Simple Slopes Analysis for the Interaction Between AASA and Lack of Premeditation on ASSIST Scores*

| Level of UPPS_PR | B | SE | p |
| --- | --- | --- | --- |
| Low (-1 *SD*) | 0.55 | 0.04 | <.001 |
| Mean | 0.58 | 0.03 | <.001 |
| High (+1 *SD*) | 0.60 | 0.04 | <.001 |

*Note*. UPPS_PR = Lack of Premeditation sub-facet of the SUPPS-P scale; SE = Standard Error.

*Table M. Latent Variable Interaction and Simple Slopes Analysis for Lack of Perseverance Moderating the Relationship Between AASA and ASSIST Scores*

| Predictor | B | *β* | SE | z | p |
| --- | --- | --- | --- | --- | --- |
| AASA | 0.44 | 0.06 | 0.10 | 4.28 | <.001 |
| UPPS-PE | 0.11 | 0.08 | 0.01 | 17.50 | <.001 |
| AASA x UPPS-PE | 0.02 | 0.02 | 0.01 | 1.66 | .097 |

*Note*. AASA = Adult Adolescent Sexual Abuse; UPPS_PE = Lack of Perseverance sub-facet of the SUPPS-P scale; SE = Standard Error.

*Table N. Simple Slopes Analysis for the Interaction Between AASA and Lack of Perseverance on ASSIST Scores*

| Level of UPPS_PE | B | SE | p |
| --- | --- | --- | --- |
| Low (-1 *SD*) | 0.55 | 0.05 | <.001 |
| Mean | 0.60 | 0.03 | <.001 |
| High (+1 *SD*) | 0.65 | 0.04 | <.001 |

*Note*. UPPS_PE = Lack of Perseverance sub-facet of the SUPPS-P scale; SE = Standard Error.

*Table O. Latent Variable Interaction and Simple Slopes Analysis for Negative Urgency Moderating the Relationship Between AASA and ASSIST Scores*

| Predictor | B | *β* | SE | z | p |
| --- | --- | --- | --- | --- | --- |
| AASA | 0.34 | 0.04 | 0.11 | 3.18 | .001 |
| UPPS-NU | 0.11 | 0.09 | 0.01 | 18.30 | <.001 |
| AASA x UPPS-NU | 0.03 | 0.04 | 0.01 | 2.44 | .015 |

*Note*. AASA = Adult Adolescent Sexual Abuse; UPPS_NU = Negative Urgency sub-facet of the SUPPS-P scale; SE = Standard Error.

*Table P. Simple Slopes Analysis for the Interaction Between AASA and Negative Urgency on ASSIST Scores*

| Level of UPPS_NU | B | SE | p |
| --- | --- | --- | --- |
| Low (-1 *SD*) | 0.51 | 0.05 | <.001 |
| Mean | 0.58 | 0.03 | <.001 |
| High (+1 *SD*) | 0.66 | 0.04 | <.001 |

*Note*. UPPS_NU = Negative Urgency sub-facet of the SUPPS-P scale; SE = Standard Error.

*Table Q. Latent Variable Interaction and Simple Slopes Analysis for Sensation Seeking Moderating the Relationship Between AASA and ASSIST Scores*

| Predictor | B | *β* | SE | z | p |
| --- | --- | --- | --- | --- | --- |
| AASA | 0.41 | 0.05 | 0.12 | 3.49 | <.001 |
| UPPS-SE | 0.25 | 0.20 | 0.01 | 42.92 | <.001 |
| AASA x UPPS-SE | 0.01 | 0.02 | 0.01 | 1.26 | .208 |

*Note*. AASA = Adult Adolescent Sexual Abuse; UPPS_SE = Sensation Seeking sub-facet of the SUPPS-P scale; SE = Standard Error.

*Table R. Simple Slopes Analysis for the Interaction Between AASA and Sensation Seeking on ASSIST Scores*

| Level of UPPS_SE | B | SE | p |
| --- | --- | --- | --- |
| Low (-1 *SD*) | 0.51 | 0.04 | <.001 |
| Mean | 0.55 | 0.03 | <.001 |
| High (+1 *SD*) | 0.59 | 0.04 | <.001 |

*Note*. UPPS_SE = Sensation Seeking sub-facet of the SUPPS-P scale; SE = Standard Error.

*Table S. Latent Variable Interaction and Simple Slopes Analysis for Positive Urgency Moderating the Relationship Between AASA and ASSIST Scores*

| Predictor | B | *β* | SE | z | p |
| --- | --- | --- | --- | --- | --- |
| AASA | 0.38 | 0.05 | 0.12 | 3.16 | .002 |
| UPPS-PU | 0.22 | 0.17 | 0.01 | 34.48 | <.001 |
| AASA x UPPS-PU | 0.02 | 0.02 | 0.01 | 1.49 | .135 |

*Note*. AASA = Adult Adolescent Sexual Abuse; UPPS_PU = Positive Urgency sub-facet of the SUPPS-P scale; SE = Standard Error.

*Table T. Simple Slopes Analysis for the Interaction Between AASA and Sensation Seeking on ASSIST Scores*

| Level of UPPS_PU | B | SE | p |
| --- | --- | --- | --- |
| Low (-1 *SD*) | 0.51 | 0.05 | <.001 |
| Mean | 0.55 | 0.03 | <.001 |
| High (+1 *SD*) | 0.60 | 0.04 | <.001 |

*Note*. UPPS_PU = Positive Urgency sub-facet of the SUPPS-P scale; SE = Standard Error.

*Table U. Latent Variable Interaction and Simple Slopes Analysis for Lack of Premeditation Moderating the Relationship Between CSA + AASA and ASSIST Scores*

| Predictor | B | *β* | SE | z | p |
| --- | --- | --- | --- | --- | --- |
| CSA + AASA | 0.16 | 0.02 | 0.12 | 1.29 | .196 |
| UPPS-PR | 0.22 | 0.14 | 0.01 | 30.20 | <.001 |
| CSA + AASA x UPPS-PR | 0.07 | 0.06 | 0.02 | 4.51 | <.001 |

*Note*. CSA + AASA = Lifetime Sexual Abuse; UPPS_PR = Lack of Premeditation sub-facet of the SUPPS-P scale; SE = Standard Error.

*Table V. Simple Slopes Analysis for the Interaction Between CSA + AASA and Lack of Premeditation on ASSIST Scores*

| Level of UPPS_PR | B | SE | p |
| --- | --- | --- | --- |
| Low (-1 *SD*) | 0.51 | 0.05 | <.001 |
| Mean | 0.67 | 0.04 | <.001 |
| High (+1 *SD*) | 0.82 | 0.05 | <.001 |

*Note*. UPPS_PR = Lack of Premeditation sub-facet of the SUPPS-P scale; SE = Standard Error.

*Table W. Latent Variable Interaction and Simple Slopes Analysis for Lack of Perseverance Moderating the Relationship Between CSA + AASA and ASSIST Scores*

| Predictor | B | *β* | SE | z | p |
| --- | --- | --- | --- | --- | --- |
| CSA + AASA | 0.19 | 0.02 | 0.12 | 1.61 | .107 |
| UPPS-PE | 0.11 | 0.08 | 0.01 | 16.56 | <.001 |
| CSA + AASA x UPPS-PE | 0.07 | 0.07 | 0.02 | 4.76 | <.001 |

*Note*. CSA + AASA = Lifetime Sexual Abuse; UPPS_PE = Lack of Perseverance sub-facet of the SUPPS-P scale; SE = Standard Error.

*Table X. Simple Slopes Analysis for the Interaction Between CSA + AASA and Lack of Perseverance on ASSIST Scores*

| Level of UPPS_PE | B | SE | p |
| --- | --- | --- | --- |
| Low (-1 *SD*) | 0.53 | 0.05 | <.001 |
| Mean | 0.70 | 0.04 | <.001 |
| High (+1 *SD*) | 0.86 | 0.05 | <.001 |

*Note*. UPPS_PE = Lack of Perseverance sub-facet of the SUPPS-P scale; SE = Standard Error.

*Table Y. Latent Variable Interaction and Simple Slopes Analysis for Negative Urgency Moderating the Relationship Between CSA + AASA and ASSIST Scores*

| Predictor | B | *β* | SE | z | p |
| --- | --- | --- | --- | --- | --- |
| CSA + AASA | 0.10 | 0.01 | 0.12 | 0.85 | .395 |
| UPPS-NU | 0.10 | 0.08 | 0.01 | 17.89 | <.001 |
| CSA + AASA x UPPS-NU | 0.06 | 0.07 | 0.02 | 5.08 | <.001 |

*Note*. CSA + AASA = Lifetime Sexual Abuse; UPPS_NU = Negative Urgency sub-facet of the SUPPS-P scale; SE = Standard Error.

*Table Z. Simple Slopes Analysis for the Interaction Between CSA + AASA and Negative Urgency on ASSIST Scores*

| Level of UPPS_NU | B | SE | p |
| --- | --- | --- | --- |
| Low (-1 *SD*) | 0.50 | 0.05 | <.001 |
| Mean | 0.68 | 0.04 | <.001 |
| High (+1 *SD*) | 0.86 | 0.05 | <.001 |

*Note*. UPPS_NU = Negative Urgency sub-facet of the SUPPS-P scale; SE = Standard Error.

*Table AA. Latent Variable Interaction and Simple Slopes Analysis for Sensation Seeking Moderating the Relationship Between CSA + AASA and ASSIST Scores*

| Predictor | B | *β* | SE | z | p |
| --- | --- | --- | --- | --- | --- |
| CSA + AASA | -0.16 | -0.02 | 0.13 | -1.24 | .216 |
| UPPS-SE | 0.24 | 0.19 | 0.01 | 42.59 | <.001 |
| CSA + AASA x UPPS-SE | 0.08 | 0.10 | 0.01 | 6.42 | <.001 |

*Note*. CSA + AASA = Lifetime Sexual Abuse; UPPS_SE = Sensation Seeking sub-facet of the SUPPS-P scale; SE = Standard Error.

*Table AB. Simple Slopes Analysis for the Interaction Between CSA + AASA and Sensation Seeking on ASSIST Scores*

| Level of UPPS_SE | B | SE | p |
| --- | --- | --- | --- |
| Low (-1 *SD*) | 0.41 | 0.05 | <.001 |
| Mean | 0.63 | 0.04 | <.001 |
| High (+1 *SD*) | 0.85 | 0.05 | <.001 |

*Note*. UPPS_SE = Sensation Seeking sub-facet of the SUPPS-P scale; SE = Standard Error.

*Table AC. Latent Variable Interaction and Simple Slopes Analysis for Positive Urgency Moderating the Relationship Between CSA + AASA and ASSIST Scores*

| Predictor | B | *β* | SE | z | p |
| --- | --- | --- | --- | --- | --- |
| CSA + AASA | -0.32 | -0.04 | 0.14 | -2.33 | .020 |
| UPPS-PU | 0.20 | 0.15 | 0.01 | 33.32 | <.001 |
| CSA + AASA x UPPS-PU | 0.10 | 0.12 | 0.01 | 7.20 | <.001 |

*Note*. CSA + AASA = Lifetime Sexual Abuse; UPPS_PU = Positive Urgency sub-facet of the SUPPS-P scale; SE = Standard Error.

*Table AD. Simple Slopes Analysis for the Interaction Between CSA + AASA and Positive Urgency on ASSIST Scores*

| Level of UPPS_PU | B | SE | p |
| --- | --- | --- | --- |
| Low (-1 *SD*) | 0.35 | 0.05 | <.001 |
| Mean | 0.61 | 0.04 | <.001 |
| High (+1 *SD*) | 0.86 | 0.05 | <.001 |

*Note*. UPPS_PU = Positive Urgency sub-facet of the SUPPS-P scale; SE = Standard Error.
