# Supplementary material for: The moderating role of impulsivity in the association between sexual abuse and substance use frequency across countries, gender identities, and sexual orientations
Source: PLoS One. 2026 Sep 30;21(9):e0355986. doi: 10.1371/journal.pone.0355986 (PMC13626376; doi:10.1371/journal.pone.0355986)
Supplement: Supplementary file 4 [file pone.0355986.s004.docx]

### Table A. *Latent Variable Interaction Lack of Premeditation Moderating the Relationship Between CSA and ASSIST Scores Between Groups*

| Group | CSA | | | PR | | | CSA x PR | | |
| --- | --- | --- | --- | --- | --- | --- | --- | --- | --- |
|  | *B* | *β* | *p* | *B* | *β* | *p* | *B* | *β* | *p* |
| Cisgender Women | -0.12 | -0.01 | .536 | 0.27 | 0.18 | <.001 | -0.05 | -0.04 | .049 |
| Cisgender Men | -0.05 | 0.00 | .840 | 0.20 | 0.12 | <.001 | -0.01 | -0.01 | .786 |
| Gender-Diverse Individuals | -0.11 | -0.01 | .889 | 0.30 | 0.18 | <.001 | -0.11 | -0.07 | .285 |
| Heterosexual | -0.05 | -0.01 | .776 | 0.21 | 0.14 | <.001 | -0.03 | -0.02 | .184 |
| Gay or Lesbian | -1.31 | -0.11 | .061 | 0.19 | 0.12 | <.001 | 0.12 | 0.07 | .221 |
| Other Sexual Orientations | -0.11 | -0.01 | .742 | 0.28 | 0.17 | <.001 | -0.07 | -0.04 | .113 |
| Australia | 2.15 | 0.16 | .248 | 0.32 | 0.19 | <.001 | -0.37 | -0.20 | .154 |
| Austria | -0.52 | -0.05 | .752 | 0.31 | 0.17 | <.001 | 0.10 | 0.06 | .662 |
| Belgium | 1.80 | 0.17 | .258 | 0.10 | 0.07 | .128 | -0.15 | -0.11 | .481 |
| Canada | -1.22 | -0.10 | .175 | 0.17 | 0.11 | <.001 | 0.10 | 0.06 | .429 |
| Colombia | 0.01 | 0.00 | .984 | 0.14 | 0.10 | <.001 | 0.00 | 0.00 | .994 |
| Chile | -0.20 | -0.02 | .834 | 0.22 | 0.15 | <.001 | 0.00 | 0.00 | .999 |
| Croatia | -0.24 | -0.02 | .804 | 0.31 | 0.18 | <.001 | 0.02 | 0.01 | .899 |
| France | 0.60 | 0.06 | .528 | 0.21 | 0.13 | <.001 | -0.05 | -0.04 | .698 |
| Germany | 1.55 | 0.17 | .017 | 0.19 | 0.12 | <.001 | -0.22 | -0.18 | .015 |
| Hungary | 0.37 | 0.03 | .299 | 0.26 | 0.17 | <.001 | -0.06 | -0.04 | .208 |
| Ireland | 0.08 | 0.01 | .950 | 0.34 | 0.21 | <.001 | -0.15 | -0.08 | .392 |
| Israel | 0.25 | 0.02 | .818 | 0.38 | 0.23 | <.001 | -0.08 | -0.05 | .606 |
| Italy | -0.48 | -0.04 | .487 | 0.27 | 0.18 | <.001 | 0.06 | 0.04 | .534 |
| Malaysia | 0.16 | 0.02 | .849 | 0.04 | 0.03 | .330 | -0.03 | -0.03 | .783 |
| Mexico | -0.86 | -0.12 | .155 | 0.10 | 0.07 | .003 | 0.09 | 0.08 | .315 |
| New Zealand | -1.12 | -0.08 | .221 | 0.23 | 0.14 | <.001 | 0.13 | 0.07 | .304 |
| North Macedonia | -1.73 | -0.11 | .252 | 0.21 | 0.12 | <.001 | 0.25 | 0.11 | .259 |
| Peru | 0.79 | 0.11 | .116 | 0.21 | 0.16 | <.001 | -0.18 | -0.19 | .011 |
| Poland | 0.12 | 0.01 | .773 | 0.25 | 0.17 | <.001 | -0.05 | -0.04 | .352 |
| Portugal | -0.20 | -0.02 | .800 | 0.31 | 0.20 | <.001 | -0.01 | -0.01 | .898 |
| South Africa | -0.23 | -0.02 | .803 | 0.35 | 0.21 | <.001 | -0.06 | -0.04 | .644 |
| South Korea | -0.22 | -0.03 | .733 | 0.13 | 0.12 | <.001 | 0.01 | 0.01 | .950 |
| Spain | -0.92 | -0.08 | .253 | 0.25 | 0.18 | <.001 | 0.10 | 0.06 | .372 |
| Switzerland | 1.17 | 0.11 | .327 | 0.31 | 0.21 | <.001 | -0.25 | -0.16 | .136 |
| United Kingdom | -1.01 | -0.08 | .406 | 0.24 | 0.16 | <.001 | 0.07 | 0.04 | .648 |
| United States | 0.73 | 0.06 | .393 | 0.22 | 0.14 | <.001 | -0.20 | -0.12 | .090 |

*Note*. CSA = Child Sexual Abuse; PR = SUPPS-Sub Facet Lack of Premeditation; CSA x PR = Estimate of the relationship between Child Sexual Abuse and Lack of Premeditation.

### Table B. *Latent Variable Interaction Lack of Premeditation Moderating the Relationship Between AASA and ASSIST Scores Between Groups*

| Group | AASA | | | PR | | | AASA x PR | | |
| --- | --- | --- | --- | --- | --- | --- | --- | --- | --- |
|  | *B* | *β* | *p* | *B* | *β* | *p* | *B* | *β* | *p* |
| Cisgender Women | 0.59 | 0.08 | <.001 | 0.26 | 0.17 | <.001 | 0.00 | 0.00 | .930 |
| Cisgender Men | 0.63 | 0.07 | .004 | 0.19 | 0.12 | <.001 | 0.02 | 0.02 | .433 |
| Gender-Diverse Individuals | 1.15 | 0.13 | .025 | 0.31 | 0.19 | <.001 | -0.09 | -0.09 | .151 |
| Heterosexual | 0.36 | 0.05 | .005 | 0.19 | 0.13 | <.001 | 0.02 | 0.02 | .297 |
| Gay or Lesbian | 1.50 | 0.18 | .002 | 0.23 | 0.14 | <.001 | -0.10 | -0.09 | .151 |
| Other Sexual Orientations | 0.49 | 0.06 | .020 | 0.27 | 0.17 | <.001 | 0.01 | 0.01 | .718 |
| Australia | -1.02 | -0.12 | .392 | 0.21 | 0.12 | .012 | 0.24 | 0.23 | .118 |
| Austria | 1.58 | 0.21 | .149 | 0.34 | 0.19 | <.001 | -0.11 | -0.11 | .460 |
| Belgium | -0.62 | -0.09 | .569 | 0.08 | 0.05 | .284 | 0.05 | 0.05 | .740 |
| Canada | 1.01 | 0.14 | .052 | 0.20 | 0.13 | <.001 | -0.09 | -0.10 | .187 |
| Colombia | 0.99 | 0.11 | .157 | 0.15 | 0.10 | <.001 | -0.05 | -0.05 | .554 |
| Chile | 0.67 | 0.08 | .425 | 0.21 | 0.14 | <.001 | 0.02 | 0.02 | .837 |
| Croatia | 2.41 | 0.30 | <.001 | 0.38 | 0.22 | <.001 | -0.25 | -0.24 | .001 |
| France | 0.14 | 0.02 | .841 | 0.19 | 0.12 | <.001 | 0.04 | 0.04 | .649 |
| Germany | 0.19 | 0.03 | .681 | 0.15 | 0.09 | <.001 | 0.03 | 0.03 | .690 |
| Hungary | 0.19 | 0.02 | .494 | 0.24 | 0.16 | <.001 | 0.05 | 0.05 | .186 |
| Ireland | 0.59 | 0.08 | .358 | 0.33 | 0.21 | <.001 | -0.01 | -0.01 | .913 |
| Israel | 0.97 | 0.12 | .211 | 0.39 | 0.23 | <.001 | -0.07 | -0.07 | .485 |
| Italy | -0.21 | -0.03 | .675 | 0.25 | 0.17 | <.001 | 0.09 | 0.09 | .179 |
| Malaysia | -0.86 | -0.15 | .147 | -0.03 | -0.03 | .442 | 0.23 | 0.30 | .004 |
| Mexico | 0.09 | 0.01 | .870 | 0.11 | 0.08 | .003 | 0.03 | 0.03 | .712 |
| New Zealand | 1.70 | 0.21 | .002 | 0.30 | 0.18 | <.001 | -0.18 | -0.18 | .010 |
| North Macedonia | 0.97 | 0.10 | .265 | 0.22 | 0.12 | <.001 | -0.01 | -0.01 | .925 |
| Peru | -0.22 | -0.03 | .660 | 0.17 | 0.13 | <.001 | 0.05 | 0.05 | .484 |
| Poland | -0.10 | -0.02 | .714 | 0.23 | 0.15 | <.001 | 0.05 | 0.06 | .151 |
| Portugal | 1.26 | 0.17 | .019 | 0.33 | 0.21 | <.001 | -0.10 | -0.09 | .213 |
| South Africa | -0.15 | -0.02 | .808 | 0.31 | 0.19 | <.001 | 0.12 | 0.10 | .189 |
| South Korea | 0.08 | 0.01 | .899 | 0.12 | 0.11 | <.001 | 0.05 | 0.07 | .471 |
| Spain | 1.39 | 0.20 | .005 | 0.28 | 0.19 | <.001 | -0.07 | -0.08 | .271 |
| Switzerland | 1.08 | 0.15 | .155 | 0.31 | 0.20 | <.001 | -0.07 | -0.08 | .455 |
| United Kingdom | 0.63 | 0.09 | .364 | 0.24 | 0.15 | <.001 | 0.02 | 0.02 | .811 |
| United States | -0.03 | 0.00 | .955 | 0.18 | 0.12 | <.001 | 0.08 | 0.07 | .273 |

*Note*. AASA = Adult Sexual Abuse; PR = SUPPS-Sub Facet Lack of Premeditation; CSA x PR = Estimate of the relationship between Child Sexual Abuse and Lack of Premeditation.

### Table C. *Latent Variable Interaction Lack of Premeditation Moderating the Relationship Between CSA+AASA and ASSIST Scores Between Groups*

| Group | CSA+ AASA | | | PR | | | CSA +AASA x PR | | |
| --- | --- | --- | --- | --- | --- | --- | --- | --- | --- |
|  | *B* | *β* | *p* | *B* | *β* | *p* | *B* | *β* | *p* |
| Cisgender Women | 0.35 | 0.05 | .015 | 0.24 | 0.16 | <.001 | 0.05 | 0.05 | .009 |
| Cisgender Men | 0.66 | 0.05 | .022 | 0.19 | 0.12 | <.001 | 0.01 | 0.01 | .757 |
| Gender-Diverse Individuals | 0.00 | 0.00 | .998 | 0.22 | 0.13 | <.001 | 0.16 | 0.16 | .009 |
| Heterosexual | 0.25 | 0.03 | .114 | 0.20 | 0.13 | <.001 | 0.03 | 0.02 | .221 |
| Gay or Lesbian | -0.41 | -0.05 | .451 | 0.17 | 0.10 | <.001 | 0.14 | 0.12 | .058 |
| Other Sexual Orientations | 0.31 | 0.04 | .162 | 0.25 | 0.15 | <.001 | 0.07 | 0.07 | .016 |
| Australia | -0.53 | -0.06 | .682 | 0.23 | 0.14 | 0.003 | 0.23 | 0.20 | .173 |
| Austria | 1.11 | 0.12 | .430 | 0.31 | 0.17 | <.001 | -0.02 | -0.02 | .905 |
| Belgium | 0.55 | 0.06 | .690 | 0.09 | 0.06 | .198 | 0.02 | 0.01 | .926 |
| Canada | 0.31 | 0.04 | .590 | 0.15 | 0.10 | <.001 | 0.06 | 0.06 | .404 |
| Colombia | 0.08 | 0.01 | .912 | 0.12 | 0.08 | 0.002 | 0.11 | 0.10 | .243 |
| Chile | 0.56 | 0.06 | .536 | 0.20 | 0.14 | <.001 | 0.08 | 0.07 | .525 |
| Croatia | -1.65 | -0.16 | .028 | 0.24 | 0.14 | <.001 | 0.32 | 0.25 | <.001 |
| France | 0.18 | 0.02 | .811 | 0.19 | 0.12 | <.001 | 0.04 | 0.03 | .706 |
| Germany | -0.42 | -0.05 | .478 | 0.15 | 0.09 | <.001 | 0.13 | 0.11 | .126 |
| Hungary | 0.01 | 0.00 | .979 | 0.24 | 0.16 | <.001 | 0.07 | 0.06 | .077 |
| Ireland | 0.00 | 0.00 | .999 | 0.30 | 0.18 | <.001 | 0.11 | 0.10 | .231 |
| Israel | 0.30 | 0.03 | .719 | 0.34 | 0.20 | <.001 | 0.09 | 0.08 | .426 |
| Italy | 1.43 | 0.15 | .020 | 0.30 | 0.21 | <.001 | -0.15 | -0.13 | .055 |
| Malaysia | 1.79 | 0.28 | .007 | 0.06 | 0.06 | .093 | -0.17 | -0.20 | .061 |
| Mexico | 0.59 | 0.09 | .227 | 0.10 | 0.07 | .008 | 0.02 | 0.02 | .770 |
| New Zealand | 0.04 | 0.00 | .941 | 0.19 | 0.12 | <.001 | 0.12 | 0.12 | .094 |
| North Macedonia | 0.38 | 0.03 | .734 | 0.19 | 0.10 | <.001 | 0.16 | 0.10 | .307 |
| Peru | 0.28 | 0.04 | .532 | 0.15 | 0.11 | <.001 | 0.07 | 0.08 | .272 |
| Poland | -0.16 | -0.02 | .623 | 0.23 | 0.15 | <.001 | 0.09 | 0.10 | .023 |
| Portugal | 0.66 | 0.08 | .268 | 0.29 | 0.18 | <.001 | 0.04 | 0.04 | .602 |
| South Africa | 1.70 | 0.19 | .017 | 0.36 | 0.22 | <.001 | -0.12 | -0.10 | .215 |
| South Korea | 0.53 | 0.07 | .443 | 0.11 | 0.11 | <.001 | 0.04 | 0.05 | .628 |
| Spain | 0.75 | 0.08 | .277 | 0.26 | 0.18 | <.001 | -0.05 | -0.04 | .598 |
| Switzerland | 0.23 | 0.03 | .801 | 0.28 | 0.18 | <.001 | 0.08 | 0.08 | .464 |
| United Kingdom | 0.44 | 0.05 | .566 | 0.26 | 0.17 | <.001 | -0.04 | -0.04 | .703 |
| United States | -0.08 | -0.01 | .881 | 0.13 | 0.09 | <.001 | 0.18 | 0.18 | .009 |

*Note*. CSA + AASA = Lifetime Sexual Abuse; PR = SUPPS-Sub Facet Lack of Premeditation; CSA x PR = Estimate of the relationship between Child Sexual Abuse and Lack of Premeditation.

### Table D. *Latent Variable Interaction Lack of Perseverance Moderating the Relationship Between CSA and ASSIST Scores Between Groups*

| Group | CSA | | | PE | | | CSA x PE | | |
| --- | --- | --- | --- | --- | --- | --- | --- | --- | --- |
|  | *B* | *β* | *p* | *B* | *β* | *p* | *B* | *β* | *p* |
| Cisgender Women | 0.01 | 0.00 | .962 | 0.14 | 0.11 | <.001 | -0.07 | -0.05 | .003 |
| Cisgender Men | 0.13 | 0.01 | .581 | 0.11 | 0.08 | <.001 | -0.03 | -0.02 | .270 |
| Gender-Diverse Individuals | -0.29 | -0.02 | .717 | 0.09 | 0.07 | <.001 | -0.07 | -0.05 | .396 |
| Heterosexual | 0.02 | 0.00 | .894 | 0.11 | 0.08 | <.001 | -0.04 | -0.03 | .056 |
| Gay or Lesbian | -0.19 | -0.02 | .772 | 0.09 | 0.06 | <.001 | -0.04 | -0.03 | .620 |
| Other Sexual Orientations | -0.02 | 0.00 | .954 | 0.11 | 0.08 | <.001 | -0.07 | -0.05 | .038 |
| Australia | 1.86 | 0.14 | .195 | 0.22 | 0.15 | <.001 | -0.31 | -0.19 | .083 |
| Austria | 0.62 | 0.05 | .660 | 0.28 | 0.19 | <.001 | -0.06 | -0.04 | .749 |
| Belgium | -0.18 | -0.02 | .907 | 0.16 | 0.13 | .004 | 0.10 | 0.08 | .573 |
| Canada | -0.43 | -0.03 | .593 | 0.08 | 0.06 | .009 | -0.02 | -0.01 | .876 |
| Colombia | -0.33 | -0.04 | .640 | 0.15 | 0.11 | <.001 | 0.04 | 0.04 | .632 |
| Chile | 2.16 | 0.22 | .027 | 0.16 | 0.12 | <.001 | -0.29 | -0.25 | .011 |
| Croatia | 1.01 | 0.08 | .274 | 0.13 | 0.09 | <.001 | -0.15 | -0.09 | .199 |
| France | 0.07 | 0.01 | .936 | 0.01 | 0.01 | .848 | 0.02 | 0.01 | .849 |
| Germany | 0.67 | 0.07 | .260 | 0.09 | 0.07 | <.001 | -0.09 | -0.07 | .271 |
| Hungary | 0.50 | 0.05 | .143 | 0.16 | 0.12 | <.001 | -0.07 | -0.05 | .092 |
| Ireland | 0.13 | 0.01 | .900 | 0.19 | 0.14 | <.001 | -0.14 | -0.08 | .323 |
| Israel | -0.52 | -0.05 | .580 | 0.25 | 0.17 | <.001 | 0.02 | 0.01 | .885 |
| Italy | 1.01 | 0.09 | .153 | 0.16 | 0.12 | <.001 | -0.15 | -0.10 | .098 |
| Malaysia | -0.21 | -0.03 | .773 | 0.01 | 0.01 | .763 | 0.02 | 0.02 | .830 |
| Mexico | 0.41 | 0.06 | .448 | 0.10 | 0.09 | <.001 | -0.09 | -0.10 | .180 |
| New Zealand | -0.72 | -0.05 | .405 | 0.12 | 0.08 | <.001 | 0.05 | 0.04 | .589 |
| North Macedonia | 0.27 | 0.02 | .849 | 0.11 | 0.07 | .022 | -0.06 | -0.03 | .773 |
| Peru | 0.36 | 0.05 | .466 | 0.14 | 0.11 | <.001 | -0.11 | -0.13 | .077 |
| Poland | 0.64 | 0.06 | .105 | 0.12 | 0.09 | <.001 | -0.11 | -0.09 | .018 |
| Portugal | -1.66 | -0.17 | .028 | 0.09 | 0.07 | .004 | 0.19 | 0.14 | .058 |
| South Africa | -0.79 | -0.07 | .346 | 0.18 | 0.13 | <.001 | 0.03 | 0.02 | .795 |
| South Korea | -0.27 | -0.04 | .640 | 0.10 | 0.11 | <.001 | 0.01 | 0.01 | .919 |
| Spain | -1.17 | -0.10 | .143 | 0.21 | 0.15 | <.001 | 0.11 | 0.08 | .251 |
| Switzerland | -1.21 | -0.11 | .279 | 0.11 | 0.09 | .008 | 0.07 | 0.05 | .615 |
| United Kingdom | -1.59 | -0.12 | .188 | 0.10 | 0.08 | .010 | 0.13 | 0.09 | .337 |
| United States | 0.33 | 0.03 | .682 | 0.13 | 0.10 | <.001 | -0.13 | -0.09 | .173 |

*Note*. CSA = Child Sexual Abuse; PE = SUPPS-Sub Facet Lack of Perseverance; CSA x PE = Estimate of the relationship between Child Sexual Abuse and Lack of Perseverance.

### Table E. *Latent Variable Interaction Lack of Perseverance Moderating the Relationship Between AASA and ASSIST Scores Between Groups*

| Group | AASA | | | PE | | | AASA x PE | | |
| --- | --- | --- | --- | --- | --- | --- | --- | --- | --- |
|  | *B* | *β* | *p* | *B* | *β* | *p* | *B* | *β* | *p* |
| Cisgender Women | 0.53 | 0.08 | <.001 | 0.13 | 0.10 | <.001 | 0.01 | 0.01 | 0.451 |
| Cisgender Men | 0.45 | 0.05 | 0.031 | 0.10 | 0.07 | <.001 | 0.05 | 0.04 | 0.073 |
| Gender-Diverse Individuals | 0.71 | 0.08 | 0.187 | 0.09 | 0.06 | 0.003 | -0.03 | -0.03 | 0.639 |
| Heterosexual | 0.24 | 0.03 | .049 | 0.10 | 0.07 | <.001 | 0.04 | 0.04 | .023 |
| Gay or Lesbian | 0.66 | 0.08 | .170 | 0.08 | 0.06 | .007 | 0.02 | 0.02 | .702 |
| Other Sexual Orientations | 0.66 | 0.09 | .001 | 0.10 | 0.07 | <.001 | -0.01 | -0.01 | .736 |
| Australia | 1.01 | 0.12 | .409 | 0.17 | 0.12 | .013 | -0.03 | -0.03 | .860 |
| Austria | -1.25 | -0.16 | .184 | 0.19 | 0.13 | .004 | 0.27 | 0.29 | .022 |
| Belgium | 0.47 | 0.06 | .636 | 0.20 | 0.16 | .001 | -0.09 | -0.10 | .447 |
| Canada | 0.67 | 0.09 | .181 | 0.08 | 0.06 | .014 | -0.04 | -0.04 | .548 |
| Colombia | 0.24 | 0.03 | .730 | 0.15 | 0.11 | <.001 | 0.05 | 0.05 | .572 |
| Chile | 0.87 | 0.11 | .321 | 0.11 | 0.08 | .013 | 0.00 | 0.00 | .980 |
| Croatia | 2.35 | 0.29 | <.001 | 0.19 | 0.13 | <.001 | -0.23 | -0.23 | <.001 |
| France | 0.16 | 0.02 | .798 | 0.00 | 0.00 | .983 | 0.04 | 0.04 | .612 |
| Germany | -0.22 | -0.03 | .600 | 0.06 | 0.04 | .041 | 0.08 | 0.10 | .121 |
| Hungary | 0.39 | 0.05 | .133 | 0.15 | 0.11 | <.001 | 0.02 | 0.02 | .463 |
| Ireland | 0.15 | 0.02 | .814 | 0.17 | 0.12 | <.001 | 0.04 | 0.05 | .561 |
| Israel | 0.20 | 0.02 | .787 | 0.24 | 0.16 | <.001 | 0.05 | 0.04 | .627 |
| Italy | -0.82 | -0.11 | .096 | 0.09 | 0.07 | .008 | 0.16 | 0.18 | .009 |
| Malaysia | 1.65 | 0.29 | .006 | 0.03 | 0.03 | .310 | -0.11 | -0.16 | .132 |
| Mexico | 0.17 | 0.03 | .735 | 0.08 | 0.07 | .009 | 0.01 | 0.02 | .845 |
| New Zealand | 0.88 | 0.11 | .085 | 0.14 | 0.10 | <.001 | -0.07 | -0.07 | .275 |
| North Macedonia | 0.74 | 0.08 | .348 | 0.10 | 0.06 | .071 | 0.03 | 0.02 | .795 |
| Peru | 0.26 | 0.04 | .600 | 0.12 | 0.10 | <.001 | -0.02 | -0.02 | .790 |
| Poland | -0.44 | -0.07 | .095 | 0.08 | 0.06 | <.001 | 0.09 | 0.12 | .003 |
| Portugal | 1.02 | 0.14 | .056 | 0.12 | 0.09 | <.001 | -0.05 | -0.06 | .447 |
| South Africa | 1.24 | 0.15 | .042 | 0.20 | 0.14 | <.001 | -0.08 | -0.08 | .302 |
| South Korea | 0.28 | 0.04 | .630 | 0.09 | 0.10 | .001 | 0.03 | 0.04 | .678 |
| Spain | 1.38 | 0.20 | .005 | 0.24 | 0.18 | <.001 | -0.06 | -0.07 | .354 |
| Switzerland | -0.64 | -0.09 | .351 | 0.06 | 0.05 | .215 | 0.17 | 0.19 | .049 |
| United Kingdom | 1.32 | 0.18 | .055 | 0.13 | 0.10 | .003 | -0.06 | -0.07 | .475 |
| United States | -0.18 | -0.02 | .733 | 0.09 | 0.07 | .006 | 0.09 | 0.10 | .150 |

*Note*. AASA = Adult Sexual Abuse; PE = SUPPS-Sub Facet Lack of Perseverance; CSA x PE = Estimate of the relationship between Child Sexual Abuse and Lack of Perseverance.

### Table F. *Latent Variable Interaction Lack of Perseverance Moderating the Relationship Between CSA+AASA and ASSIST Scores Between Groups*

| Group | CSA+ AASA | | | PE | | | CSA +AASA x PE | | |
| --- | --- | --- | --- | --- | --- | --- | --- | --- | --- |
|  | *B* | *β* | *p* | *B* | *β* | *p* | *B* | *β* | *p* |
| Cisgender Women | 0.33 | 0.04 | .014 | 0.12 | 0.09 | <.001 | 0.05 | 0.06 | .002 |
| Cisgender Men | 0.38 | 0.03 | .177 | 0.10 | 0.07 | <.001 | 0.05 | 0.03 | .155 |
| Gender-Diverse Individuals | 0.16 | 0.02 | .753 | 0.03 | 0.02 | .303 | 0.12 | 0.14 | .021 |
| Heterosexual | 0.25 | 0.03 | .092 | 0.10 | 0.08 | <.001 | 0.03 | 0.02 | .142 |
| Gay or Lesbian | -0.43 | -0.05 | .419 | 0.05 | 0.04 | .079 | 0.13 | 0.12 | .038 |
| Other Sexual Orientations | 0.19 | 0.02 | .370 | 0.07 | 0.05 | <.001 | 0.08 | 0.09 | .001 |
| Australia | -1.20 | -0.13 | .311 | 0.08 | 0.06 | .232 | 0.29 | 0.29 | .036 |
| Austria | 1.00 | 0.11 | .403 | 0.26 | 0.18 | <.001 | -0.01 | -0.01 | .918 |
| Belgium | -1.68 | -0.19 | .205 | 0.15 | 0.11 | .010 | 0.31 | 0.28 | .059 |
| Canada | 0.57 | 0.07 | .310 | 0.06 | 0.04 | .076 | 0.03 | 0.03 | .674 |
| Colombia | 1.04 | 0.12 | .147 | 0.15 | 0.11 | <.001 | -0.02 | -0.02 | .795 |
| Chile | -0.44 | -0.05 | .629 | 0.08 | 0.06 | .063 | 0.20 | 0.19 | .068 |
| Croatia | -0.38 | -0.04 | .577 | 0.08 | 0.05 | .028 | 0.16 | 0.13 | .061 |
| France | 0.91 | 0.11 | .205 | 0.02 | 0.01 | .663 | -0.04 | -0.04 | .617 |
| Germany | -0.15 | -0.02 | .768 | 0.07 | 0.05 | .010 | 0.08 | 0.08 | .219 |
| Hungary | 0.05 | 0.00 | .880 | 0.14 | 0.10 | <.001 | 0.07 | 0.06 | .057 |
| Ireland | 0.33 | 0.04 | .644 | 0.17 | 0.12 | <.001 | 0.06 | 0.06 | .449 |
| Israel | 2.27 | 0.26 | .004 | 0.28 | 0.19 | <.001 | -0.18 | -0.16 | .082 |
| Italy | 0.87 | 0.09 | .161 | 0.15 | 0.12 | <.001 | -0.06 | -0.05 | .420 |
| Malaysia | -0.39 | -0.06 | .540 | -0.01 | -0.01 | .844 | 0.12 | 0.16 | .108 |
| Mexico | 0.16 | 0.03 | .723 | 0.05 | 0.04 | .157 | 0.08 | 0.10 | .189 |
| New Zealand | -0.12 | -0.01 | .823 | 0.07 | 0.05 | .023 | 0.14 | 0.14 | .036 |
| North Macedonia | 0.56 | 0.05 | .579 | 0.08 | 0.05 | .121 | 0.13 | 0.08 | .319 |
| Peru | 0.29 | 0.05 | .518 | 0.09 | 0.08 | .002 | 0.07 | 0.08 | .247 |
| Poland | 0.46 | 0.06 | .130 | 0.10 | 0.08 | <.001 | 0.02 | 0.02 | .637 |
| Portugal | 0.95 | 0.12 | .102 | 0.10 | 0.08 | .002 | 0.01 | 0.01 | .843 |
| South Africa | 0.08 | 0.01 | .911 | 0.14 | 0.10 | <.001 | 0.11 | 0.10 | .185 |
| South Korea | 0.00 | 0.00 | .994 | 0.07 | 0.08 | .005 | 0.10 | 0.13 | .136 |
| Spain | 0.33 | 0.04 | .625 | 0.21 | 0.16 | <.001 | 0.01 | 0.01 | .893 |
| Switzerland | 1.57 | 0.18 | .056 | 0.13 | 0.10 | .003 | -0.09 | -0.08 | .386 |
| United Kingdom | -0.85 | -0.10 | .247 | 0.07 | 0.05 | .111 | 0.12 | 0.14 | .141 |
| United States | 0.36 | 0.04 | .514 | 0.08 | 0.06 | .022 | 0.11 | 0.12 | .080 |

*Note*. CSA + AASA = Lifetime Sexual Abuse; PE = SUPPS-Sub Facet Lack of Perseverance; CSA x PE = Estimate of the relationship between Child Sexual Abuse and Lack of Perseverance.

### Table G. *Latent Variable Interaction Negative Urgency Moderating the Relationship Between CSA and ASSIST Scores Between Groups*

| Group | CSA | | | NU | | | CSA x NU | | |
| --- | --- | --- | --- | --- | --- | --- | --- | --- | --- |
|  | *B* | *β* | *p* | *B* | *β* | *p* | *B* | *β* | *p* |
| Cisgender Women | -0.06 | -0.01 | .772 | 0.15 | 0.12 | <.001 | -0.05 | -0.05 | .015 |
| Cisgender Men | 0.05 | 0.00 | .835 | 0.10 | 0.08 | <.001 | -0.02 | -0.02 | .469 |
| Gender-Diverse Individuals | 0.18 | 0.01 | .820 | 0.14 | 0.11 | <.001 | -0.12 | -0.09 | .129 |
| Heterosexual | -0.01 | 0.00 | .948 | 0.10 | 0.08 | <.001 | -0.03 | -0.03 | .097 |
| Gay or Lesbian | -0.81 | -0.07 | .233 | 0.09 | 0.07 | <.001 | 0.03 | 0.03 | .654 |
| Other Sexual Orientations | 0.22 | 0.02 | .495 | 0.16 | 0.13 | <.001 | -0.09 | -0.07 | .006 |
| Australia | -0.41 | -0.03 | .790 | 0.06 | 0.04 | .324 | -0.02 | -0.01 | .928 |
| Austria | 0.95 | 0.08 | .555 | 0.13 | 0.10 | .013 | -0.08 | -0.07 | .623 |
| Belgium | -0.21 | -0.02 | .895 | 0.06 | 0.05 | .280 | 0.12 | 0.09 | .521 |
| Canada | 0.91 | 0.07 | .251 | 0.13 | 0.11 | <.001 | -0.17 | -0.12 | .056 |
| Colombia | -0.27 | -0.03 | .718 | -0.01 | -0.01 | .817 | 0.03 | 0.04 | .667 |
| Chile | -0.28 | -0.03 | .776 | 0.04 | 0.03 | .289 | 0.01 | 0.01 | .905 |
| Croatia | -0.86 | -0.06 | .385 | 0.17 | 0.13 | <.001 | 0.08 | 0.06 | .436 |
| France | -0.13 | -0.01 | .885 | 0.09 | 0.08 | .005 | 0.04 | 0.03 | .704 |
| Germany | 1.16 | 0.13 | .064 | 0.11 | 0.09 | <.001 | -0.13 | -0.13 | .057 |
| Hungary | -0.10 | -0.01 | .764 | 0.13 | 0.11 | <.001 | 0.00 | 0.00 | .901 |
| Ireland | 0.73 | 0.06 | .535 | 0.22 | 0.17 | <.001 | -0.18 | -0.13 | .137 |
| Israel | -0.09 | -0.01 | .932 | 0.17 | 0.13 | <.001 | -0.03 | -0.03 | .771 |
| Italy | 0.57 | 0.05 | .503 | 0.16 | 0.13 | <.001 | -0.07 | -0.06 | .431 |
| Malaysia | -1.33 | -0.18 | .088 | 0.01 | 0.01 | .790 | 0.13 | 0.18 | .090 |
| Mexico | 0.39 | 0.05 | .482 | 0.08 | 0.08 | .003 | -0.07 | -0.10 | .204 |
| New Zealand | -0.13 | -0.01 | .878 | 0.16 | 0.13 | <.001 | -0.01 | -0.01 | .895 |
| North Macedonia | -0.59 | -0.04 | .673 | 0.14 | 0.10 | <.001 | 0.05 | 0.03 | .733 |
| Peru | -0.17 | -0.02 | .713 | 0.09 | 0.09 | <.001 | -0.03 | -0.05 | .498 |
| Poland | 0.09 | 0.01 | .830 | 0.15 | 0.13 | <.001 | -0.04 | -0.04 | .383 |
| Portugal | 0.13 | 0.01 | .862 | 0.14 | 0.12 | <.001 | -0.05 | -0.05 | .535 |
| South Africa | -1.36 | -0.12 | .144 | 0.22 | 0.18 | <.001 | 0.08 | 0.06 | .430 |
| South Korea | 0.31 | 0.05 | .615 | 0.08 | 0.10 | .001 | -0.05 | -0.08 | .399 |
| Spain | 0.87 | 0.08 | .306 | 0.14 | 0.12 | <.001 | -0.13 | -0.10 | .156 |
| Switzerland | -0.45 | -0.04 | .683 | 0.19 | 0.15 | <.001 | -0.03 | -0.02 | .817 |
| United Kingdom | 0.19 | 0.01 | .870 | 0.18 | 0.16 | <.001 | -0.07 | -0.05 | .588 |
| United States | 1.11 | 0.09 | .151 | 0.16 | 0.13 | <.001 | -0.20 | -0.15 | .016 |

*Note*. CSA = Child Sexual Abuse; NU = SUPPS-Sub Facet Negative Urgency; CSA x NU = Estimate of the relationship between Child Sexual Abuse and Negative Urgency.

### Table H. *Latent Variable Interaction Negative Urgency Moderating the Relationship Between AASA and ASSIST Scores Between Groups*

| Group | AASA | | | NU | | | AASA x NU | | |
| --- | --- | --- | --- | --- | --- | --- | --- | --- | --- |
|  | *B* | *β* | *p* | *B* | *β* | *p* | *B* | *β* | *p* |
| Cisgender Women | 0.43 | 0.06 | <.001 | 0.13 | 0.11 | <.001 | 0.02 | 0.03 | .166 |
| Cisgender Men | 0.49 | 0.05 | .018 | 0.08 | 0.07 | <.001 | 0.04 | 0.04 | .109 |
| Gender-Diverse Individuals | 0.68 | 0.08 | .190 | 0.13 | 0.10 | <.001 | -0.02 | -0.02 | .703 |
| Heterosexual | 0.15 | 0.02 | .244 | 0.08 | 0.07 | <.001 | 0.04 | 0.05 | .005 |
| Gay or Lesbian | 1.68 | 0.20 | <.001 | 0.12 | 0.09 | <.001 | -0.09 | -0.11 | .072 |
| Other Sexual Orientations | 0.33 | 0.04 | .117 | 0.14 | 0.11 | <.001 | 0.03 | 0.03 | .214 |
| Australia | 0.74 | 0.09 | .527 | 0.05 | 0.04 | .417 | 0.02 | 0.02 | .901 |
| Austria | 1.89 | 0.25 | .070 | 0.14 | 0.11 | .017 | -0.12 | -0.16 | .271 |
| Belgium | -0.65 | -0.09 | .514 | 0.06 | 0.05 | .341 | 0.04 | 0.05 | .702 |
| Canada | 0.35 | 0.05 | .467 | 0.11 | 0.09 | <.001 | 0.00 | 0.01 | .940 |
| Colombia | 0.75 | 0.09 | .296 | 0.00 | 0.00 | .993 | -0.02 | -0.02 | .795 |
| Chile | 0.14 | 0.02 | .864 | 0.02 | 0.02 | .585 | 0.08 | 0.10 | .343 |
| Croatia | 0.99 | 0.12 | .084 | 0.19 | 0.14 | <.001 | -0.05 | -0.06 | .388 |
| France | 0.27 | 0.03 | .689 | 0.09 | 0.07 | .012 | 0.02 | 0.02 | .787 |
| Germany | -0.35 | -0.05 | .441 | 0.07 | 0.06 | .006 | 0.08 | 0.12 | .088 |
| Hungary | 0.87 | 0.11 | <.001 | 0.13 | 0.11 | <.001 | -0.04 | -0.05 | .184 |
| Ireland | -0.13 | -0.02 | .850 | 0.18 | 0.14 | <.001 | 0.07 | 0.09 | .305 |
| Israel | 0.58 | 0.07 | .453 | 0.16 | 0.13 | <.001 | -0.02 | -0.02 | .841 |
| Italy | -0.03 | 0.00 | .961 | 0.14 | 0.12 | <.001 | 0.05 | 0.07 | .373 |
| Malaysia | 1.11 | 0.19 | .069 | 0.02 | 0.03 | .398 | -0.04 | -0.06 | .554 |
| Mexico | 0.15 | 0.02 | .770 | 0.06 | 0.06 | .030 | 0.01 | 0.02 | .818 |
| New Zealand | 1.12 | 0.14 | .040 | 0.18 | 0.14 | <.001 | -0.08 | -0.10 | .146 |
| North Macedonia | 0.03 | 0.00 | .970 | 0.11 | 0.08 | .014 | 0.09 | 0.09 | .349 |
| Peru | -0.12 | -0.02 | .798 | 0.07 | 0.08 | <.001 | 0.03 | 0.04 | .566 |
| Poland | -0.12 | -0.02 | .671 | 0.14 | 0.11 | <.001 | 0.04 | 0.07 | .125 |
| Portugal | 0.41 | 0.06 | .456 | 0.12 | 0.11 | <.001 | 0.02 | 0.03 | .674 |
| South Africa | 1.09 | 0.14 | .085 | 0.23 | 0.19 | <.001 | -0.05 | -0.06 | .426 |
| South Korea | 0.00 | 0.00 | .995 | 0.06 | 0.07 | .017 | 0.05 | 0.09 | .357 |
| Spain | 0.44 | 0.06 | .396 | 0.11 | 0.09 | <.001 | 0.05 | 0.07 | .334 |
| Switzerland | 1.64 | 0.23 | .025 | 0.21 | 0.17 | <.001 | -0.12 | -0.17 | .120 |
| United Kingdom | 0.73 | 0.10 | .283 | 0.17 | 0.15 | <.001 | 0.00 | 0.00 | .966 |
| United States | -0.14 | -0.02 | .788 | 0.11 | 0.10 | <.001 | 0.07 | 0.09 | .190 |

*Note*. AASA = Adult Sexual Abuse; NU = SUPPS-Sub Facet Negative Urgency; CSA x NU = Estimate of the relationship between Child Sexual Abuse and Negative Urgency.

### Table I. *Latent Variable Interaction Negative Urgency Moderating the Relationship Between CSA+AASA and ASSIST Scores Between Groups*

| Group | CSA+ AASA | | | NU | | | CSA +AASA x NU | | |
| --- | --- | --- | --- | --- | --- | --- | --- | --- | --- |
|  | *B* | *β* | *p* | *B* | *β* | *p* | *B* | *β* | *p* |
| Cisgender Women | 0.46 | 0.06 | .002 | 0.13 | 0.11 | <.001 | 0.03 | 0.04 | .053 |
| Cisgender Men | 0.25 | 0.02 | .371 | 0.08 | 0.07 | <.001 | 0.05 | 0.04 | .069 |
| Gender-Diverse Individuals | -0.53 | -0.06 | .283 | 0.05 | 0.04 | .077 | 0.19 | 0.23 | <.001 |
| Heterosexual | 0.35 | 0.04 | .025 | 0.09 | 0.08 | <.001 | 0.01 | 0.01 | .598 |
| Gay or Lesbian | -1.46 | -0.16 | .008 | 0.03 | 0.03 | .207 | 0.22 | 0.24 | <.001 |
| Other Sexual Orientations | 0.01 | 0.00 | .982 | 0.12 | 0.10 | <.001 | 0.09 | 0.11 | <.001 |
| Australia | 1.13 | 0.13 | .320 | 0.05 | 0.04 | .395 | 0.01 | 0.01 | .912 |
| Austria | -1.60 | -0.17 | .180 | 0.07 | 0.05 | .222 | 0.29 | 0.30 | .020 |
| Belgium | -0.91 | -0.10 | .468 | 0.04 | 0.03 | .461 | 0.20 | 0.19 | .186 |
| Canada | 0.03 | 0.00 | .960 | 0.08 | 0.07 | .004 | 0.08 | 0.10 | .141 |
| Colombia | -0.52 | -0.06 | .478 | -0.03 | -0.02 | .337 | 0.15 | 0.18 | .041 |
| Chile | 0.17 | 0.02 | .846 | 0.02 | 0.02 | .563 | 0.11 | 0.12 | .232 |
| Croatia | -0.39 | -0.04 | .588 | 0.15 | 0.12 | <.001 | 0.13 | 0.13 | .084 |
| France | 1.14 | 0.14 | .097 | 0.11 | 0.09 | .003 | -0.07 | -0.08 | .332 |
| Germany | 1.25 | 0.15 | .029 | 0.10 | 0.09 | <.001 | -0.09 | -0.11 | .144 |
| Hungary | -0.04 | 0.00 | .888 | 0.12 | 0.10 | <.001 | 0.07 | 0.07 | .035 |
| Ireland | 0.47 | 0.06 | .546 | 0.19 | 0.15 | <.001 | 0.03 | 0.04 | .679 |
| Israel | 1.16 | 0.13 | .179 | 0.16 | 0.13 | <.001 | -0.02 | -0.02 | .858 |
| Italy | 0.92 | 0.10 | .236 | 0.16 | 0.13 | <.001 | -0.06 | -0.07 | .413 |
| Malaysia | 1.71 | 0.27 | .014 | 0.04 | 0.05 | .171 | -0.12 | -0.19 | .091 |
| Mexico | 0.27 | 0.04 | .570 | 0.04 | 0.04 | .113 | 0.05 | 0.08 | .274 |
| New Zealand | -0.53 | -0.06 | .365 | 0.10 | 0.08 | <.001 | 0.15 | 0.18 | .009 |
| North Macedonia | 1.20 | 0.10 | .276 | 0.14 | 0.10 | .002 | 0.03 | 0.03 | .781 |
| Peru | 0.56 | 0.09 | .217 | 0.06 | 0.07 | .004 | 0.02 | 0.04 | .597 |
| Poland | -0.21 | -0.03 | .540 | 0.13 | 0.11 | <.001 | 0.08 | 0.10 | .019 |
| Portugal | 0.54 | 0.07 | .387 | 0.11 | 0.10 | <.001 | 0.05 | 0.06 | .422 |
| South Africa | 1.08 | 0.12 | .139 | 0.22 | 0.18 | <.001 | -0.02 | -0.02 | .770 |
| South Korea | -0.36 | -0.05 | .584 | 0.04 | 0.05 | .100 | 0.12 | 0.18 | .055 |
| Spain | 0.22 | 0.02 | .754 | 0.12 | 0.10 | <.001 | 0.02 | 0.02 | .787 |
| Switzerland | -1.26 | -0.14 | .174 | 0.14 | 0.11 | <.001 | 0.24 | 0.25 | .017 |
| United Kingdom | 0.34 | 0.04 | .652 | 0.18 | 0.16 | <.001 | -0.02 | -0.02 | .826 |
| United States | 0.07 | 0.01 | .899 | 0.09 | 0.08 | .002 | 0.12 | 0.15 | .026 |

*Note*. CSA + AASA = Lifetime Sexual Abuse; NU = SUPPS-Sub Facet Negative Urgency; CSA x NU = Estimate of the relationship between Child Sexual Abuse and Negative Urgency.

### Table J. *Latent Variable Interaction Positive Urgency Moderating the Relationship Between CSA and ASSIST Scores Between Groups*

| Group | CSA | | | PU | | | CSA x PU | | |
| --- | --- | --- | --- | --- | --- | --- | --- | --- | --- |
|  | *B* | *β* | *p* | *B* | *β* | *p* | *B* | *β* | *p* |
| Cisgender Women | 0.02 | 0.00 | .931 | 0.25 | 0.19 | <.001 | -0.05 | -0.05 | .017 |
| Cisgender Men | -0.14 | -0.01 | .628 | 0.19 | 0.14 | <.001 | 0.00 | 0.00 | .966 |
| Gender-Diverse Individuals | 1.25 | 0.10 | .162 | 0.30 | 0.20 | <.001 | -0.21 | -0.18 | .013 |
| Heterosexual | -0.19 | -0.02 | .311 | 0.20 | 0.15 | <.001 | -0.01 | -0.01 | .682 |
| Gay or Lesbian | 0.06 | 0.00 | .943 | 0.22 | 0.15 | <.001 | -0.06 | -0.05 | .498 |
| Other Sexual Orientations | 0.22 | 0.02 | .539 | 0.27 | 0.19 | <.001 | -0.08 | -0.07 | .020 |
| Australia | 1.41 | 0.11 | .457 | 0.17 | 0.12 | 0.008 | -0.21 | -0.15 | .289 |
| Austria | 1.00 | 0.09 | .604 | 0.31 | 0.21 | <.001 | -0.08 | -0.06 | .699 |
| Belgium | 0.01 | 0.00 | .997 | 0.08 | 0.05 | .224 | 0.08 | 0.07 | .720 |
| Canada | 0.08 | 0.01 | .943 | 0.17 | 0.12 | <.001 | -0.06 | -0.05 | .584 |
| Colombia | -0.89 | -0.11 | .263 | 0.17 | 0.14 | <.001 | 0.10 | 0.12 | .224 |
| Chile | 0.87 | 0.09 | .437 | 0.25 | 0.19 | <.001 | -0.11 | -0.11 | .356 |
| Croatia | -1.31 | -0.10 | .172 | 0.34 | 0.23 | <.001 | 0.15 | 0.10 | .165 |
| France | -1.17 | -0.11 | .260 | 0.20 | 0.15 | <.001 | 0.14 | 0.14 | .163 |
| Germany | 0.69 | 0.08 | .329 | 0.17 | 0.13 | <.001 | -0.08 | -0.08 | .310 |
| Hungary | -0.85 | -0.08 | .039 | 0.24 | 0.19 | <.001 | 0.08 | 0.08 | .049 |
| Ireland | 3.76 | 0.28 | .004 | 0.38 | 0.26 | <.001 | -0.50 | -0.36 | <.001 |
| Israel | 1.53 | 0.14 | .177 | 0.35 | 0.24 | <.001 | -0.21 | -0.18 | .087 |
| Italy | 0.70 | 0.06 | .321 | 0.29 | 0.24 | <.001 | -0.09 | -0.07 | .273 |
| Malaysia | -0.79 | -0.11 | .428 | 0.07 | 0.07 | .035 | 0.07 | 0.10 | .451 |
| Mexico | 0.52 | 0.07 | .382 | 0.18 | 0.17 | <.001 | -0.09 | -0.11 | .166 |
| New Zealand | 1.26 | 0.09 | .223 | 0.31 | 0.21 | <.001 | -0.16 | -0.12 | .124 |
| North Macedonia | -2.02 | -0.13 | .176 | 0.30 | 0.20 | <.001 | 0.22 | 0.14 | .161 |
| Peru | 0.19 | 0.03 | .724 | 0.18 | 0.17 | <.001 | -0.07 | -0.10 | .214 |
| Poland | 0.07 | 0.01 | .886 | 0.25 | 0.18 | <.001 | -0.03 | -0.03 | .492 |
| Portugal | 0.71 | 0.07 | .363 | 0.29 | 0.23 | <.001 | -0.10 | -0.09 | .220 |
| South Africa | -0.39 | -0.03 | .713 | 0.33 | 0.25 | <.001 | -0.02 | -0.02 | .819 |
| South Korea | 0.82 | 0.12 | .136 | 0.15 | 0.19 | <.001 | -0.11 | -0.16 | .054 |
| Spain | 0.30 | 0.03 | .752 | 0.28 | 0.21 | <.001 | -0.06 | -0.05 | .541 |
| Switzerland | -2.32 | -0.21 | .098 | 0.20 | 0.15 | <.001 | 0.18 | 0.16 | .204 |
| United Kingdom | -0.79 | -0.06 | .559 | 0.22 | 0.16 | <.001 | 0.04 | 0.03 | .779 |
| United States | 0.46 | 0.04 | .623 | 0.21 | 0.16 | <.001 | -0.12 | -0.09 | .217 |

*Note*. CSA = Child Sexual Abuse; PU = SUPPS-Sub Facet Positive Urgency; CSA x PU = Estimate of the relationship between Child Sexual Abuse and Positive Urgency.

### Table K. *Latent Variable Interaction Positive Urgency Moderating the Relationship Between AASA and ASSIST Scores Between Groups*

| Group | AASA | | | PU | | | AASA x PU | | |
| --- | --- | --- | --- | --- | --- | --- | --- | --- | --- |
|  | *B* | *β* | *p* | *B* | *β* | *p* | *B* | *β* | *p* |
| Cisgender Women | 0.48 | 0.07 | <.001 | 0.24 | 0.18 | <.001 | 0.01 | 0.01 | .501 |
| Cisgender Men | 0.56 | 0.06 | .022 | 0.18 | 0.14 | <.001 | 0.02 | 0.02 | .417 |
| Gender-Diverse Individuals | 1.14 | 0.13 | .061 | 0.29 | 0.20 | <.001 | -0.07 | -0.08 | .262 |
| Heterosexual | 0.25 | 0.03 | .080 | 0.18 | 0.15 | <.001 | 0.02 | 0.03 | .121 |
| Gay or Lesbian | 0.93 | 0.11 | .105 | 0.22 | 0.15 | <.001 | -0.01 | -0.02 | .803 |
| Other Sexual Orientations | 0.55 | 0.07 | .021 | 0.26 | 0.18 | <.001 | 0.00 | 0.00 | .989 |
| Australia | 1.55 | 0.19 | .269 | 0.17 | 0.11 | 0.023 | -0.07 | -0.09 | .604 |
| Austria | 1.19 | 0.16 | .299 | 0.31 | 0.20 | <.001 | -0.05 | -0.07 | .650 |
| Belgium | -1.37 | -0.19 | .307 | 0.05 | 0.04 | .445 | 0.11 | 0.15 | .411 |
| Canada | -0.11 | -0.01 | .868 | 0.15 | 0.11 | <.001 | 0.05 | 0.06 | .460 |
| Colombia | 0.07 | 0.01 | .928 | 0.18 | 0.14 | <.001 | 0.05 | 0.06 | .539 |
| Chile | 0.83 | 0.10 | .364 | 0.23 | 0.18 | <.001 | 0.00 | 0.00 | .983 |
| Croatia | 1.40 | 0.17 | .022 | 0.38 | 0.26 | <.001 | -0.10 | -0.11 | .137 |
| France | 0.23 | 0.03 | .775 | 0.21 | 0.16 | <.001 | 0.02 | 0.03 | .776 |
| Germany | -0.15 | -0.02 | .765 | 0.15 | 0.11 | <.001 | 0.06 | 0.08 | .291 |
| Hungary | 0.73 | 0.09 | .018 | 0.24 | 0.19 | <.001 | -0.03 | -0.03 | .382 |
| Ireland | -0.51 | -0.07 | .505 | 0.30 | 0.21 | <.001 | 0.10 | 0.14 | .179 |
| Israel | 0.29 | 0.04 | .741 | 0.31 | 0.22 | <.001 | 0.01 | 0.02 | .881 |
| Italy | 0.09 | 0.01 | .848 | 0.27 | 0.22 | <.001 | 0.03 | 0.04 | .603 |
| Malaysia | 1.58 | 0.28 | .030 | 0.09 | 0.09 | .011 | -0.08 | -0.15 | .241 |
| Mexico | 0.36 | 0.05 | .516 | 0.17 | 0.16 | <.001 | -0.01 | -0.02 | .814 |
| New Zealand | -0.84 | -0.11 | .206 | 0.26 | 0.17 | <.001 | 0.12 | 0.15 | .071 |
| North Macedonia | -1.17 | -0.12 | .253 | 0.26 | 0.17 | <.001 | 0.19 | 0.21 | .058 |
| Peru | -1.07 | -0.15 | .057 | 0.14 | 0.13 | <.001 | 0.12 | 0.18 | .030 |
| Poland | 0.56 | 0.08 | .070 | 0.26 | 0.19 | <.001 | -0.03 | -0.04 | .363 |
| Portugal | 1.32 | 0.18 | .025 | 0.29 | 0.24 | <.001 | -0.08 | -0.11 | .190 |
| South Africa | 1.01 | 0.13 | .163 | 0.34 | 0.25 | <.001 | -0.05 | -0.06 | .529 |
| South Korea | -0.04 | -0.01 | .943 | 0.12 | 0.15 | <.001 | 0.06 | 0.09 | .305 |
| Spain | 0.95 | 0.13 | .117 | 0.27 | 0.21 | <.001 | -0.01 | -0.01 | .878 |
| Switzerland | 1.61 | 0.22 | .073 | 0.24 | 0.18 | <.001 | -0.11 | -0.16 | .223 |
| United Kingdom | 1.71 | 0.23 | .030 | 0.24 | 0.18 | <.001 | -0.10 | -0.14 | .212 |
| United States | 0.14 | 0.02 | .820 | 0.19 | 0.14 | <.001 | 0.04 | 0.05 | .541 |

*Note*. AASA = Adult Sexual Abuse; PU = SUPPS-Sub Facet Positive Urgency; CSA x PU = Estimate of the relationship between Child Sexual Abuse and Positive Urgency.

### Table L. *Latent Variable Interaction Positive Urgency Moderating the Relationship Between CSA+AASA and ASSIST Scores Between Groups*

| Group | CSA+ AASA | | | PU | | | CSA +AASA x PU | | |
| --- | --- | --- | --- | --- | --- | --- | --- | --- | --- |
|  | *B* | *β* | *p* | *B* | *β* | *p* | *B* | *β* | *p* |
| Cisgender Women | -0.02 | 0.00 | .915 | 0.22 | 0.17 | <.001 | 0.07 | 0.09 | <.001 |
| Cisgender Men | 0.16 | 0.01 | .634 | 0.18 | 0.14 | <.001 | 0.05 | 0.04 | .127 |
| Gender-Diverse Individuals | -1.33 | -0.16 | .023 | 0.17 | 0.12 | <.001 | 0.24 | 0.32 | <.001 |
| Heterosexual | -0.11 | -0.01 | .533 | 0.18 | 0.14 | <.001 | 0.05 | 0.06 | .003 |
| Gay or Lesbian | -1.67 | -0.18 | .012 | 0.16 | 0.11 | <.001 | 0.22 | 0.25 | <.001 |
| Other Sexual Orientations | -0.18 | -0.02 | .463 | 0.22 | 0.16 | <.001 | 0.09 | 0.12 | <.001 |
| Australia | -0.88 | -0.10 | .567 | 0.10 | 0.07 | .137 | 0.22 | 0.25 | .158 |
| Austria | -2.35 | -0.25 | .105 | 0.24 | 0.16 | <.001 | 0.35 | 0.37 | .019 |
| Belgium | 1.52 | 0.17 | .384 | 0.09 | 0.06 | .177 | -0.09 | -0.10 | .613 |
| Canada | -0.65 | -0.08 | .336 | 0.12 | 0.09 | <.001 | 0.14 | 0.18 | .032 |
| Colombia | -0.09 | -0.01 | .918 | 0.17 | 0.13 | <.001 | 0.10 | 0.12 | .236 |
| Chile | -0.75 | -0.08 | .470 | 0.19 | 0.15 | <.001 | 0.18 | 0.20 | .089 |
| Croatia | -1.07 | -0.11 | .163 | 0.31 | 0.21 | <.001 | 0.19 | 0.18 | .018 |
| France | -0.31 | -0.04 | .723 | 0.20 | 0.15 | <.001 | 0.07 | 0.09 | .381 |
| Germany | 0.85 | 0.11 | .197 | 0.17 | 0.12 | <.001 | -0.05 | -0.06 | .464 |
| Hungary | -0.20 | -0.02 | .593 | 0.23 | 0.18 | <.001 | 0.07 | 0.08 | .045 |
| Ireland | -1.57 | -0.18 | .072 | 0.28 | 0.19 | <.001 | 0.24 | 0.29 | .006 |
| Israel | -0.11 | -0.01 | .912 | 0.29 | 0.20 | <.001 | 0.11 | 0.12 | .302 |
| Italy | 1.05 | 0.11 | .111 | 0.29 | 0.24 | <.001 | -0.09 | -0.09 | .195 |
| Malaysia | 1.02 | 0.16 | .226 | 0.07 | 0.08 | .022 | -0.04 | -0.08 | .568 |
| Mexico | -0.09 | -0.01 | .853 | 0.13 | 0.13 | <.001 | 0.09 | 0.14 | .093 |
| New Zealand | 0.45 | 0.05 | .499 | 0.27 | 0.18 | <.001 | 0.04 | 0.06 | .486 |
| North Macedonia | 0.57 | 0.05 | .667 | 0.30 | 0.20 | <.001 | 0.09 | 0.07 | .512 |
| Peru | -0.29 | -0.05 | .581 | 0.13 | 0.12 | <.001 | 0.11 | 0.17 | .043 |
| Poland | -0.73 | -0.09 | .043 | 0.22 | 0.16 | <.001 | 0.13 | 0.17 | <.001 |
| Portugal | -1.27 | -0.16 | .050 | 0.21 | 0.17 | <.001 | 0.23 | 0.28 | <.001 |
| South Africa | 0.77 | 0.08 | .344 | 0.32 | 0.24 | <.001 | 0.00 | 0.00 | .990 |
| South Korea | -0.59 | -0.08 | .349 | 0.10 | 0.13 | <.001 | 0.13 | 0.20 | .026 |
| Spain | -0.29 | -0.03 | .722 | 0.26 | 0.20 | <.001 | 0.07 | 0.08 | .355 |
| Switzerland | -1.44 | -0.16 | .195 | 0.17 | 0.13 | <.001 | 0.23 | 0.27 | .032 |
| United Kingdom | 0.43 | 0.05 | .614 | 0.22 | 0.17 | <.001 | -0.03 | -0.03 | .754 |
| United States | -0.38 | -0.05 | .558 | 0.14 | 0.10 | <.001 | 0.16 | 0.20 | .014 |

*Note*. CSA + AASA = Lifetime Sexual Abuse; PU = SUPPS-Sub Facet Positive Urgency; CSA x PU = Estimate of the relationship between Child Sexual Abuse and Positive Urgency.

### Table M. *Latent Variable Interaction Sensation Seeking Moderating the Relationship Between CSA and ASSIST Scores Between Groups*

| Group | CSA | | | SE | | | CSA x SE | | |
| --- | --- | --- | --- | --- | --- | --- | --- | --- | --- |
|  | *B* | *β* | *p* | *B* | *β* | *p* | *B* | *β* | *p* |
| Cisgender Women | 0.01 | 0.00 | .952 | 0.28 | 0.23 | <.001 | -0.05 | -0.04 | .020 |
| Cisgender Men | 0.21 | 0.02 | .478 | 0.23 | 0.18 | <.001 | -0.03 | -0.03 | .333 |
| Gender-Diverse Individuals | 1.32 | 0.10 | .113 | 0.36 | 0.27 | <.001 | -0.21 | -0.18 | .008 |
| Heterosexual | -0.12 | -0.01 | .514 | 0.22 | 0.19 | <.001 | -0.01 | -0.01 | .570 |
| Gay or Lesbian | -0.26 | -0.02 | .731 | 0.27 | 0.20 | <.001 | -0.02 | -0.02 | .812 |
| Other Sexual Orientations | 0.48 | 0.04 | .162 | 0.32 | 0.24 | <.001 | -0.10 | -0.09 | .002 |
| Australia | -2.34 | -0.18 | .298 | 0.17 | 0.12 | .006 | 0.19 | 0.15 | .390 |
| Austria | -0.75 | -0.06 | .644 | 0.25 | 0.18 | <.001 | 0.11 | 0.10 | .491 |
| Belgium | 3.26 | 0.31 | .035 | 0.14 | 0.12 | .007 | -0.29 | -0.24 | .101 |
| Canada | -0.18 | -0.01 | .859 | 0.27 | 0.21 | <.001 | -0.02 | -0.02 | .805 |
| Colombia | -0.98 | -0.12 | .172 | 0.21 | 0.19 | <.001 | 0.11 | 0.14 | .120 |
| Chile | 1.47 | 0.15 | .119 | 0.25 | 0.21 | <.001 | -0.17 | -0.15 | .107 |
| Croatia | -0.26 | -0.02 | .786 | 0.36 | 0.26 | <.001 | 0.03 | 0.02 | .773 |
| France | -1.65 | -0.16 | .101 | 0.18 | 0.14 | <.001 | 0.20 | 0.19 | .049 |
| Germany | 1.57 | 0.18 | .012 | 0.25 | 0.21 | <.001 | -0.16 | -0.17 | .014 |
| Hungary | -0.01 | 0.00 | .976 | 0.28 | 0.22 | <.001 | 0.00 | 0.00 | .991 |
| Ireland | 1.28 | 0.10 | .331 | 0.32 | 0.23 | <.001 | -0.22 | -0.16 | .104 |
| Israel | -0.63 | -0.06 | .603 | 0.39 | 0.28 | <.001 | 0.03 | 0.02 | .822 |
| Italy | -0.69 | -0.06 | .355 | 0.29 | 0.23 | <.001 | 0.09 | 0.07 | .277 |
| Malaysia | 0.45 | 0.06 | .648 | 0.15 | 0.15 | <.001 | -0.05 | -0.07 | .613 |
| Mexico | 0.33 | 0.05 | .554 | 0.22 | 0.22 | <.001 | -0.06 | -0.08 | .310 |
| New Zealand | 1.85 | 0.14 | .105 | 0.31 | 0.21 | <.001 | -0.20 | -0.16 | .059 |
| North Macedonia | -1.59 | -0.10 | .302 | 0.36 | 0.25 | <.001 | 0.17 | 0.11 | .270 |
| Peru | -0.29 | -0.04 | .577 | 0.20 | 0.21 | <.001 | -0.02 | -0.02 | .760 |
| Poland | 0.15 | 0.01 | .712 | 0.30 | 0.25 | <.001 | -0.04 | -0.04 | .361 |
| Portugal | -0.55 | -0.05 | .529 | 0.27 | 0.20 | <.001 | 0.03 | 0.03 | .724 |
| South Africa | 1.05 | 0.09 | .368 | 0.26 | 0.20 | <.001 | -0.15 | -0.14 | .161 |
| South Korea | 0.55 | 0.08 | .382 | 0.17 | 0.20 | <.001 | -0.08 | -0.12 | .206 |
| Spain | -0.26 | -0.02 | .765 | 0.27 | 0.23 | <.001 | 0.00 | 0.00 | .993 |
| Switzerland | -0.66 | -0.06 | .601 | 0.26 | 0.21 | <.001 | 0.02 | 0.02 | .893 |
| United Kingdom | -0.78 | -0.06 | .602 | 0.30 | 0.22 | <.001 | 0.04 | 0.03 | .800 |
| United States | 0.84 | 0.07 | .372 | 0.25 | 0.19 | <.001 | -0.14 | -0.12 | .114 |

*Note*. CSA = Child Sexual Abuse; SE = SUPPS-Sub Facet Sensation Seeking; CSA x SE = Estimate of the relationship between Child Sexual Abuse and Sensation Seeking.

### Table N. *Latent Variable Interaction Sensation Seeking Moderating the Relationship Between AASA and ASSIST Scores Between Groups*

| Group | AASA | | | SE | | | AASA x SE | | |
| --- | --- | --- | --- | --- | --- | --- | --- | --- | --- |
|  | *B* | *β* | *p* | *B* | *β* | *p* | *B* | *β* | *p* |
| Cisgender Women | 0.58 | 0.08 | <.001 | 0.27 | 0.22 | <.001 | 0.00 | -0.01 | .793 |
| Cisgender Men | 0.26 | 0.03 | .318 | 0.21 | 0.17 | <.001 | 0.04 | 0.05 | .068 |
| Gender-Diverse Individuals | 0.96 | 0.11 | .102 | 0.36 | 0.26 | <.001 | -0.06 | -0.07 | .303 |
| Heterosexual | 0.42 | 0.06 | .003 | 0.22 | 0.18 | <.001 | 0.01 | 0.01 | .683 |
| Gay or Lesbian | 0.92 | 0.11 | .106 | 0.27 | 0.20 | <.001 | -0.02 | -0.02 | .757 |
| Other Sexual Orientations | 0.39 | 0.05 | .092 | 0.31 | 0.23 | <.001 | 0.01 | 0.02 | .529 |
| Australia | -0.08 | -0.01 | .962 | 0.16 | 0.11 | .017 | 0.08 | 0.11 | .566 |
| Austria | -1.22 | -0.16 | .322 | 0.20 | 0.14 | <.001 | 0.20 | 0.28 | .092 |
| Belgium | -1.33 | -0.18 | .216 | 0.08 | 0.07 | .159 | 0.11 | 0.15 | .326 |
| Canada | -0.05 | -0.01 | .927 | 0.26 | 0.20 | <.001 | 0.04 | 0.06 | .471 |
| Colombia | 0.65 | 0.08 | .396 | 0.23 | 0.20 | <.001 | -0.01 | -0.01 | .902 |
| Chile | 1.14 | 0.14 | .197 | 0.22 | 0.19 | <.001 | -0.05 | -0.06 | .584 |
| Croatia | 1.07 | 0.13 | .078 | 0.37 | 0.28 | <.001 | -0.07 | -0.09 | .262 |
| France | 0.90 | 0.12 | .191 | 0.21 | 0.17 | <.001 | -0.05 | -0.06 | .491 |
| Germany | -0.35 | -0.05 | .459 | 0.21 | 0.18 | <.001 | 0.07 | 0.11 | .132 |
| Hungary | 0.28 | 0.04 | .347 | 0.27 | 0.21 | <.001 | 0.03 | 0.03 | .378 |
| Ireland | 1.02 | 0.14 | .177 | 0.32 | 0.24 | <.001 | -0.05 | -0.08 | .459 |
| Israel | 0.68 | 0.08 | .444 | 0.39 | 0.28 | <.001 | -0.03 | -0.04 | .750 |
| Italy | 0.18 | 0.02 | .742 | 0.29 | 0.24 | <.001 | 0.01 | 0.02 | .820 |
| Malaysia | 1.86 | 0.32 | .015 | 0.16 | 0.17 | <.001 | -0.11 | -0.20 | .129 |
| Mexico | 1.01 | 0.15 | .060 | 0.23 | 0.23 | <.001 | -0.09 | -0.13 | .101 |
| New Zealand | 0.15 | 0.02 | .818 | 0.28 | 0.20 | <.001 | 0.02 | 0.03 | .736 |
| North Macedonia | 0.12 | 0.01 | .904 | 0.35 | 0.24 | <.001 | 0.06 | 0.07 | .496 |
| Peru | -0.04 | -0.01 | .941 | 0.20 | 0.21 | <.001 | 0.01 | 0.02 | .785 |
| Poland | 0.61 | 0.09 | .025 | 0.31 | 0.26 | <.001 | -0.03 | -0.05 | .244 |
| Portugal | 1.52 | 0.21 | .020 | 0.29 | 0.22 | <.001 | -0.09 | -0.13 | .145 |
| South Africa | -0.19 | -0.02 | .813 | 0.22 | 0.17 | <.001 | 0.07 | 0.10 | .327 |
| South Korea | -0.24 | -0.04 | .701 | 0.14 | 0.16 | <.001 | 0.08 | 0.12 | .218 |
| Spain | 1.05 | 0.15 | .053 | 0.27 | 0.23 | <.001 | -0.02 | -0.03 | .720 |
| Switzerland | 0.75 | 0.10 | .361 | 0.26 | 0.21 | <.001 | -0.01 | -0.02 | .854 |
| United Kingdom | 0.49 | 0.07 | .564 | 0.28 | 0.21 | <.001 | 0.02 | 0.02 | .837 |
| United States | 0.36 | 0.05 | .590 | 0.23 | 0.18 | <.001 | 0.01 | 0.02 | .834 |

*Note*. AASA = Adult Sexual Abuse; SE = SUPPS-Sub Facet Sensation Seeking; CSA x SE = Estimate of the relationship between Child Sexual Abuse and Sensation Seeking.

### Table O. *Latent Variable Interaction Sensation Seeking Moderating the Relationship Between CSA+AASA and ASSIST Scores Between Groups*

| Group | CSA+ AASA | | | SE | | | CSA +AASA x SE | | |
| --- | --- | --- | --- | --- | --- | --- | --- | --- | --- |
|  | *B* | *β* | *p* | *B* | *β* | *p* | *B* | *β* | *p* |
| Cisgender Women | 0.00 | 0.00 | .987 | 0.25 | 0.21 | <.001 | 0.07 | 0.09 | <.001 |
| Cisgender Men | 0.18 | 0.01 | .602 | 0.22 | 0.17 | <.001 | 0.04 | 0.04 | .175 |
| Gender-Diverse Individuals | -0.54 | -0.06 | .322 | 0.27 | 0.20 | <.001 | 0.16 | 0.21 | .001 |
| Heterosexual | -0.13 | -0.01 | .422 | 0.21 | 0.18 | <.001 | 0.06 | 0.07 | <.001 |
| Gay or Lesbian | -0.04 | 0.00 | .949 | 0.25 | 0.18 | <.001 | 0.05 | 0.06 | .375 |
| Other Sexual Orientations | 0.20 | 0.02 | .400 | 0.29 | 0.22 | <.001 | 0.05 | 0.07 | .017 |
| Australia | 1.69 | 0.19 | .243 | 0.21 | 0.14 | .003 | -0.04 | -0.05 | .774 |
| Austria | 1.29 | 0.14 | .338 | 0.26 | 0.18 | <.001 | -0.03 | -0.03 | .819 |
| Belgium | -0.08 | -0.01 | .954 | 0.09 | 0.08 | .079 | 0.08 | 0.09 | .578 |
| Canada | -0.62 | -0.07 | .347 | 0.23 | 0.18 | <.001 | 0.13 | 0.17 | .033 |
| Colombia | -0.09 | -0.01 | .904 | 0.21 | 0.19 | <.001 | 0.09 | 0.11 | .207 |
| Chile | 0.15 | 0.02 | .879 | 0.20 | 0.17 | <.001 | 0.09 | 0.10 | .344 |
| Croatia | 0.44 | 0.04 | .540 | 0.35 | 0.26 | <.001 | 0.03 | 0.03 | .668 |
| France | -0.61 | -0.07 | .455 | 0.17 | 0.14 | <.001 | 0.11 | 0.14 | .158 |
| Germany | 0.02 | 0.00 | .967 | 0.23 | 0.19 | <.001 | 0.05 | 0.05 | .430 |
| Hungary | -0.16 | -0.02 | .638 | 0.26 | 0.21 | <.001 | 0.08 | 0.08 | .028 |
| Ireland | -1.54 | -0.18 | .077 | 0.25 | 0.18 | <.001 | 0.23 | 0.29 | .005 |
| Israel | -0.33 | -0.04 | .742 | 0.36 | 0.26 | <.001 | 0.13 | 0.15 | .193 |
| Italy | 1.17 | 0.12 | .091 | 0.32 | 0.25 | <.001 | -0.10 | -0.10 | .157 |
| Malaysia | -2.13 | -0.33 | .008 | 0.08 | 0.09 | .009 | 0.24 | 0.43 | <.001 |
| Mexico | -0.37 | -0.06 | .430 | 0.17 | 0.17 | <.001 | 0.12 | 0.19 | .015 |
| New Zealand | 0.20 | 0.02 | .776 | 0.27 | 0.19 | <.001 | 0.08 | 0.10 | .224 |
| North Macedonia | -0.55 | -0.05 | .692 | 0.34 | 0.24 | <.001 | 0.18 | 0.16 | .159 |
| Peru | 0.26 | 0.04 | .568 | 0.18 | 0.19 | <.001 | 0.05 | 0.08 | .270 |
| Poland | -0.26 | -0.03 | .418 | 0.28 | 0.23 | <.001 | 0.08 | 0.10 | .011 |
| Portugal | -0.91 | -0.11 | .211 | 0.22 | 0.17 | <.001 | 0.19 | 0.24 | .008 |
| South Africa | -0.94 | -0.10 | .326 | 0.21 | 0.16 | <.001 | 0.17 | 0.21 | .047 |
| South Korea | -0.18 | -0.03 | .789 | 0.13 | 0.15 | <.001 | 0.10 | 0.15 | .140 |
| Spain | 0.13 | 0.01 | .862 | 0.27 | 0.22 | <.001 | 0.03 | 0.03 | .709 |
| Switzerland | 0.36 | 0.04 | .718 | 0.25 | 0.20 | <.001 | 0.05 | 0.06 | .601 |
| United Kingdom | 0.94 | 0.12 | .287 | 0.32 | 0.24 | <.001 | -0.07 | -0.10 | .376 |
| United States | 0.69 | 0.08 | .315 | 0.21 | 0.16 | <.001 | 0.04 | 0.06 | .475 |

*Note*. CSA + AASA = Lifetime Sexual Abuse; SE = SUPPS-Sub Facet Sensation Seeking; CSA x SE = Estimate of the relationship between Child Sexual Abuse and Sensation Seeking.
