# Supplementary material for: The moderating role of impulsivity in the association between sexual abuse and substance use frequency across countries, gender identities, and sexual orientations
Source: PLoS One. 2026 Sep 30;21(9):e0355986. doi: 10.1371/journal.pone.0355986 (PMC13626376; doi:10.1371/journal.pone.0355986)
Supplement: Supplementary file 5 [file pone.0355986.s006.docx]

**Re: PONE-D-26-01923 – Response to Reviewers and Revised Manuscript**

Dear Dr. Tucker and Editorial Board,

We thank the editors and the reviewer for their careful and constructive evaluation of our manuscript, “The Moderating Role of Impulsivity in the Association between Sexual Abuse and Substance Use Frequency across Countries, Gender Identities, and Sexual Orientations.” We have addressed all points raised and provide detailed responses below. All changes to the manuscript are reflected in the Revised Manuscript with Track Changes file.

**RESPONSES TO JOURNAL REQUIREMENTS**

**Requirement 1: PLOS ONE Style Requirements**

We have reviewed the manuscript against the PLOS ONE style templates and made necessary formatting adjustments to ensure compliance with heading levels, in-text citation style, and file naming conventions.

**Requirement 2: Grant Numbers – Funding Information vs. Financial Disclosure**

We have verified the grant numbers in the Financial Disclosure Statement and confirm these are the correct awards received. The corresponding author (Dr. Potenza) has updated the Funding Information fields in the Editorial Manager submission portal to match the Financial Disclosure Statement exactly. The confirmed grants are:

*DEA: T32DA022975 and K08DA051667*

*BB: FRQSC NP Program (https://doi.org/10.69777/343918) and FRQSC Regroupements stratégiques (https://doi.org/10.69777/281155)*

*LN: ÚNKP-22-3 and ÚNKP-23-4*

*SWK: Kindbridge Research Institute*

*ZD: KKP126835*

*JB: WUN Research Development Fund (RDF) 2021*

**Requirement 3: Role of Funder Statement**

We confirm that the funders had no role in study design, data collection and analysis, decision to publish, or preparation of the manuscript. This statement is included in the Financial Disclosure Statement document and is reproduced here for the editorial team to update in the online submission form:

*The funders had no role in study design, data collection and analysis, decision to publish, or preparation of the manuscript.*

**Requirement 4: Data Availability Statement – Third-Party Contact**

We have updated the Data Sharing Statement to include the contact information for data access requests. The updated statement now reads in full:

*We believe that openly sharing data is important for replication purposes, and in general, we apply this open science practice in our work. However, in this case, it is unfortunately not possible due to ethical constraints. A total of 42 countries are involved in the International Sex Survey, of which 26 are included in the present study. The project was approved by the national or institutional ethics review boards of all participating countries, or, in some cases, the local ethics committees deemed the study exempt from further review, as it had already received approval from the principal investigators’ institutions (https://osf.io/n3k2c/files/osfstorage/636974c6f490ee001cfe45fe). Participating countries and institutions have different ethical regulations regarding data sharing, especially when the dataset includes sensitive topics (e.g., sexuality, pornography use, religiosity). While some institutions support open data sharing, others restrict it to protect participants. As the project aimed to follow a standardized protocol across all participating countries, we adopted the most stringent approach to data sharing (i.e., not sharing the data publicly but instead providing access upon request). Furthermore, based on consultation with a member of the institutional ethics review board at the first author’s institution, public data sharing would require participants’ explicit consent. However, this consent was not included in the original informed consent form, and it is not possible to obtain it retrospectively, as the data were collected anonymously. Requests for data access may be directed to the lead investigator of the International Sex Survey, Beáta Bőthe (beata.bothe@umontreal.ca).*

The corresponding author has also updated this statement in the Editorial Manager submission portal.

**Requirement 5: ISS Consortium – Lead Contact and Individual Members**

The full membership list of the International Sex Survey Consortium with institutional affiliations was already present in the Acknowledgments section of the manuscript. We have added a lead contact line at the top of the Consortium listing in the Acknowledgments (see tracked changes): “Lead contact: Beáta Bőthe, beata.bothe@umontreal.ca.”

**Requirement 6: Full Ethics Statement in Methods**

We have expanded the ethics statement in the Methods section (Procedure subsection) to include the full names of the relevant ethics committees and to confirm the consent procedure. Specifically, we now state that ethics approval in Canada was granted by the Comité d’éthique de la recherche of the Université du Québec à Trois-Rivières (CER-21-276-07.03) and the Comité d’éthique de la recherche en éducation et en psychologie of the Université de Montréal (CEREP, project #2021-2877), and that all participants provided written informed consent electronically prior to completing the survey. A complete list of ethics approvals by country is available at the OSF project page (https://osf.io/n3k2c/files/osfstorage/636974c6f490ee001cfe45fe). Please see the tracked changes in the revised manuscript.

**Requirement 7: Supporting Information Captions**

Captions for all Supporting Information files have been added at the end of the manuscript under a “Supporting Information” heading. In-text citations are consistent with these captions. Please see the tracked changes in the revised manuscript.

**Requirement 8: Identifying Data in Supplemental File**

We thank the editorial team for flagging this. Upon careful review of the supplemental file, we confirmed that it contains only aggregate-level statistical output, specifically, measurement invariance model fit indices (CFI, TLI, RMSEA, chi-square values) across country, gender identity, and sexual orientation groups. There is no participant-level data, no names, no IP addresses, no specific dates, no contact information, and no ID numbers in the file. We have re-uploaded the file and respectfully request that the editorial team clarify what specific information was flagged, as we were unable to identify any identifying information in the document.

**Requirement 9: Reviewer-Recommended Citations**

We have added Coulter et al. (2018) to the Limitations section in response to the bisexual subgroup comment, and all other new citations were added based on the substantive content of our revisions.

**Requirement 10: Reference List Review**

We have reviewed all references for completeness and accuracy. No retracted articles were identified. All references have been verified and corrected where necessary.

**RESPONSES TO REVIEWER #1 COMMENTS**

We sincerely thank Reviewer #1 for their thoughtful feedback. Reviewer comments are shown in italics; our responses follow.

**Comment 1: Rationale for Three Sexual Orientation Categories**

*“Please provide a rationale for why only 3 categories were used for sexual orientation. Bisexual youth often have higher risk behaviors, so it is unclear why other categories for sexual orientation were not included. If the authors are unable to delineate additional categories, this should be noted in the limitations section.”*

Response: We thank the reviewer for this important point. The three-category grouping (heterosexual, gay or lesbian, and other sexual orientations) was necessary to maintain adequate cell sizes for stable structural equation modeling. The ‘other’ category includes bisexual, pansexual, asexual, queer, and other non-heterosexual, non-gay/lesbian identities. The original preregistration specified eight sexual orientation categories, but several had insufficient sample sizes for reliable model estimation. We have added an explicit rationale for this grouping in the Methods section (Sociodemographic Characteristics subsection) and have added a new limitation paragraph acknowledging that this aggregation may obscure meaningful differences between bisexual individuals and other sexual minority subgroups, with reference to Coulter et al. (2018). Please see the tracked changes.

**Comment 2: Table 2 Footnotes**

*“Table 2 – please provide footnotes that inform the reader how to interpret the scores; the tables should be standalone from the text.”*

Response: We agree. The Table 2 note has been substantially expanded to include: the ASSIST-11 score range (0–40) and interpretation of higher scores; each UPPS-P subscale’s item count (4 items) and range (4–16); a statement that higher scores reflect greater impulsivity on all subscales; and a description of the 4-point Likert scale and reverse-coding procedure. Table 2 is now fully interpretable without reference to the main text. Please see the tracked changes.

**Comment 3: SUPPS-P Example Items and Scoring**

*“For the SUPPS-P – please provide an example item for each ‘facet’ of impulsivity; please also provide scoring instructions.”*

Response: We have expanded the SUPPS-P description in the Measures section to include one labeled example item per facet: Lack of Premeditation (“I usually think carefully before doing anything” [reverse-coded]); Lack of Perseverance (“I finish what I start” [reverse-coded]); Sensation Seeking (“I welcome new and exciting experiences and sensations, even if they are a little frightening or unconventional”); Negative Urgency (“When I feel bad, I often do things I later regret”); and Positive Urgency (“When I’m in a great mood, I tend to get carried away easily”). We have also clarified the reverse-coding procedure and stated that subscale scores range from 4 to 16, with higher scores uniformly reflecting greater impulsivity. Please see the tracked changes.

**Comment 4: CSA and Lower Substance Use Frequency**

*“More explanation is needed for why CSA was associated with lower levels of substance use frequency. The authors seem to ‘gloss’ over this a bit in their discussion. For example, the authors mention that impulsivity might be a coping strategy – but how can it be a coping strategy if substance use frequency is increasing.”*

Response: We thank the reviewer for this close reading and fully agree this finding warranted more substantive engagement. We have substantially expanded the relevant Discussion paragraph to address both points. First, we clarify that impulsivity is not itself a coping strategy but rather a dispositional feature that moderates which coping behaviors individuals engage in under emotional distress; individuals high in urgency may be more likely to resort to substance use as a maladaptive response to trauma-related distress, which is consistent with the positive moderation effects observed for AASA and CSA+AASA. Second, we offer four non-mutually exclusive mechanistic explanations for the negative CSA-substance use association: (1) the ASSIST-11 captures only past-3-month frequency rather than lifetime patterns or severity; (2) CSA survivors may develop internalizing rather than externalizing coping styles, particularly when abuse occurred early and was never disclosed; (3) those who experienced CSA without subsequent AASA may represent a relatively resilient subgroup who accessed protective resources; and (4) online recruitment may have underrepresented the most severely affected survivors. We also explicitly note that these findings should not be interpreted as evidence that CSA is protective against substance use. Please see the tracked changes.
