# Supplementary material for: The moderating role of impulsivity in the association between sexual abuse and substance use frequency across countries, gender identities, and sexual orientations
Source: PLoS One. 2026 Sep 30;21(9):e0355986. doi: 10.1371/journal.pone.0355986 (PMC13626376; doi:10.1371/journal.pone.0355986)
Supplement: Peer review history

# Peer review: Decision Letter 0

**DOI:** 10.1371/journal.pone.0355986.r001

**Authors:** Jennifer Tucker

17 May 2026

Dear Dr. Potenza,

Please submit your revised manuscript by Jun 28 2026 11:59PM. If you will need more time than this to complete your revisions, please reply to this message or contact the journal office at plosone@plos.org. When you're ready to submit your revision, log on to https://www.editorialmanager.com/pone/ and select the 'Submissions Needing Revision' folder to locate your manuscript file.

- A letter that responds to each point raised by the academic editor and reviewer(s). You should upload this letter as a separate file labeled 'Response to Reviewers'.
- A marked-up copy of your manuscript that highlights changes made to the original version. You should upload this as a separate file labeled 'Revised Manuscript with Track Changes'.
- An unmarked version of your revised paper without tracked changes. You should upload this as a separate file labeled 'Manuscript'.

If you would like to make changes to your financial disclosure, please include your updated statement in your cover letter. Guidelines for resubmitting your figure files are available below the reviewer comments at the end of this letter.

If applicable, we recommend that you deposit your laboratory protocols in protocols.io to enhance the reproducibility of your results. Protocols.io assigns your protocol its own identifier (DOI) so that it can be cited independently in the future. For instructions see: https://journals.plos.org/plosone/s/submission-guidelines#loc-laboratory-protocols. Additionally, PLOS ONE offers an option for publishing peer-reviewed Lab Protocol articles, which describe protocols hosted on protocols.io. Read more information on sharing protocols at https://plos.org/protocols?utm_medium=editorial-email&utm_source=authorletters&utm_campaign=protocols.

As the corresponding author, your ORCID iD is verified in the submission system and will appear in the published article. PLOS supports the use of ORCID, and we encourage all coauthors to register for an ORCID iD and use it as well. Please encourage your coauthors to verify their ORCID iD within the submission system before final acceptance, as unverified ORCID iDs will not appear in the published article. *Only*  the individual author can complete the verification step; PLOS staff *cannot*  verify ORCID iDs on behalf of authors.

We look forward to receiving your revised manuscript.

Kind regards,

Jennifer Tucker, PhD

Staff Editor

PLOS One

Journal Requirements:

When submitting your revision, we need you to address these additional requirements.

1.Please ensure that your manuscript meets PLOS ONE's style requirements, including those for file naming. The PLOS ONE style templates can be found at

https://journals.plos.org/plosone/s/file?id=wjVg/PLOSOne_formatting_sample_main_body.pdf and

https://journals.plos.org/plosone/s/file?id=ba62/PLOSOne_formatting_sample_title_authors_affiliations.pdf

2. We note that the grant information you provided in the ‘Funding Information’ and ‘Financial Disclosure’ sections do not match.

When you resubmit, please ensure that you provide the correct grant numbers for the awards you received for your study in the ‘Funding Information’ section.

3. Thank you for stating the following financial disclosure:

“Financial Disclosure Statement

The funders had no role in study design, data collection and analysis, decision to publish, or preparation of the manuscript.

Damla Aksen

DEA was supported by the NIDA T32 Neuroimaging Sciences Training in Substance Abuse Program (T32DA022975), and K08DA051667

Beáta Bőthe

BB was supported by the FRQSC – Research Support for New Academics (NP) Program (https://doi.org/10.69777/343918). BB is a researcher of the Centre de Recherche Interdisciplinaire sur les Problèmes Conjugaux et les Agressions Sexuelles, supported by the FRQSC - Regroupements stratégiques / Centre - fonctionnement (https://doi.org/10.69777/281155).

Léna Nagy

LN was supported by the ÚNKP-22-3 and ÚNKP-23-4 New National Excellence Program of the Ministry for Culture and Innovation from the source of the National Research, Development and Innovation Fund.

Shane Kraus

SWK was supported by the Kindbridge Research Institute.

Zsolt Demetrovics

ZD was supported by the Hungarian National Research, Development, and Innovation Office (Grant numbers: KKP126835).

Joël Billieux

JB received support from the WUN Research Development Fund (RDF) 2021”

Please state what role the funders took in the study.  If the funders had no role, please state: "The funders had no role in study design, data collection and analysis, decision to publish, or preparation of the manuscript."

If this statement is not correct you must amend it as needed.

Please include this amended Role of Funder statement in your cover letter; we will change the online submission form on your behalf.

4. In the online submission form, you indicated that your data is available only on request from a third party. Please note that your Data Availability Statement is currently missing the name of the third party contact or institution / contact details for the third party, such as an email address or a link to where data requests can be made. Please update your statement with the missing information.

5. One of the noted authors is a group or consortium “International Sex Survey Consortium” In addition to naming the author group, please list the individual authors and affiliations within this group in the acknowledgments section of your manuscript. Please also indicate clearly a lead author for this group along with a contact email address.

6. Please include your full ethics statement in the ‘Methods’ section of your manuscript file. In your statement, please include the full name of the IRB or ethics committee who approved or waived your study, as well as whether or not you obtained informed written or verbal consent. If consent was waived for your study, please include this information in your statement as well.

7. Please include captions for your Supporting Information files at the end of your manuscript, and update any in-text citations to match accordingly. Please see our Supporting Information guidelines for more information: http://journals.plos.org/plosone/s/supporting-information.

8. We note that there is identifying data in the Supporting Information file “Aksen_ISS_ASSIST11_supplement.docx” Due to the inclusion of these potentially identifying data, we have removed this file from your file inventory. Prior to sharing human research participant data, authors should consult with an ethics committee to ensure data are shared in accordance with participant consent and all applicable local laws.

Data sharing should never compromise participant privacy. It is therefore not appropriate to publicly share personally identifiable data on human research participants. The following are examples of data that should not be shared:

-Name, initials, physical address

-Ages more specific than whole numbers

-Internet protocol (IP) address

-Specific dates (birth dates, death dates, examination dates, etc.)

-Contact information such as phone number or email address

-Location data

-ID numbers that seem specific (long numbers, include initials, titled “Hospital ID”) rather than random (small numbers in numerical order)

Data that are not directly identifying may also be inappropriate to share, as in combination they can become identifying. For example, data collected from a small group of participants, vulnerable populations, or private groups should not be shared if they involve indirect identifiers (such as sex, ethnicity, location, etc.) that may risk the identification of study participants.

Additional guidance on preparing raw data for publication can be found in our Data Policy (https://journals.plos.org/plosone/s/data-availability#loc-human-research-participant-data-and-other-sensitive-data) and in the following article: http://www.bmj.com/content/340/bmj.c181.long

Please remove or anonymize all personal information (<specific identifying information in file to be removed>), ensure that the data shared are in accordance with participant consent, and re-upload a fully anonymized data set. Please note that spreadsheet columns with personal information must be removed and not hidden as all hidden columns will appear in the published file.

9. If the reviewer comments include a recommendation to cite specific previously published works, please review and evaluate these publications to determine whether they are relevant and should be cited. There is no requirement to cite these works unless the editor has indicated otherwise.

10. Please review your reference list to ensure that it is complete and correct. If you have cited papers that have been retracted, please include the rationale for doing so in the manuscript text, or remove these references and replace them with relevant current references. Any changes to the reference list should be mentioned in the rebuttal letter that accompanies your revised manuscript. If you need to cite a retracted article, indicate the article’s retracted status in the References list and also include a citation and full reference for the retraction notice.

Additional Editor Comments:

Please note that we have only been able to secure a single reviewer to assess your manuscript. We are issuing a decision on your manuscript at this point to prevent further delays in the evaluation of your manuscript. Please be aware that the editor who handles your revised manuscript might find it necessary to invite additional reviewers to assess this work once the revised manuscript is submitted. However, we will aim to proceed on the basis of this single review if possible.

[Note: HTML markup is below. Please do not edit.]

Reviewer's Responses to Questions

**Comments to the Author**

1. Is the manuscript technically sound, and do the data support the conclusions?

Reviewer #1: Yes

**********

2. Has the statistical analysis been performed appropriately and rigorously?

Reviewer #1: Yes

**********

3. Have the authors made all data underlying the findings in their manuscript fully available?

The PLOS Data policy

Reviewer #1: Yes

**********

4. Is the manuscript presented in an intelligible fashion and written in standard English?

Reviewer #1: Yes

**********

Reviewer #1: This is a very well written article that examines the association between sexual abuse and substance use frequency, examining impulsivity as a moderator. Before publication, however, the following should be addressed:

• Please provide a rationale for why only 3 categories were used for sexual orientation. Bisexual youth often have higher risk behaviors, so it is unclear why other categories for sexual orientation were not included. If the authors are unable to delineate additional categories, this should be noted in the limitations section.

• Table 2 – please provide footnotes that inform the reader how to interpret the scores; the tables should be standalone from the text

• For the SUPPS-P – please provide an example item for each “facet” of impulsivity; please also provide scoring instructions

• More explanation is needed for why CSA was associated with lower levels of substance use frequency. The authors seem to “gloss” over this a bit in their discussion. For example, the authors mention that impulsivity might be a coping strategy – but how can it be a coping strategy if substance use frequency is increasing.

**********

what does this mean?). If published, this will include your full peer review and any attached files.

If you choose “no”, your identity will remain anonymous but your review may still be made public.

**Do you want your identity to be public for this peer review?**  For information about this choice, including consent withdrawal, please see our Privacy Policy

Reviewer #1: No

**********

[NOTE: If reviewer comments were submitted as an attachment file, they will be attached to this email and accessible via the submission site. Please log into your account, locate the manuscript record, and check for the action link "View Attachments". If this link does not appear, there are no attachment files.]

To ensure your figures meet our technical requirements, please review our figure guidelines: https://journals.plos.org/plosone/s/figures

You may also use PLOS’s free figure tool, NAAS, to help you prepare publication quality figures: https://journals.plos.org/plosone/s/figures#loc-tools-for-figure-preparation.

NAAS will assess whether your figures meet our technical requirements by comparing each figure against our figure specifications.

# Author comment: Author response to Decision Letter 1

**DOI:** 10.1371/journal.pone.0355986.r002

9 Jul 2026

Re: PONE-D-26-01923 – Response to Reviewers and Revised Manuscript

Dear Dr. Tucker and Editorial Board,

We thank the editors and the reviewer for their careful and constructive evaluation of our manuscript, “The Moderating Role of Impulsivity in the Association between Sexual Abuse and Substance Use Frequency across Countries, Gender Identities, and Sexual Orientations.” We have addressed all points raised and provide detailed responses below. All changes to the manuscript are reflected in the Revised Manuscript with Track Changes file.

RESPONSES TO JOURNAL REQUIREMENTS

Requirement 1: PLOS ONE Style Requirements

We have reviewed the manuscript against the PLOS ONE style templates and made necessary formatting adjustments to ensure compliance with heading levels, in-text citation style, and file naming conventions.

Requirement 2: Grant Numbers – Funding Information vs. Financial Disclosure

We have verified the grant numbers in the Financial Disclosure Statement and confirm these are the correct awards received. The corresponding author (Dr. Potenza) has updated the Funding Information fields in the Editorial Manager submission portal to match the Financial Disclosure Statement exactly. The confirmed grants are:

DEA: T32DA022975 and K08DA051667

BB: FRQSC NP Program (https://doi.org/10.69777/343918) and FRQSC Regroupements stratégiques (https://doi.org/10.69777/281155)

LN: ÚNKP-22-3 and ÚNKP-23-4

SWK: Kindbridge Research Institute

ZD: KKP126835

JB: WUN Research Development Fund (RDF) 2021

Requirement 3: Role of Funder Statement

We confirm that the funders had no role in study design, data collection and analysis, decision to publish, or preparation of the manuscript. This statement is included in the Financial Disclosure Statement document and is reproduced here for the editorial team to update in the online submission form:

The funders had no role in study design, data collection and analysis, decision to publish, or preparation of the manuscript.

Requirement 4: Data Availability Statement – Third-Party Contact

We have updated the Data Sharing Statement to include the contact information for data access requests. The updated statement now reads in full:

We believe that openly sharing data is important for replication purposes, and in general, we apply this open science practice in our work. However, in this case, it is unfortunately not possible due to ethical constraints. A total of 42 countries are involved in the International Sex Survey, of which 26 are included in the present study. The project was approved by the national or institutional ethics review boards of all participating countries, or, in some cases, the local ethics committees deemed the study exempt from further review, as it had already received approval from the principal investigators’ institutions (https://osf.io/n3k2c/files/osfstorage/636974c6f490ee001cfe45fe). Participating countries and institutions have different ethical regulations regarding data sharing, especially when the dataset includes sensitive topics (e.g., sexuality, pornography use, religiosity). While some institutions support open data sharing, others restrict it to protect participants. As the project aimed to follow a standardized protocol across all participating countries, we adopted the most stringent approach to data sharing (i.e., not sharing the data publicly but instead providing access upon request). Furthermore, based on consultation with a member of the institutional ethics review board at the first author’s institution, public data sharing would require participants’ explicit consent. However, this consent was not included in the original informed consent form, and it is not possible to obtain it retrospectively, as the data were collected anonymously. Requests for data access may be directed to the lead investigator of the International Sex Survey, Beáta Bőthe (beata.bothe@umontreal.ca).

The corresponding author has also updated this statement in the Editorial Manager submission portal.

Requirement 5: ISS Consortium – Lead Contact and Individual Members

The full membership list of the International Sex Survey Consortium with institutional affiliations was already present in the Acknowledgments section of the manuscript. We have added a lead contact line at the top of the Consortium listing in the Acknowledgments (see tracked changes): “Lead contact: Beáta Bőthe, beata.bothe@umontreal.ca.”

Requirement 6: Full Ethics Statement in Methods

We have expanded the ethics statement in the Methods section (Procedure subsection) to include the full names of the relevant ethics committees and to confirm the consent procedure. Specifically, we now state that ethics approval in Canada was granted by the Comité d’éthique de la recherche of the Université du Québec à Trois-Rivières (CER-21-276-07.03) and the Comité d’éthique de la recherche en éducation et en psychologie of the Université de Montréal (CEREP, project #2021-2877), and that all participants provided written informed consent electronically prior to completing the survey. A complete list of ethics approvals by country is available at the OSF project page (https://osf.io/n3k2c/files/osfstorage/636974c6f490ee001cfe45fe). Please see the tracked changes in the revised manuscript.

Requirement 7: Supporting Information Captions

Captions for all Supporting Information files have been added at the end of the manuscript under a “Supporting Information” heading. In-text citations are consistent with these captions. Please see the tracked changes in the revised manuscript.

Requirement 8: Identifying Data in Supplemental File

We thank the editorial team for flagging this. Upon careful review of the supplemental file, we confirmed that it contains only aggregate-level statistical output, specifically, measurement invariance model fit indices (CFI, TLI, RMSEA, chi-square values) across country, gender identity, and sexual orientation groups. There is no participant-level data, no names, no IP addresses, no specific dates, no contact information, and no ID numbers in the file. We have re-uploaded the file and respectfully request that the editorial team clarify what specific information was flagged, as we were unable to identify any identifying information in the document.

Requirement 9: Reviewer-Recommended Citations

We have added Coulter et al. (2018) to the Limitations section in response to the bisexual subgroup comment, and all other new citations were added based on the substantive content of our revisions.

Requirement 10: Reference List Review

We have reviewed all references for completeness and accuracy. No retracted articles were identified. All references have been verified and corrected where necessary.

RESPONSES TO REVIEWER #1 COMMENTS

We sincerely thank Reviewer #1 for their thoughtful feedback. Reviewer comments are shown in italics; our responses follow.

Comment 1: Rationale for Three Sexual Orientation Categories

“Please provide a rationale for why only 3 categories were used for sexual orientation. Bisexual youth often have higher risk behaviors, so it is unclear why other categories for sexual orientation were not included. If the authors are unable to delineate additional categories, this should be noted in the limitations section.”

Response: We thank the reviewer for this important point. The three-category grouping (heterosexual, gay or lesbian, and other sexual orientations) was necessary to maintain adequate cell sizes for stable structural equation modeling. The ‘other’ category includes bisexual, pansexual, asexual, queer, and other non-heterosexual, non-gay/lesbian identities. The original preregistration specified eight sexual orientation categories, but several had insufficient sample sizes for reliable model estimation. We have added an explicit rationale for this grouping in the Methods section (Sociodemographic Characteristics subsection) and have added a new limitation paragraph acknowledging that this aggregation may obscure meaningful differences between bisexual individuals and other sexual minority subgroups, with reference to Coulter et al. (2018). Please see the tracked changes.

Comment 2: Table 2 Footnotes

“Table 2 – please provide footnotes that inform the reader how to interpret the scores; the tables should be standalone from the text.”

Response: We agree. The Table 2 note has been substantially expanded to include: the ASSIST-11 score range (0–40) and interpretation of higher scores; each UPPS-P subscale’s item count (4 items) and range (4–16); a statement that higher scores reflect greater impulsivity on all subscales; and a description of the 4-point Likert scale and reverse-coding procedure. Table 2 is now fully interpretable without reference to the main text. Please see the tracked changes.

Comment 3: SUPPS-P Example Items and Scoring

“For the SUPPS-P – please provide an example item for each ‘facet’ of impulsivity; please also provide scoring instructions.”

Response: We have expanded the SUPPS-P description in the Measures section to include one labeled example item per facet: Lack of Premeditation (“I usually think carefully before doing anything” [reverse-coded]); Lack of Perseverance (“I finish what I start” [reverse-coded]); Sensation Seeking (“I welcome new and exciting experiences and sensations, even if they are a little frightening or unconventional”); Negative Urgency (“When I feel bad, I often do things I later regret”); and Positive Urgency (“When I’m in a great mood, I tend to get carried away easily”). We have also clarified the reverse-coding procedure and stated that subscale scores range from 4 to 16, with higher scores uniformly reflecting greater impulsivity. Please see the tracked changes.

Comment 4: CSA and Lower Substance Use Frequency

“More explanation is needed for why CSA was associated with lower levels of substance use frequency. The authors seem to ‘gloss’ over this a bit in their discussion. For example, the authors mention that impulsivity might be a coping strategy – but how can it be a coping strategy if substance use frequency is increasing.”

Response: We thank the reviewer for this close reading and fully agree this finding warranted more substantive engagement. We have substantially expanded the relevant Discussion paragraph to address both points. First, we clarify that impulsivity is not itself a coping strategy but rather a dispositional feature that moderates which coping behaviors individuals engage in under emotional distress; individuals high in urgency may be more likely to resort to substance use as a maladaptive response to trauma-related distress, which is consistent with the positive moderation effects observed for AASA and CSA+AASA. Second, we offer four non-mutually exclusive mechanistic explanations for the negative CSA-substance use association: (1) the ASSIST-11 captures only past-3-month frequency rather than lifetime patterns or severity; (2) CSA survivors may develop internalizing rather than externalizing coping styles, particularly when abuse occurred early and was never disclosed; (3) those who experienced CSA without subsequent AASA may represent a relatively resilient subgroup who accessed protective resources; and (4) online recruitment may have underrepresented the most severely affected survivors. We also explicitly note that these findings should not be interpreted as evidence that CSA is protective against substance use. Please see the tracked changes.

Attachment Submitted filename: Response_to_Reviewers.docx

# Editor report: Decision Letter 1

**DOI:** 10.1371/journal.pone.0355986.r003

**Authors:** Emily Lund

28 Jul 2026

The Moderating Role of Impulsivity in the Association between Sexual Abuse and Substance Use Frequency across Countries, Gender Identities, and Sexual Orientations

PONE-D-26-01923R1

Dear Dr. Potenza,

We’re pleased to inform you that your manuscript has been judged scientifically suitable for publication and will be formally accepted for publication once it meets all outstanding technical requirements.

Within one week, you’ll receive an e-mail detailing the required amendments. When these have been addressed, you’ll receive a formal acceptance letter and your manuscript will be scheduled for publication.

An invoice will be generated when your article is formally accepted. Please note, if your institution has a publishing partnership with PLOS and your article meets the relevant criteria, all or part of your publication costs will be covered. Please make sure your user information is up-to-date by logging into Editorial Manager at Editorial Manager® and clicking the ‘Update My Information' link at the top of the page. For questions related to billing, please contact billing support.

If your institution or institutions have a press office, please notify them about your upcoming paper to help maximize its impact. If they’ll be preparing press materials, please inform our press team as soon as possible -- no later than 48 hours after receiving the formal acceptance. Your manuscript will remain under strict press embargo until 2 pm Eastern Time on the date of publication. For more information, please contact onepress@plos.org.

Kind regards,

Emily Lund

Academic Editor

PLOS One

Additional Editor Comments (optional):

Reviewers' comments:

# Editor report: Acceptance letter

**DOI:** 10.1371/journal.pone.0355986.r004

**Authors:** Emily Lund

PONE-D-26-01923R1

PLOS One

Dear Dr. Potenza,

I'm pleased to inform you that your manuscript has been deemed suitable for publication in PLOS One. Congratulations! Your manuscript is now being handed over to our production team.

At this stage, our production department will prepare your paper for publication. This includes ensuring the following:

* All references, tables, and figures are properly cited

* All relevant supporting information is included in the manuscript submission,

* There are no issues that prevent the paper from being properly typeset

You will receive further instructions from the production team, including instructions on how to review your proof when it is ready. Please keep in mind that we are working through a large volume of accepted articles, so please give us a few days to review your paper and let you know the next and final steps.

Lastly, if your institution or institutions have a press office, please let them know about your upcoming paper now to help maximize its impact. If they'll be preparing press materials, please inform our press team within the next 48 hours. Your manuscript will remain under strict press embargo until 2 pm Eastern Time on the date of publication. For more information, please contact onepress@plos.org.

You will receive an invoice from PLOS for your publication fee after your manuscript has reached the completed accept phase. If you receive an email requesting payment before acceptance or for any other service, this may be a phishing scheme. Learn how to identify phishing emails and protect your accounts at https://explore.plos.org/phishing.

If we can help with anything else, please email us at customercare@plos.org.

Thank you for submitting your work to PLOS One and supporting open access.

Kind regards,

PLOS One Editorial Office Staff

on behalf of

Dr. Emily Lund

Academic Editor

PLOS One
